# Supplementary material for: Olea europaea L. Root Endophyte Bacillus velezensis OEE1 Counteracts Oomycete and Fungal Harmful Pathogens and Harbours a Large Repertoire of Secreted and Volatile Metabolites and Beneficial Functional Genes
Source: Microorganisms. 2019 Sep 3;7(9):314. doi: 10.3390/microorganisms7090314 (PMC6780883; doi:10.3390/microorganisms7090314)

Table S1. antiSMASH hits for analyzed genomes in this study.

Strain clusters	Type	Length (bp)	Most similar known clusters	Predicted core clusters
**CAU B946**				
Cluster 1	Nrps	65407	Surfactin biosynthetic gene cluster (78% of genes show similarity)	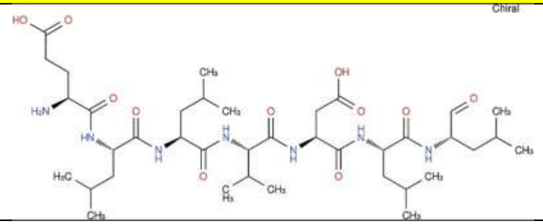
Cluster 2	Otherks	41244	Butirosin biosynthetic gene cluster (7% of genes show similarity)	-
Cluster 3	Terpene	20740	-	-
Cluster 4	Transatpks	85884	Macrolactin biosynthetic gene cluster (100% of genes show similarity)	-
Cluster 5	Transatpks-Nrps	102692	Bacillaene biosynthetic gene cluster (100% of genes show similarity)	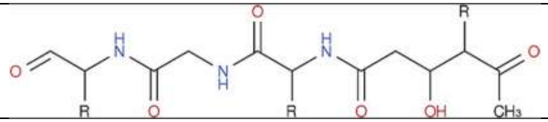
Cluster 6	Transatpks-Nrps	137829	Fengycin biosynthetic gene cluster (100% of genes show similarity)	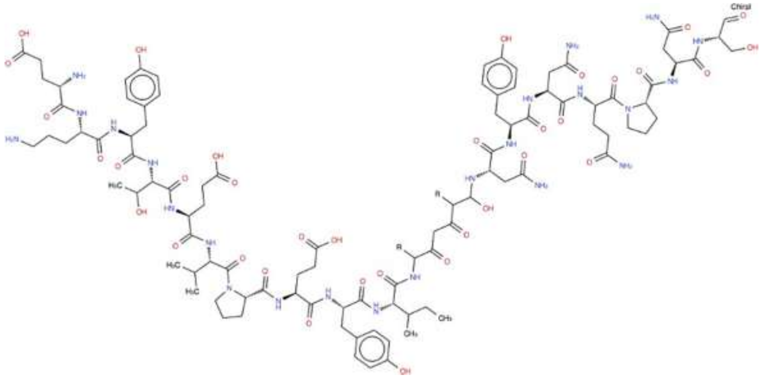
Cluster 7	Terpene	21883	-	-
Cluster 8	T3pks	41109	-	-
Cluster 9	Transatpks	95444	Difficidin biosynthetic gene cluster (100% of genes show similarity)	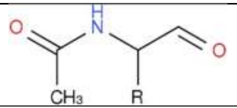

Table S1. Continued.

Strain clusters	Type	Length (bp)	Most similar known clusters	Predicted core clusters
**CAU B946**				
Cluster 10	Bacteriocin-Nrps	66793	Bacillibactin biosynthetic gene cluster (100% of genes show similarity)	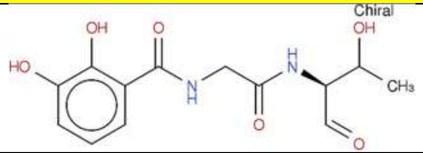
Cluster 11	Other	41418	Bacilysin biosynthetic gene cluster (100% of genes show similarity)	-

Table S1. Continued.

Strain clusters	Type	Length (bp)	Most similar known clusters	Predicted core clusters
**M27**				
Cluster 1	Other	41418	Bacilysin biosynthetic gene cluster (100% of genes show similarity)	-
Cluster 2	Bacteriocin-Nrps	51793	Bacillibactin biosynthetic gene cluster (100% of genes show similarity)	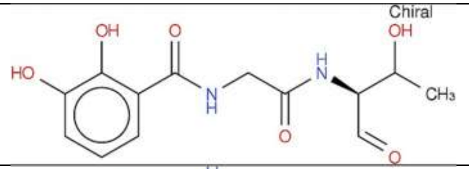
Cluster 3	Transatpks	100450	Difficidin biosynthetic gene cluster (100% of genes show similarity)	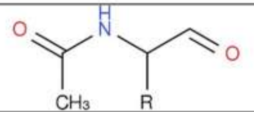
Cluster 4	T3pks	41109	-	-
Cluster 5	Terpene	21883	-	-
Cluster 6	Nrps	22704	Plipastatin biosynthetic gene cluster (30% of genes show similarity)	-
Cluster 7	Nrps	14947	Fengycin biosynthetic gene cluster (26% of genes show similarity)	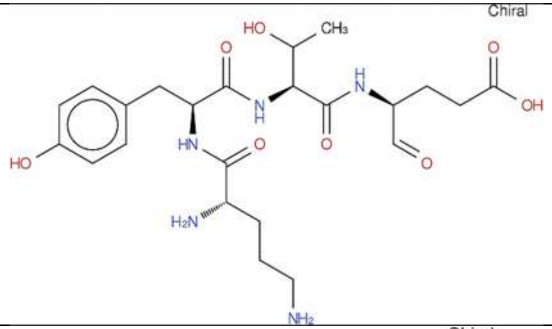
Cluster 8	Nrps	10147	Fengycin biosynthetic gene cluster (13% of genes show similarity)	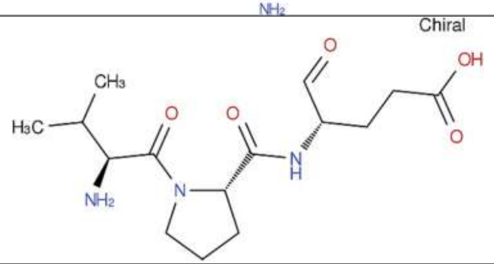

Table S1. Continued.

Strain clusters	Type	Length (bp)	Most similar known clusters	Predicted core clusters
**M27**				
Cluster 9	Nrps-Transatpks	87564	Fengycin biosynthetic gene cluster (80% of genes show similarity)	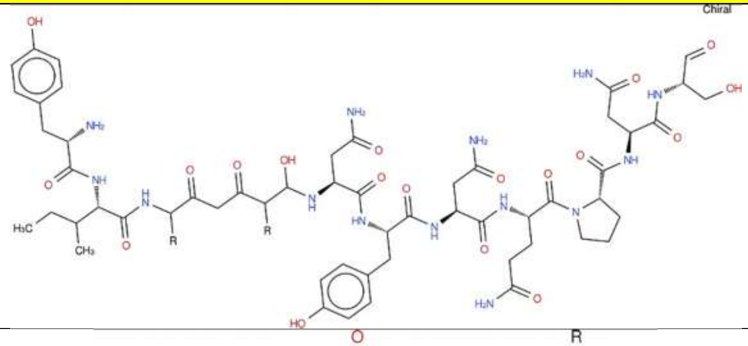
Cluster 10	Transatpks-Nrps	102707	Bacillaene biosynthetic gene cluster (100% of genes show similarity)	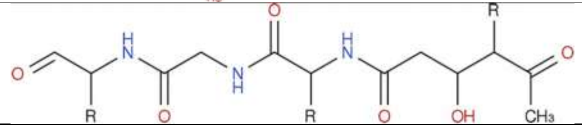
Cluster 11	Transatpks	85802	Macrolactin biosynthetic gene cluster (100% of genes show similarity)	-
Cluster 12	Lantipeptide	28889	-	-
Cluster 13	Terpene	20740	-	-
Cluster 14	Otherks	41244	Butirosin biosynthetic gene cluster (7% of genes show similarity)	-
Cluster 15	Nrps	25161	Surfactin biosynthetic gene cluster (34% of genes show similarity)	-
Cluster 16	Nrps	38342	Surfactin biosynthetic gene cluster (52% of genes show similarity)	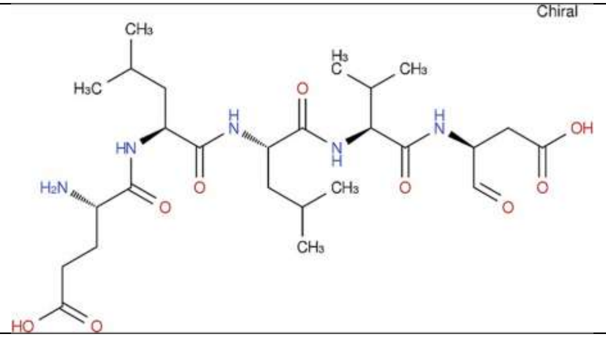

Table S1. Continued.

Strain clusters	Type	Length (bp)	Most similar known clusters	Predicted core clusters
**AS43.3**				
Cluster 1	Nrps	65407	Surfactin biosynthetic gene cluster (91% of genes show similarity)	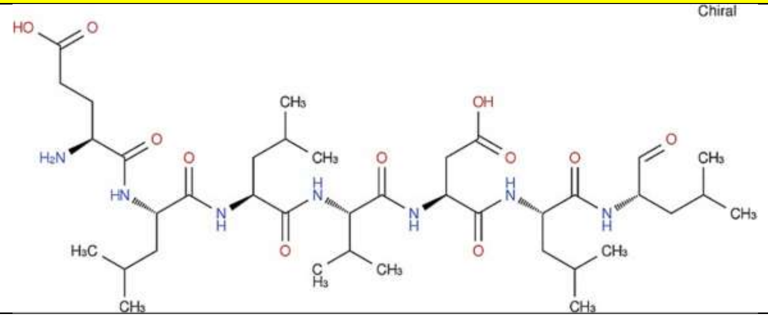
Cluster 2	Ladderane	41217	-	-
Cluster 3	Otherks	41244	Butirosin biosynthetic gene cluster (7% of genes show similarity)	-
Cluster 4	Terpene	20740	-	-
Cluster 5	Transatpks	85896	Macrolactin biosynthetic gene cluster (100% of genes show similarity)	-
Cluster 6	Transatpks-Nrps	102678	Bacillaene biosynthetic gene cluster (100% of genes show similarity)	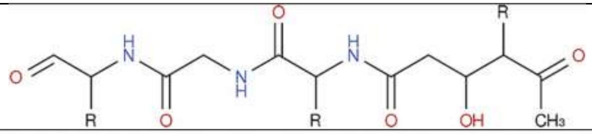
Cluster 7	Transatpks-Nrps	139085	Fengycin biosynthetic gene cluster (100% of genes show similarity)	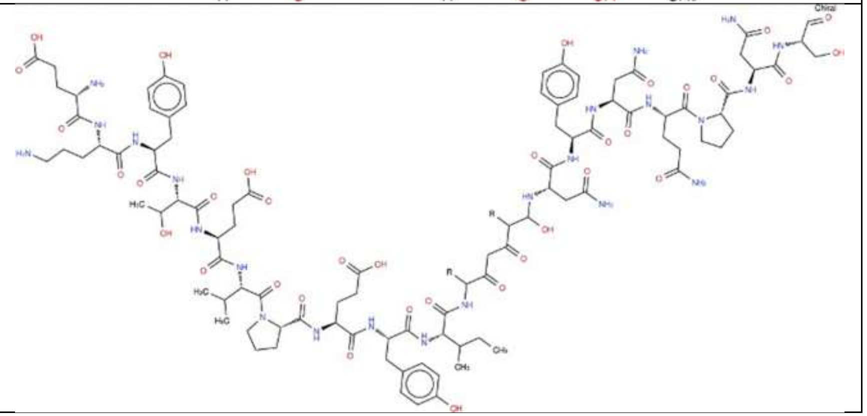

Table S1. Continued.

Strain clusters	Type	Length (bp)	Most similar known clusters	Predicted core clusters
**AS43.3**				
Cluster 8	Terpene	21883	-	-
Cluster 9	T3pks	41109	-	-
Cluster 10	Transatpks	100449	Difficidin biosynthetic gene cluster (100% of genes show similarity)	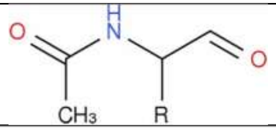
Cluster 11	Bacteriocin-Nrps	66790	Bacillibactin biosynthetic gene cluster (100% of genes show similarity)	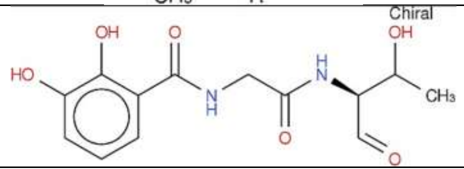
Cluster 12	Other	41418	Bacilysin biosynthetic gene cluster (100% of genes show similarity)	-

Table S1. Continued.

Strain clusters	Type	Length (bp)	Most similar known clusters	Predicted core clusters
**FZB42**				
Cluster 1	Nrps	65407	Surfactin biosynthetic gene cluster (91% of genes show similarity)	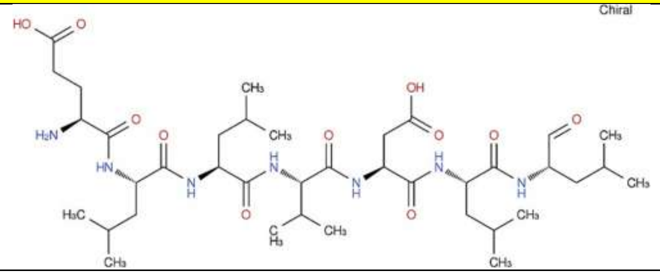
Cluster 2	Otherks	41244	-	-
Cluster 3	Terpene	20740	-	-
Cluster 4	Transatpks	85899	Macrolactin biosynthetic gene cluster (100% of genes show similarity)	-
Cluster 5	Transatpks-Nrps	102683	Bacillaene biosynthetic gene cluster (100% of genes show similarity)	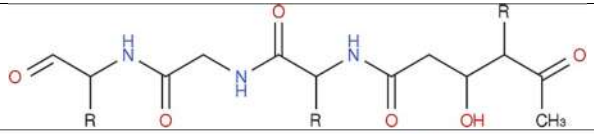
Cluster 6	Transatpks-Nrps	137825	Fengycin biosynthetic gene cluster (100% of genes show similarity)	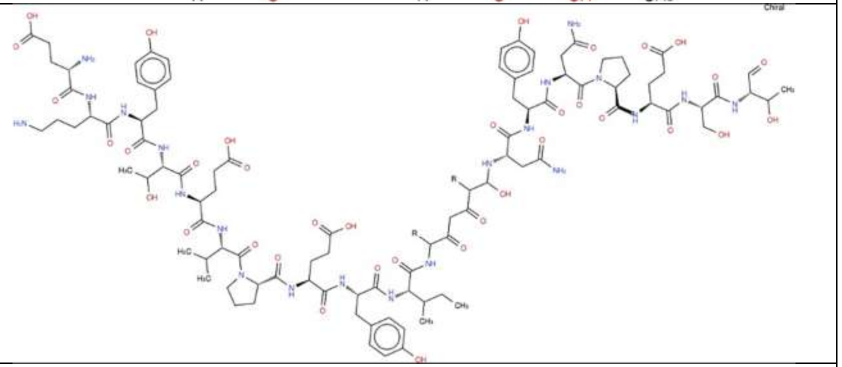
Cluster 7	Terpene	21883	-	-
Cluster 8	T3pks	41109	-	-
Cluster 9	Transatpks	100447	Difficidin biosynthetic gene cluster (100% of genes show similarity)	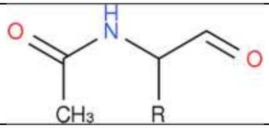

Table S1. Continued.

Strain clusters	Type	Length (bp)	Most similar known clusters	Predicted core clusters
**FZB42**				
Cluster 10	Nrps	55810	-	-
Cluster 18	Bacteriocin-Nrps	66791	Bacillibactin biosynthetic gene cluster (100% of genes show similarity)	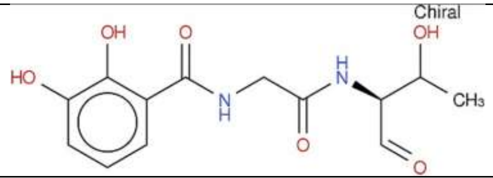
Cluster 19	Other	41418	Bacilysin biosynthetic gene cluster (100% of genes show similarity)	-

Table S1. Continued.

Strain clusters	Type	Length (bp)	Most similar known clusters	Predicted core clusters
**AQGM01000001**				
Cluster 1	Nrps	23248	-	-
Cluster 2	Other	41418	Bacilysin biosynthetic gene cluster (100% of genes show similarity)	-
Cluster 3	Terpene	20740	-	-
Cluster 4	Otherks	41244	Butirosin biosynthetic gene cluster (7% of genes show similarity)	-
Cluster 5	Transatpks-Nrps	100101	Bacillaene biosynthetic gene cluster (100% of genes show similarity)	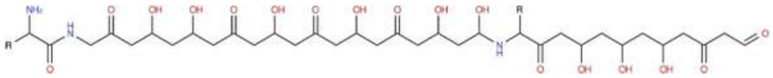
Cluster 6	Transatpks	45842	Difficidin biosynthetic gene cluster (53% of genes show similarity)	-
Cluster 7	T3pks	41109	-	-
Cluster 8	Transatpks	85902	Macrolactin biosynthetic gene cluster (100% of genes show similarity)	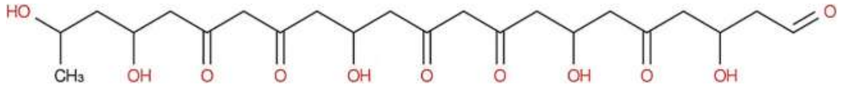
Cluster 9	Phosphonate	40902	-	-
Cluster 10	Bacteriocin-Nrps	51536	Bacillibactin biosynthetic gene cluster (100% of genes show similarity)	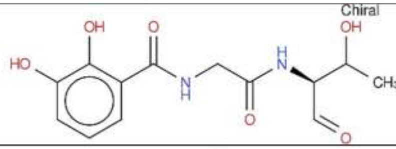
Cluster 11	Transatpks-Nrps	88037	Fengycin biosynthetic gene cluster (86% of genes show similarity)	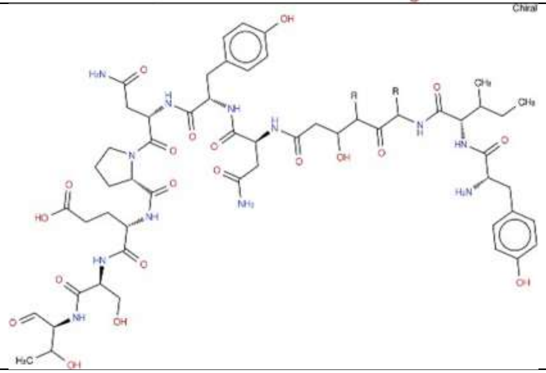

Table S1. Continued.

Strain clusters	Type	Length (bp)	Most similar known clusters	Predicted core clusters
**AQGM01000001**				
Cluster 12	Transatpks	28292	Difficidin biosynthetic gene cluster (46% of genes show similarity)	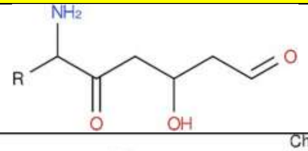
Cluster 13	Nrps	28032	Surfactin biosynthetic gene cluster (47% of genes show similarity)	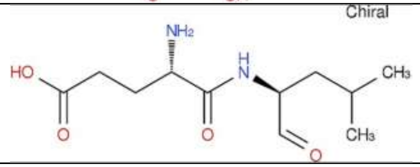
Cluster 14	Terpene	21883	-	-
Cluster 15	Nrps	21780	Plipastatin biosynthetic gene cluster (30% of genes show similarity)	-
Cluster 16	Transatpks	23101	Difficidin biosynthetic gene cluster (26% of genes show similarity)	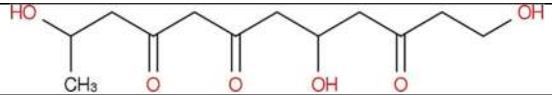
Cluster 17	Nrps	15078	Surfactin biosynthetic gene cluster (39% of genes show similarity)	-
Cluster 18	Nrps	13069	Fengycin biosynthetic gene cluster (20% of genes show similarity)	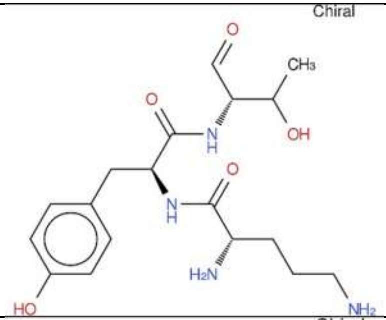
Cluster 19	Nrps	9559	Surfactin biosynthetic gene cluster (8% of genes show similarity)	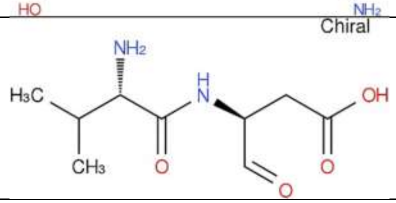

Table S1. Continued.

Strain clusters	Type	Length (bp)	Most similar known clusters	Predicted core clusters
**AQGM01000001**				
Cluster 20	Nrps	9098	Fengycin biosynthetic gene cluster (13% of genes show similarity)	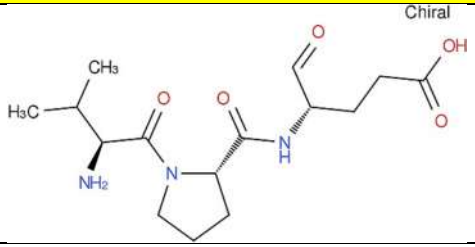
Cluster 21	Nrps	6895	-	-
Cluster 22	Nrps	6499	-	-

Table S1. Continued.

Strain clusters	Type	Length (bp)	Most similar known clusters	Predicted core clusters
**NAU-B3**				
Cluster 1	Nrps	65408	Surfactin biosynthetic gene cluster (82% of genes show similarity)	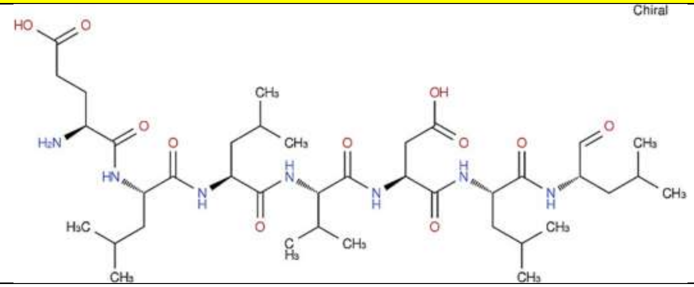
Cluster 2	Phosphonate	40902	-	-
Cluster 3	Transatpks	100444	Difficidin biosynthetic gene cluster (100% of genes show similarity)	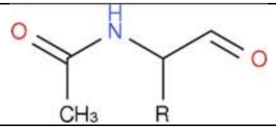
Cluster 4	T3pks	41109	-	-
Cluster 5	Terpene	21883	-	-
Cluster 6	Transatpks-Nrps	137831	Fengycin biosynthetic gene cluster (100% of genes show similarity)	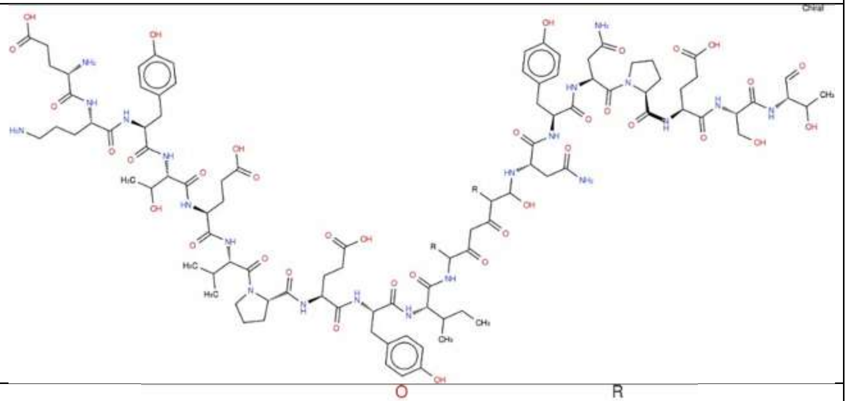
Cluster 7	Transatpks-Nrps	102683	Bacillaene biosynthetic gene cluster (92% of genes show similarity)	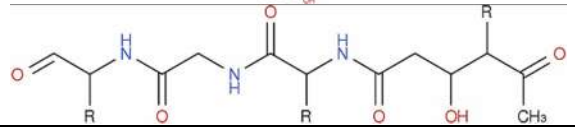
Cluster 8	Transatpks	78518	Macrolactin biosynthetic gene cluster (90% of genes show similarity)	-

Table S1. Continued.

Strain clusters	Type	Length (bp)	Most similar known clusters	Predicted core clusters
**NAU-B3**				
Cluster 9	Terpene	20740	-	-
Cluster 10	Otherks	41244	Butirosin biosynthetic gene cluster (7% of genes show similarity)	-
Cluster 11	Bacteriocin-Nrps	66795	Bacillibactin biosynthetic gene cluster (100% of genes show similarity)	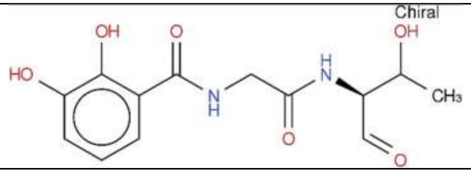
Cluster 19	Nrps	65342	-	-
Cluster 20	Other	41418	Bacilysin biosynthetic gene cluster (100% of genes show similarity)	-

Table S1. Continued.

Strain clusters	Type	Length (bp)	Most similar known clusters	Predicted core clusters
**SK19.001**				
Cluster 1	Other	41418	Bacilysin biosynthetic gene cluster (100% of genes show similarity)	-
Cluster 2	Transatpks-Nrps	82374	Bacillaene biosynthetic gene cluster (100% of genes show similarity)	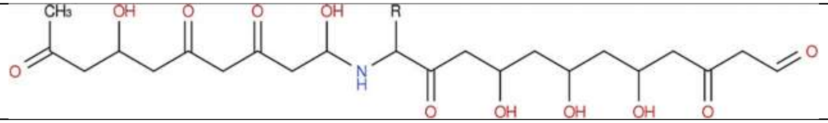
Cluster 3	Transatpks	85902	Macrolactin biosynthetic gene cluster (100% of genes show similarity)	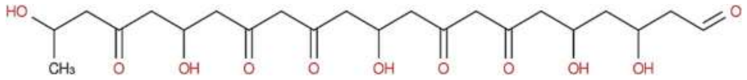
Cluster 4	Otherks	41244	Butirosin biosynthetic gene cluster (7% of genes show similarity)	-
Cluster 5	Terpene	20740	-	-
Cluster 6	Lantipeptide	28889	-	-
Cluster 7	Terpene	21883	-	-
Cluster 8	Transatpks	45822	Difficidin biosynthetic gene cluster (53% of genes show similarity)	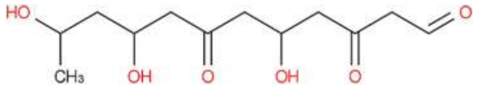
Cluster 9	Transatpks	28322	Difficidin biosynthetic gene cluster (46% of genes show similarity)	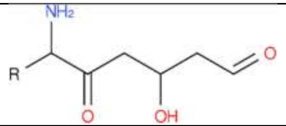
Cluster 10	Nrps	25189	Surfactin biosynthetic gene cluster (39% of genes show similarity)	-
Cluster 11	Nrps-Transatpks	87842	Fengycin biosynthetic gene cluster (80% of genes show similarity)	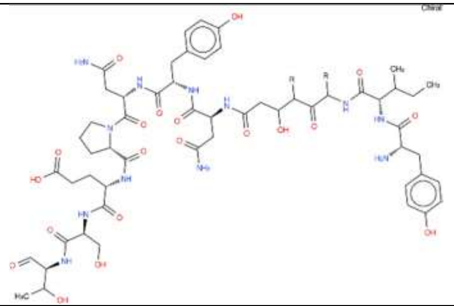

Table S1. Continued.

Strain clusters	Type	Length (bp)	Most similar known clusters	Predicted core clusters
**SK19.001**				
Cluster 12	Bacteriocin-Nrps	51522	Bacillibactin biosynthetic gene cluster (100% of genes show similarity)	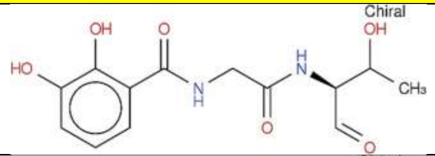
Cluster 13	Nrps	27628	Surfactin biosynthetic gene cluster (47% of genes show similarity)	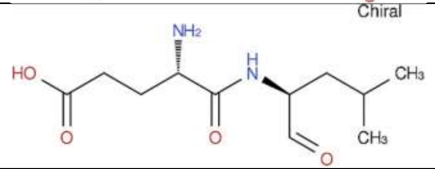
Cluster 14	Transatpks	23032	Difficidin biosynthetic gene cluster (26% of genes show similarity)	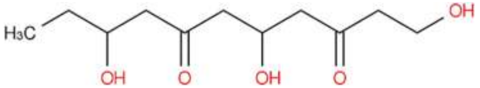
Cluster 15	Nrps	21896	Fengycin biosynthetic gene cluster (20% of genes show similarity)	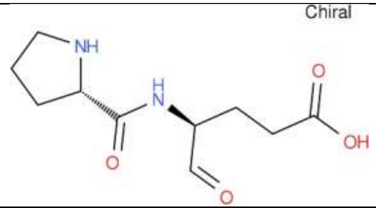
Cluster 16	Nrps	14781	Plipastatin biosynthetic gene cluster (38% of genes show similarity)	-
Cluster 17	Nrps	8947	Surfactin biosynthetic gene cluster (8% of genes show similarity)	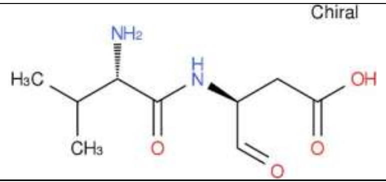
Cluster 18	Bacteriocin	6571	-	-
Cluster 19	Nrps	1883	-	-

Table S1. Continued.

Strain clusters	Type	Length (bp)	Most similar known clusters	Predicted core clusters
**9912D**				
Cluster 1	Nrps	65407	Surfactin biosynthetic gene cluster (82% of genes show similarity)	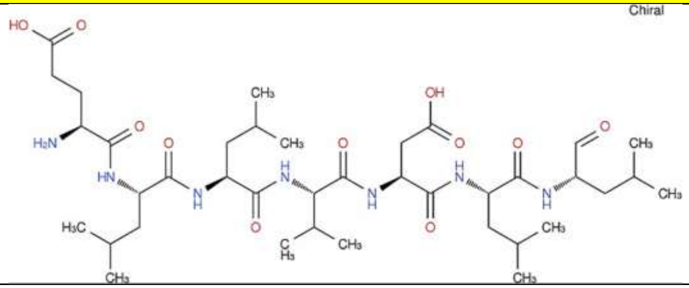
Cluster 2	Otherks	41244	Butirosin biosynthetic gene cluster (7% of genes show similarity)	-
Cluster 3	Terpene	20740	-	-
Cluster 4	Lantipeptide	28889	Micrococcin P1 biosynthetic gene cluster (8% of genes show similarity)	-
Cluster 5	Transatpks	85902	Macrolactin biosynthetic gene cluster (100% of genes show similarity)	-
Cluster 6	Transatpks-Nrps	83665	Bacillaene biosynthetic gene cluster (100% of genes show similarity)	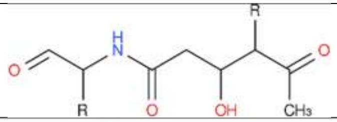
Cluster 7	Transatpks-Nrps	136487	Fengycin biosynthetic gene cluster (93% of genes show similarity)	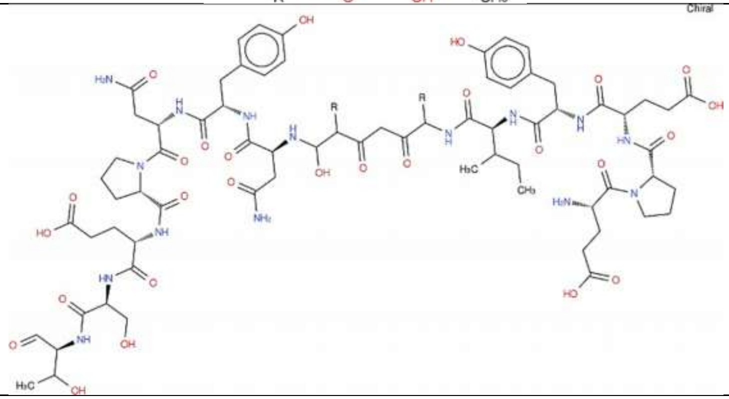

Table S1. Continued.

Strain clusters	Type	Length (bp)	Most similar known clusters	Predicted core clusters
**9912D**				
Cluster 8	Terpene	21883	-	-
Cluster 9	Transatpks	100447	Difficidin biosynthetic gene cluster (100% of genes show similarity)	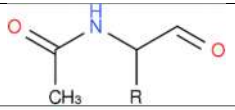
Cluster 10	Bacteriocin-Nrps	66791	Bacillibactin biosynthetic gene cluster (100% of genes show similarity)	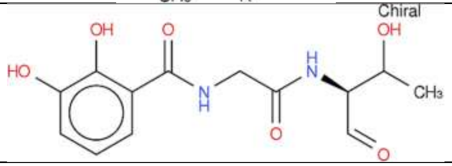
Cluster 11	Other	41418	Bacilysin biosynthetic gene cluster (100% of genes show similarity)	-

Table S1. Continued.

Strain clusters	Type	Length (bp)	Most similar known clusters	Predicted core clusters
**JJ-D34**				
Cluster 1	Nrps	65407	Surfactin biosynthetic gene cluster (78% of genes show similarity)	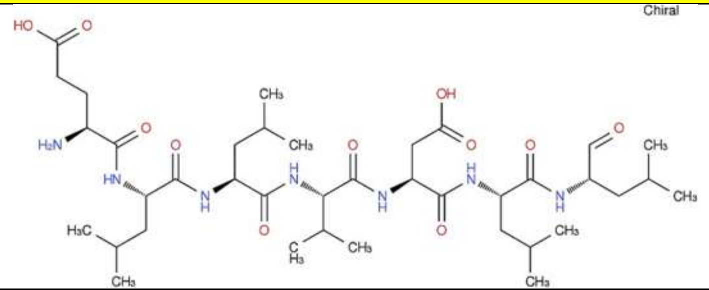
Cluster 2	Thiopeptide	45069	Kijanimicin biosynthetic gene cluster (4% of genes show similarity)	-
Cluster 3	Otherks	41244	Butirosin biosynthetic gene cluster (7% of genes show similarity)	-
Cluster 4	Terpene	20740	-	-
Cluster 5	Transatpks	85887	Macrolactin biosynthetic gene cluster (100% of genes show similarity)	-
Cluster 6	Transatpks-Nrps	102692	Bacillaene biosynthetic gene cluster (100% of genes show similarity)	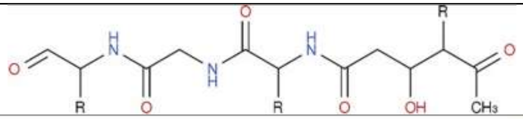
Cluster 7	Transatpks-Nrps	137832	Fengycin biosynthetic gene cluster (100% of genes show similarity)	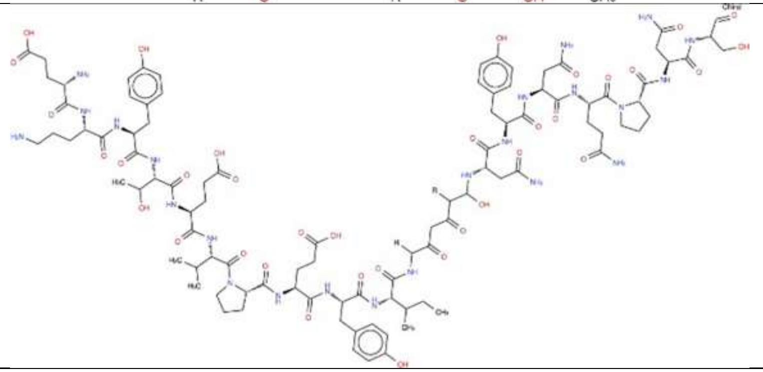

Table S1. Continued.

Strain clusters	Type	Length (bp)	Most similar known clusters	Predicted core clusters
**JJ-D34**				
Cluster 8	Terpene	21883	-	-
Cluster 9	T3pks	41109	-	-
Cluster 10	Transatpks	100450	Difficidin biosynthetic gene cluster (100% of genes show similarity)	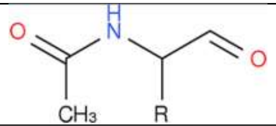
Cluster 11	Bacteriocin-Nrps	66792	Bacillibactin biosynthetic gene cluster (100% of genes show similarity)	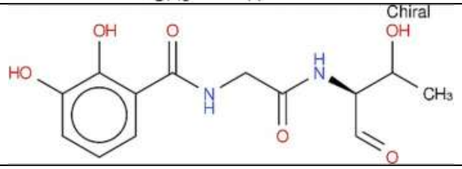
Cluster 18	Other	41418	Bacilysin biosynthetic gene cluster (100% of genes show similarity)	-

Table S1. Continued.

Strain clusters	Type	Length (bp)	Most similar known clusters	Predicted core clusters
**CBMB205**				
Cluster 1	Other	41418	Bacilysin biosynthetic gene cluster (100% of genes show similarity)	-
Cluster 2	Bacteriocin-Nrps	66791	Bacillibactin biosynthetic gene cluster (100% of genes show similarity)	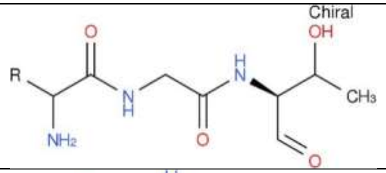
Cluster 3	Transatpks	100453	Difficidin biosynthetic gene cluster (100% of genes show similarity)	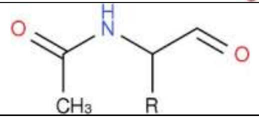
Cluster 4	T3pks	41100	-	-
Cluster 5	Terpene	21883	-	-
Cluster 6	Transatpks-Nrps	137801	Fengycin biosynthetic gene cluster (100% of genes show similarity)	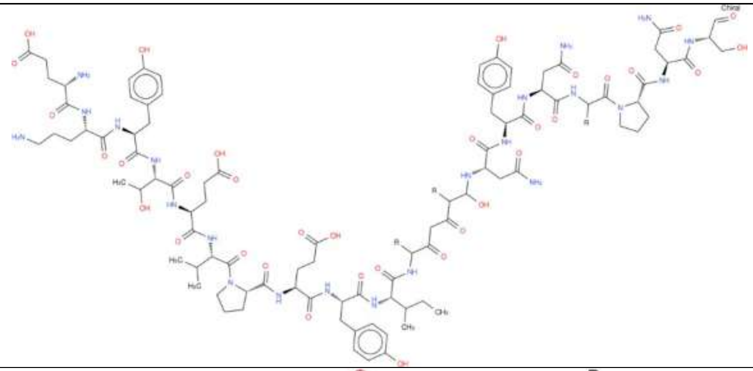
Cluster 7	Transatpks-Nrps	102674	Bacillaene biosynthetic gene cluster (100% of genes show similarity)	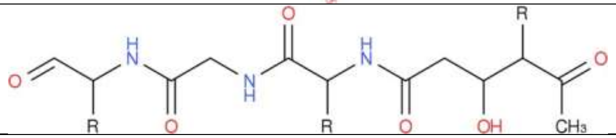
Cluster 8	Transatpks	85905	Macrolactin biosynthetic gene cluster (100% of genes show similarity)	-
Cluster 9	Lantipeptide	28888	-	-
Cluster 10	Terpene	20740	-	-

Table S1. Continued.

Strain clusters	Type	Length (bp)	Most similar known clusters	Predicted core clusters
**CBMB205**				
Cluster 11	Otherks	41244	Butirosin biosynthetic gene cluster (7% of genes show similarity)	-
Cluster 12	Nrps	65407	Surfactin biosynthetic gene cluster (82% of genes show similarity)	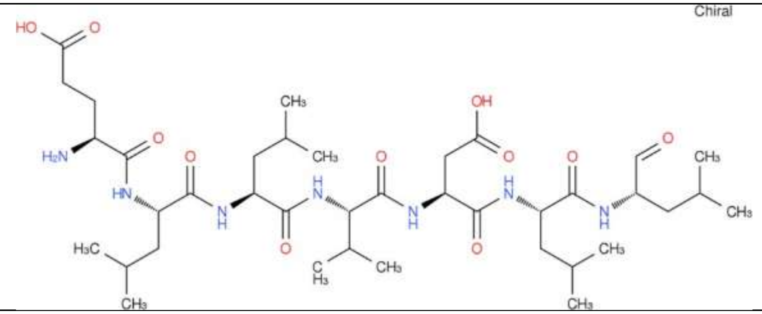

Table S1. Continued.

Strain clusters	Type	Length (bp)	Most similar known clusters	Predicted core clusters
**FKM10**				
Cluster 1	Other	41418	Bacilysin biosynthetic gene cluster (100% of genes show similarity)	-
Cluster 2	Bacteriocin-Nrps	51791	Bacillibactin biosynthetic gene cluster (100% of genes show similarity)	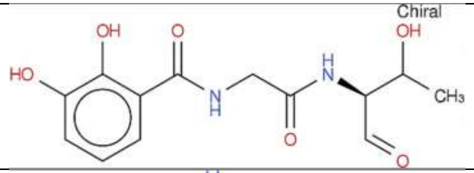
Cluster 3	Transatpks	100453	Difficidin biosynthetic gene cluster (100% of genes show similarity)	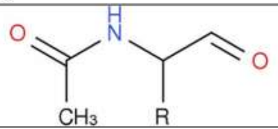
Cluster 4	T3pks	41109	-	-
Cluster 5	Terpene	21883	-	-
Cluster 6	Nrps-Transatpks	137801	Fengycin biosynthetic gene cluster (100% of genes show similarity)	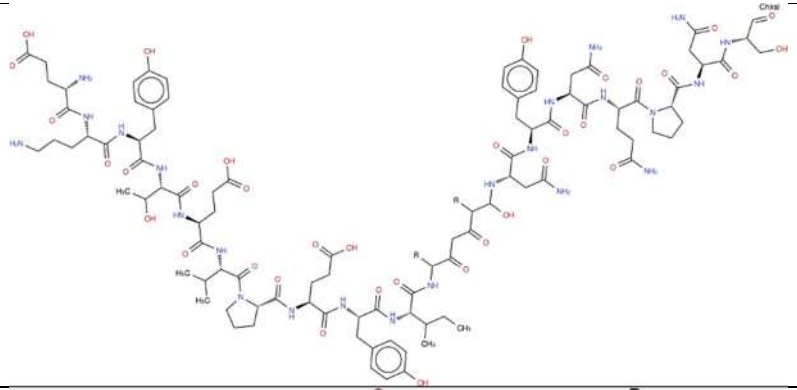
Cluster 7	Transatpks-Nrps	102674	Bacillaene biosynthetic gene cluster (100% of genes show similarity)	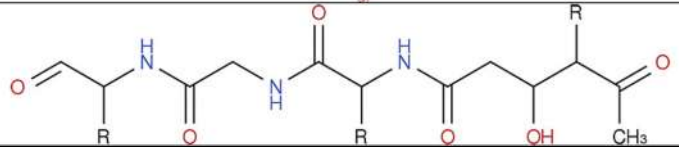
Cluster 8	Transatpks	85905	Macrolactin biosynthetic gene cluster (90% of genes show similarity)	-
Cluster 9	Lantipeptide	28888	-	-

Table S1. Continued.

Strain clusters	Type	Length (bp)	Most similar known clusters	Predicted core clusters
**FKM10**				
Cluster 10	Terpene	20740	-	-
Cluster 11	Otherks	41244	Butirosin biosynthetic gene cluster (7% of genes show similarity)	-
Cluster 12	Nrps	65407	Surfactin biosynthetic gene cluster (78% of genes show similarity)	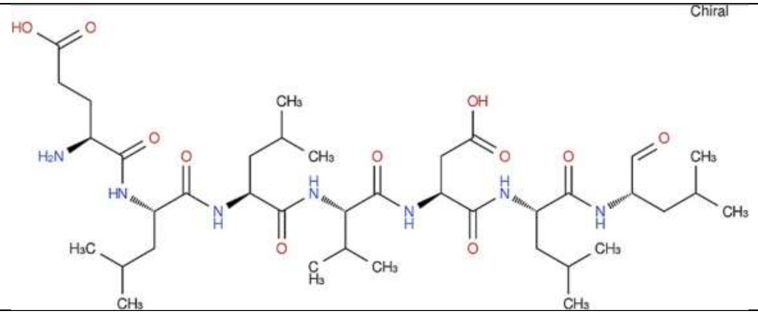

Table S1. Continued.

Strain clusters	Type	Length (bp)	Most similar known clusters	Predicted core clusters
**K26**				
Cluster 1	Other	41418	Bacilysin biosynthetic gene cluster (100% of genes show similarity)	-
Cluster 2	Transatpks	85902	Macrolactin biosynthetic gene cluster (100% of genes show similarity)	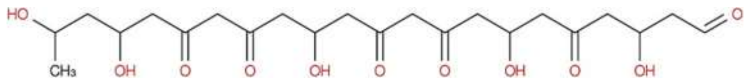
Cluster 3	Transatpks-Nrps	82375	Bacillaene biosynthetic gene cluster (100% of genes show similarity)	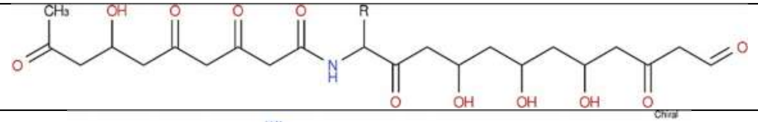
Cluster 4	Transatpks-Nrps	111670	Fengycin biosynthetic gene cluster (93% of genes show similarity)	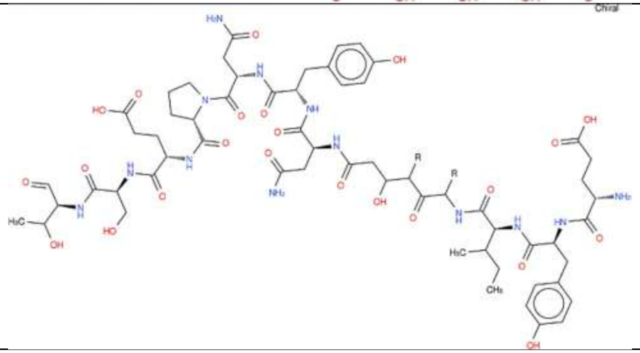
Cluster 5	Terpene	21883	-	-
Cluster 6	Transatpks	100444	Difficidin biosynthetic gene cluster (100% of genes show similarity)	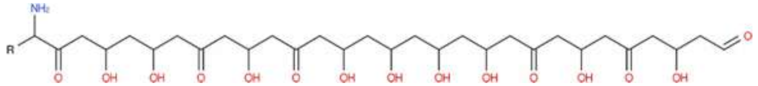
Cluster 7	Lantipeptide	28889	-	-
Cluster 8	Terpene	20740	-	-
Cluster 9	Otherks	41244	Butirosin biosynthetic gene cluster (7% of genes show similarity)	-
Cluster 10	Nrps	27949	Surfactin biosynthetic gene cluster (47% of genes show similarity)	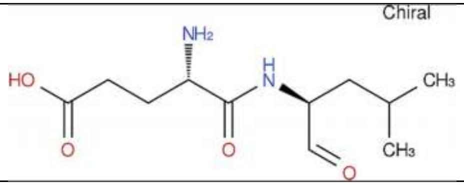

Table S1. Continued.

Strain clusters	Type	Length (bp)	Most similar known clusters	Predicted core clusters
**K26**				
Cluster 11	Nrps	26083	Surfactin biosynthetic gene cluster (39% of genes show similarity)	-
Cluster 12	Ladderane	41217	-	-
Cluster 13	Bacteriocin-Nrps	66792	Bacillibactin biosynthetic gene cluster (100% of genes show similarity)	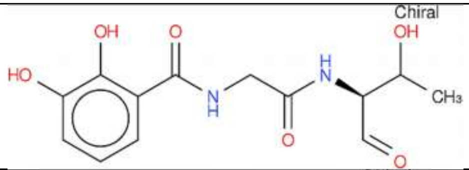
Cluster 14	Nrps	10159	Surfactin biosynthetic gene cluster (8% of genes show similarity)	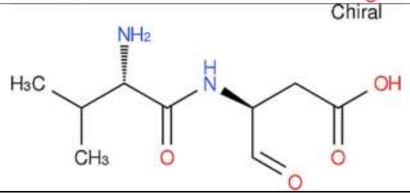

Table S1. Continued.

Strain clusters	Type	Length (bp)	Most similar known clusters	Predicted core clusters
**B26**				
Cluster 1	Transatpks	75787	Difficidin biosynthetic gene cluster (86% of genes show similarity)	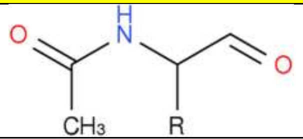
Cluster 2	T3pks	41109	-	-
Cluster 3	Terpene	21883	-	-
Cluster 4	Nrps	48404	Plipastatin biosynthetic gene cluster (53% of genes show similarity)	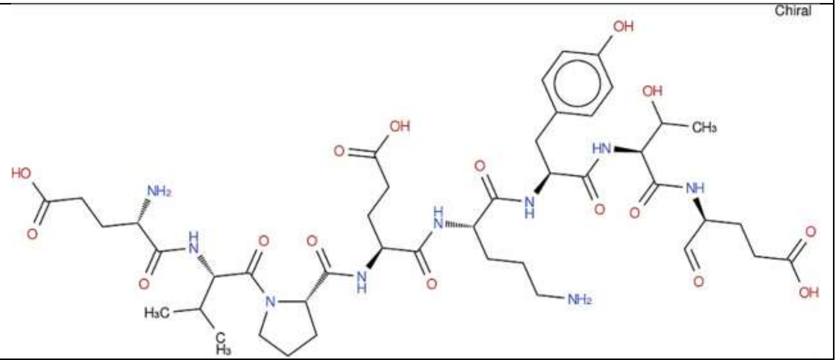
Cluster 5	Nrps	24757	-	-
Cluster 6	Other	41418	Bacilysin biosynthetic gene cluster (100% of genes show similarity)	-
Cluster 7	Terpene	20740	-	-
Cluster 8	Transatpks	85896	Macrolactin biosynthetic gene cluster (100% of genes show similarity)	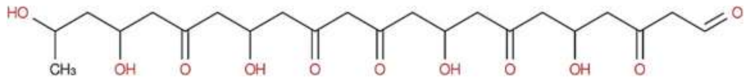
Cluster 9	Nrps-Transatpks	102689	Bacillaene biosynthetic gene cluster (100% of genes show similarity)	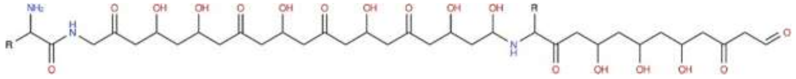
Cluster 10	Nrps-Transatpks	88616	Fengycin biosynthetic gene cluster (86% of genes show similarity)	
Cluster 11	Nrps	35338	-	-

Table S1. Continued.

Strain clusters	Type	Length (bp)	Most similar known clusters	Predicted core clusters
**B26**				
Cluster 12	Nrps	37080	Surfactin biosynthetic gene cluster (47% of genes show similarity)	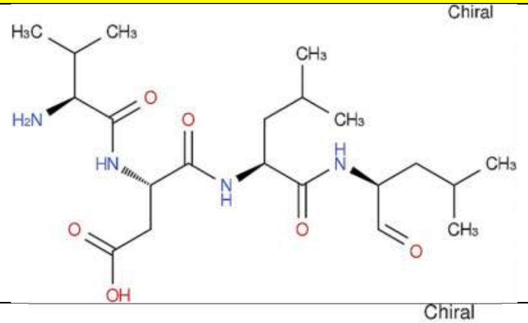
Cluster 13	Nrps	28237	Surfactin biosynthetic gene cluster (47% of genes show similarity)	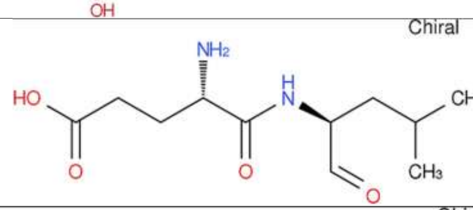
Cluster 14	Bacteriocin-Nrps	51794	Bacillibactin biosynthetic gene cluster (100% of genes show similarity)	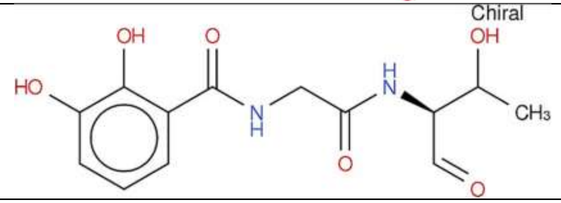
Cluster 15	Otherks	41244	Butirosin biosynthetic gene cluster (7% of genes show similarity)	-
Cluster 16	Transatpks	24629	Difficidin biosynthetic gene cluster (26% of genes show similarity)	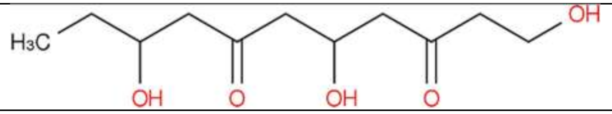
Cluster 17	Nrps	7256	-	-

Table S1. Continued.

Strain clusters	Type	Length (bp)	Most similar known clusters	Predicted core clusters
**LS69**				
Cluster 1	Nrps	65407	Surfactin biosynthetic gene cluster (82% of genes show similarity)	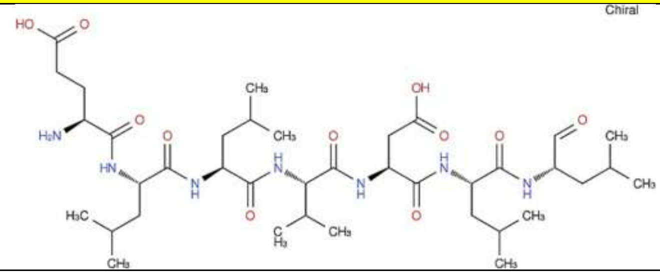
Cluster 2	Otherks	41244	Butirosin biosynthetic gene cluster (7% of genes show similarity)	-
Cluster 3	Terpene	20740	-	-
Cluster 4	Lantipeptide	28888	-	-
Cluster 5	Transatpks	85905	Macrolactin biosynthetic gene cluster (100% of genes show similarity)	-
Cluster 6	Nrps-Transatpks	102674	Bacillaene biosynthetic gene cluster (100% of genes show similarity)	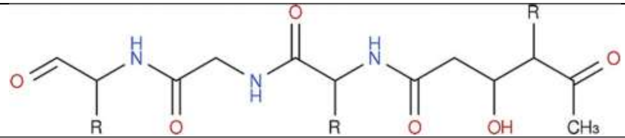
Cluster 7	Nrps-Transatpks	137801	Fengycin biosynthetic gene cluster (100% of genes show similarity)	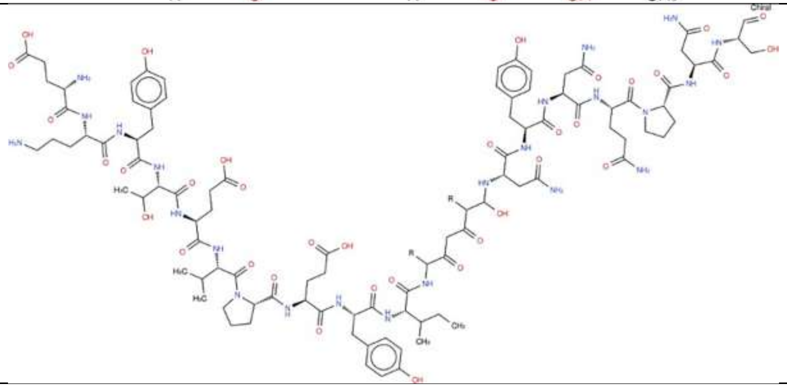
Cluster 8	Terpene	21883	-	-
Cluster 9	T3pks	41109	-	-

Table S1. Continued.

Strain clusters	Type	Length (bp)	Most similar known clusters	Predicted core clusters
**LS69**				
Cluster 10	Transatpks	100453	Difficidin biosynthetic gene cluster (100% of genes show similarity)	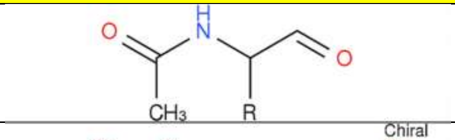
Cluster 11	Bacteriocin-Nrps	66791	Bacillibactin biosynthetic gene cluster (100% of genes show similarity)	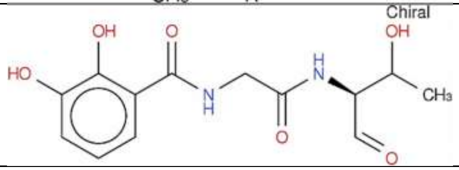
Cluster 12	Other	41418	Bacilysin biosynthetic gene cluster (100% of genes show similarity)	-

Table S1. Continued.

Strain clusters	Type	Length (bp)	Most similar known clusters	Predicted core clusters
**KACC 13105**				
Cluster 1	Nrps	36146	Surfactin biosynthetic gene cluster (39% of genes show similarity)	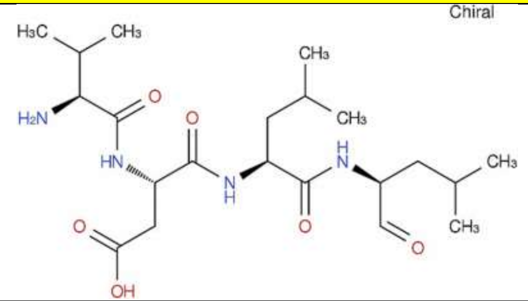
Cluster 2	Transatpks	85905	Macrolactin biosynthetic gene cluster (100% of genes show similarity)	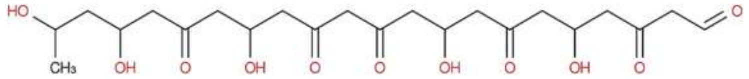
Cluster 3	Terpene	21883	-	-
Cluster 4	Nrps	23186	Plipastatin biosynthetic gene cluster (38% of genes show similarity)	-
Cluster 5	Microcin	4598	-	-
Cluster 6	Nrps-Transatpks	114098	Fengycin biosynthetic gene cluster (93% of genes show similarity)	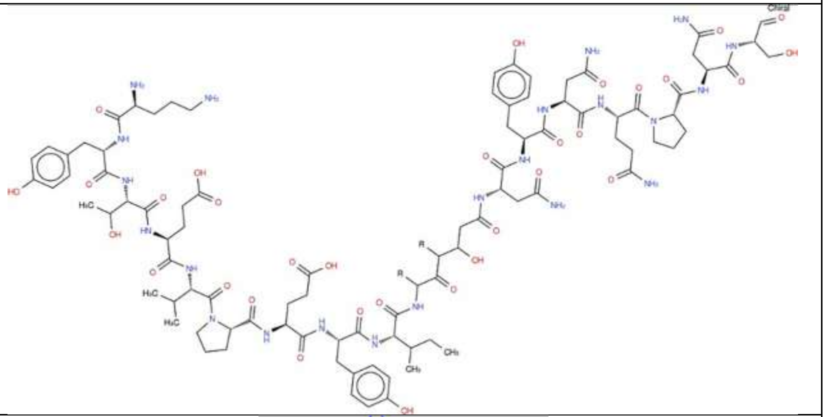
Cluster 7	Transatpks	100453	Difficidin biosynthetic gene cluster (93% of genes show similarity)	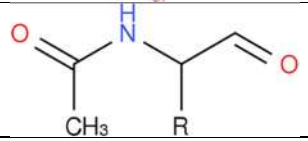

Table S1. Continued.

Strain clusters	Type	Length (bp)	Most similar known clusters	Predicted core clusters
**KACC 13105**				
Cluster 8	T3pks	41109	-	-
Cluster 9	Nrps-Bacteriocin	66791	Bacillibactin biosynthetic gene cluster (100% of genes show similarity)	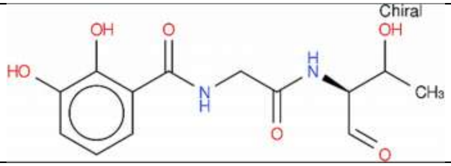
Cluster 10	Other	41418	Bacilysin biosynthetic gene cluster (100% of genes show similarity)	-
Cluster 11	Nrps-Transatpks	102674	Bacillaene biosynthetic gene cluster (100% of genes show similarity)	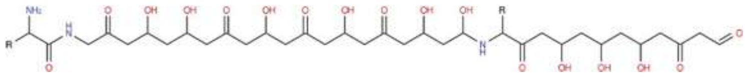
Cluster 12	Nrps	29170	Surfactin biosynthetic gene cluster (47% of genes show similarity)	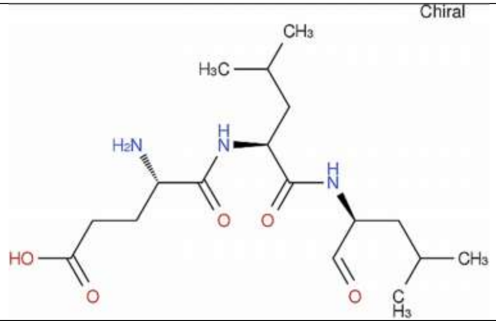
Cluster 13	Otherks	41244	-	-
Cluster 14	Terpene	20740	-	-
Cluster 15	Lantipeptide	28888	-	-

Table S1. Continued.

Strain clusters	Type	Length (bp)	Most similar known clusters	Predicted core clusters
**RC218**				
Cluster 1	Nrps	65407	Surfactin biosynthetic gene cluster (91% of genes show similarity)	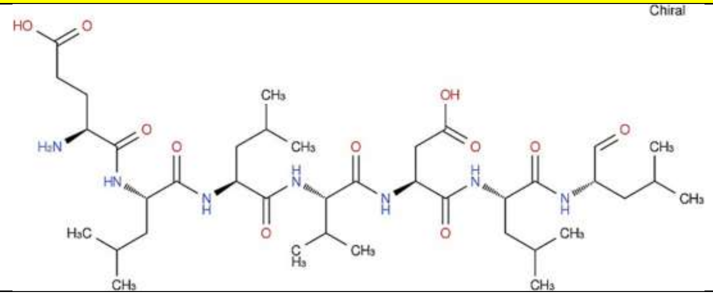
Cluster 2	Otherks	41244	Butirosin biosynthetic gene cluster (7% of genes show similarity)	-
Cluster 3	Transatpks	24266	Difficidin biosynthetic gene cluster (26% of genes show similarity)	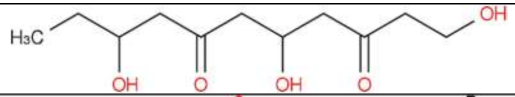
Cluster 4	Transatpks-Nrps	102695	Bacillaene biosynthetic gene cluster (100% of genes show similarity)	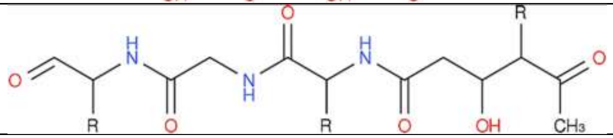
Cluster 5	Transatpks	85905	Macrolactin biosynthetic gene cluster (100% of genes show similarity)	-
Cluster 6	Terpene	20740	-	-
Cluster 7	Transatpks-Nrps	88755	Fengycin biosynthetic gene cluster (86% of genes show similarity)	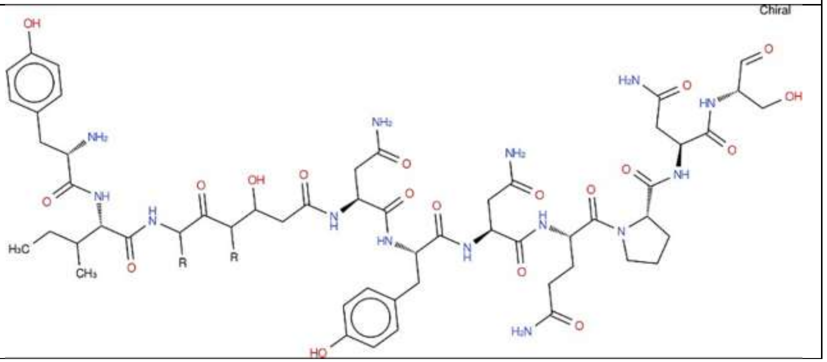

Table S1. Continued.

Strain clusters	Type	Length (bp)	Most similar known clusters	Predicted core clusters
**RC218**				
Cluster 8	Other	41418	Bacilysin biosynthetic gene cluster (100% of genes show similarity)	-
Cluster 9	Lantipeptide	27056	Subtilin biosynthetic gene cluster (100% of genes show similarity)	-
Cluster 10	Bacteriocin-Nrps	51792	Bacillibactin biosynthetic gene cluster (100% of genes show similarity)	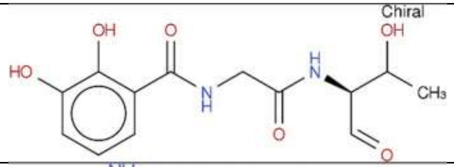
Cluster 11	Transatpks	28662	Difficidin biosynthetic gene cluster (46% of genes show similarity)	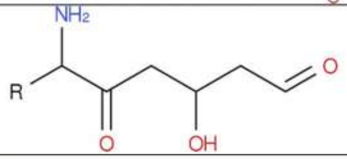
Cluster 12	Nrps	49224	Plipastatin biosynthetic gene cluster (53% of genes show similarity)	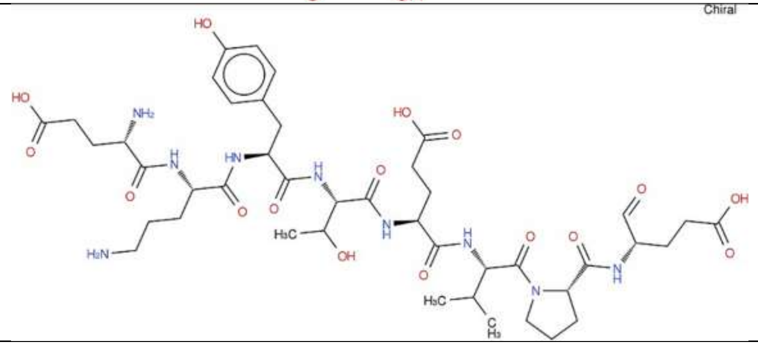
Cluster 13	Terpene	21883	-	-
Cluster 14	T3pks	41109	-	-
Cluster 15	Transatpks	46720	Difficidin biosynthetic gene cluster (53% of genes show similarity)	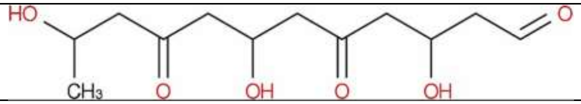

Table S1. Continued.

Strain clusters	Type	Length (bp)	Most similar known clusters	Predicted core clusters
**AP183**				
Cluster 1	Bacteriocin-Nrps	51792	Bacillibactin biosynthetic gene cluster (100% of genes show similarity)	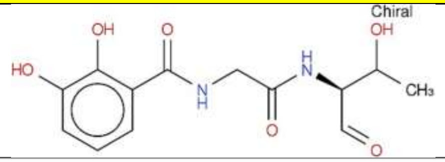
Cluster 2	Transatpks	75784	Difficidin biosynthetic gene cluster (86% of genes show similarity)	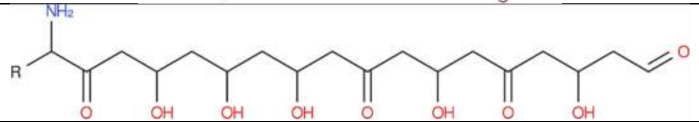
Cluster 3	Other	41418	Bacilysin biosynthetic gene cluster (100% of genes show similarity)	-
Cluster 4	Transatpks	46090	Myxovirescin biosynthetic gene cluster (17% of genes show similarity)	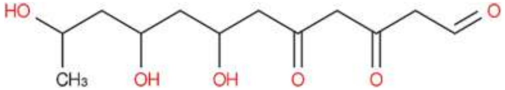
Cluster 5	Transatpks-T1pks	30008	Thiomarinol biosynthetic gene cluster (5% of genes show similarity)	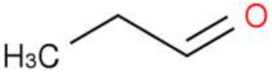
Cluster 6	Transatpks-T1pks	102692	Bacillaene biosynthetic gene cluster (100% of genes show similarity)	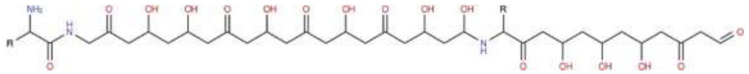
Cluster 7	Transatpks-T1pks	103075	Fengycin biosynthetic gene cluster (93% of genes show similarity)	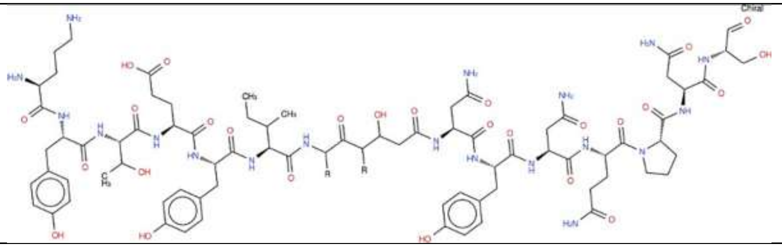
Cluster 8	Transatpks	68255	Macrolactin biosynthetic gene cluster (80% of genes show similarity)	-
Cluster 9	Terpene	20740	-	-
Cluster 10	Otherks	41244	Butirosin biosynthetic gene cluster (7% of genes show similarity)	-

Table S1. Continued.

Strain clusters	Type	Length (bp)	Most similar known clusters	Predicted core clusters
**AP183**				
Cluster 11	Nrps	25177	Surfactin biosynthetic gene cluster (43% of genes show similarity)	-
Cluster 12	Nrps	38360	Surfactin biosynthetic gene cluster (47% of genes show similarity)	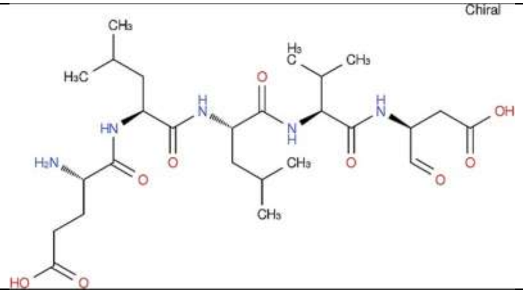
Cluster 13	Transatpks	47375	Difficidin biosynthetic gene cluster (26% of genes show similarity)	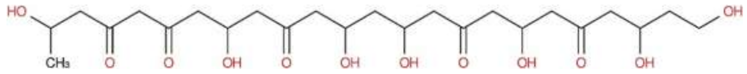
Cluster 14	Nrps	19928	Fengycin biosynthetic gene cluster (20% of genes show similarity)	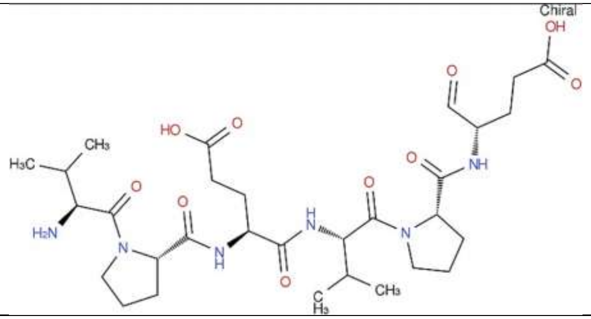
Cluster 15	Transatpks	8122	Bryostatin biosynthetic gene cluster (80% of genes show similarity)	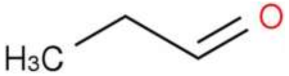
Cluster 16	T3pks	26386	-	-
Cluster 17	Terpene	21883	-	-

Table S1. Continued.

Strain clusters	Type	Length (bp)	Most similar known clusters	Predicted core clusters
**G341**				
Cluster 5	Nrps	65407	Surfactin biosynthetic gene cluster (91% of genes show similarity)	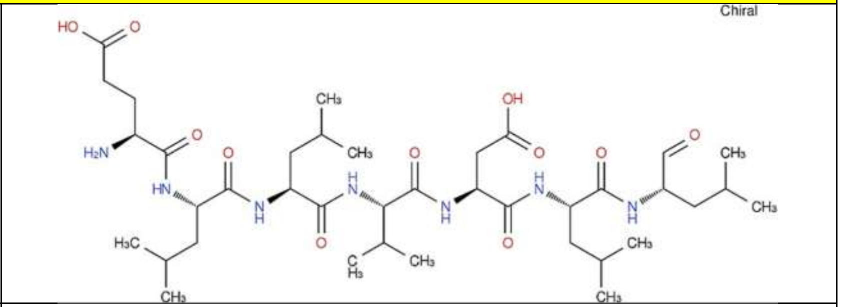
Cluster 2	Otherks	41244	Butirosin biosynthetic gene cluster (7% of genes show similarity)	-
Cluster 3	Lantipeptide-Terpene	45895	-	-
Cluster 4	Transatpks	85893	Macrolactin biosynthetic gene cluster (100% of genes show similarity)	-
Cluster 5	Nrps-Transatpks	102698	Bacillaene biosynthetic gene cluster (100% of genes show similarity)	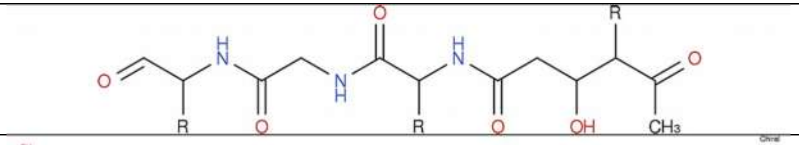
Cluster 6	Nrps-Transatpks	139131	Fengycin biosynthetic gene cluster (100% of genes show similarity)	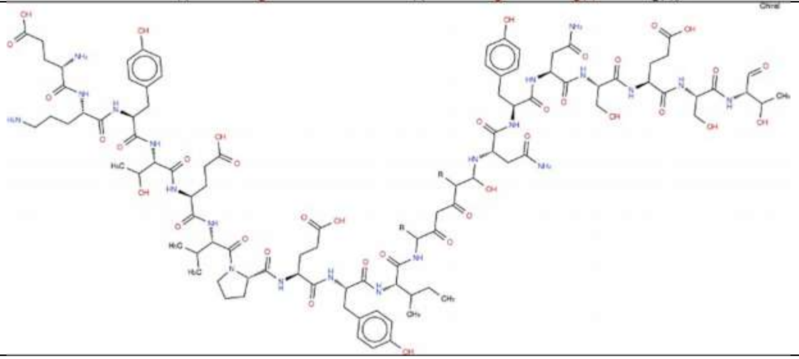
Cluster 7	Terpene	21883	-	-
Cluster 8	T3pks	41109	-	-

Table S1. Continued.

Strain clusters	Type	Length (bp)	Most similar known clusters	Predicted core clusters
**G341**				
Cluster 9	Transatpks	100450	Difficidin biosynthetic gene cluster (100% of genes show similarity)	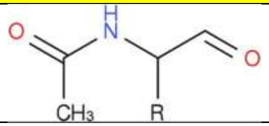
Cluster 10	Bacteriocin-Nrps	66794	Bacillibactin biosynthetic gene cluster (100% of genes show similarity)	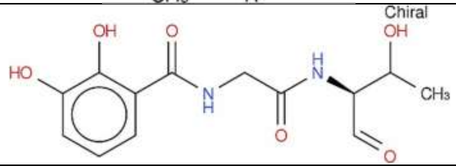
Cluster 11	Other	41418	Bacilysin biosynthetic gene cluster (100% of genes show similarity)	-

Table S1. Continued.

Strain clusters	Type	Length (bp)	Most similar known clusters	Predicted core clusters
**AP194**				
Cluster 1	Other	41418	Bacilysin biosynthetic gene cluster (100% of genes show similarity)	-
Cluster 2	Transatpks	85893	Macrolactin biosynthetic gene cluster (100% of genes show similarity)	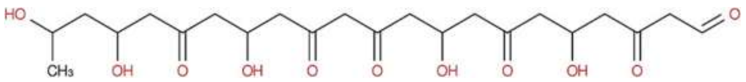
Cluster 3	Transatpks	45699	Difficidin biosynthetic gene cluster (53% of genes show similarity)	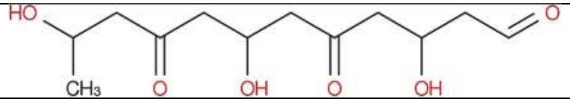
Cluster 4	Lantipeptide	27137	Haloduracin alpha / haloduracin beta biosynthetic gene cluster (40% of genes show similarity)	-
Cluster 5	Transatpks-Nrps	85704	Bacillaene biosynthetic gene cluster (100% of genes show similarity)	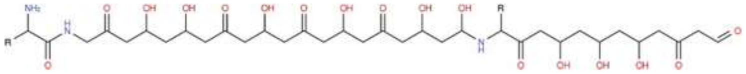
Cluster 6	T3pks	41100	-	-
Cluster 7	Nrps	23938	Plipastatin biosynthetic gene cluster (46% of genes show similarity)	-
Cluster 8	Terpene	21883	-	-
Cluster 9	Transatpks	30074	Difficidin biosynthetic gene cluster (46% of genes show similarity)	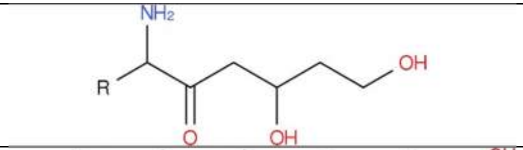
Cluster 10	Transatpks	24406	Difficidin biosynthetic gene cluster (26% of genes show similarity)	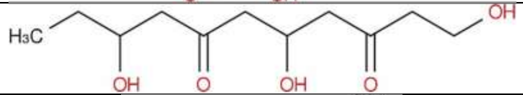
Cluster 11	Nrps	13196	Fengycin biosynthetic gene cluster (20% of genes show similarity)	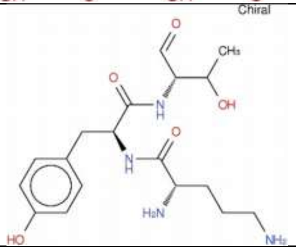

Table S1. Continued.

Strain clusters	Type	Length (bp)	Most similar known clusters	Predicted core clusters
**AP194**				
Cluster 12	Nrps	9648	Fengycin biosynthetic gene cluster (13% of genes show similarity)	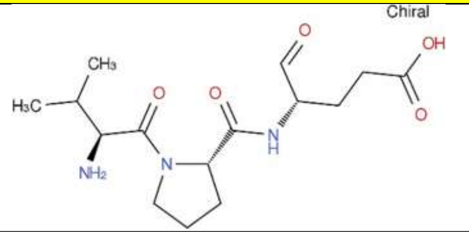
Cluster 13	Transatpks-Nrps	77730	Rhizocticin biosynthetic gene cluster (16% of genes show similarity)	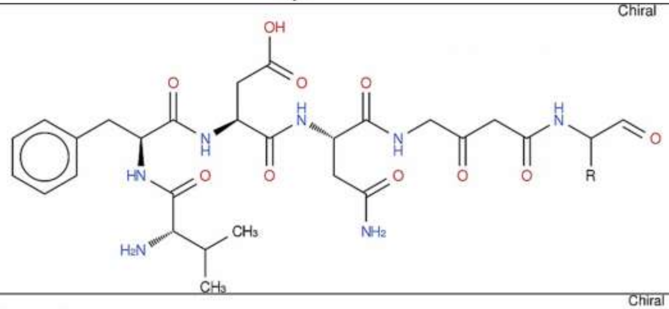
Cluster 14	Nrps	65413	Surfactin biosynthetic gene cluster (91% of genes show similarity)	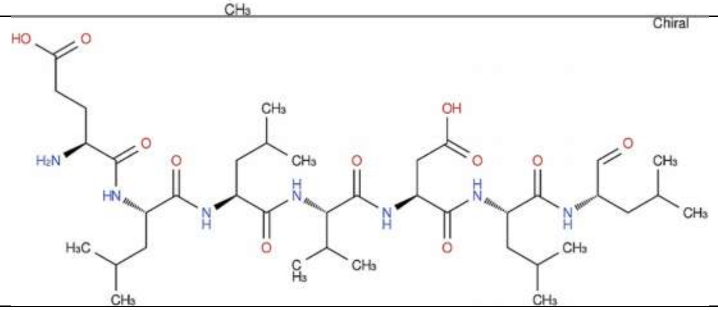
Cluster 15	Otherks	41244	Butirosin biosynthetic gene cluster (7% of genes show similarity)	-
Cluster 16	Terpene	20740	-	-
Cluster 17	Bacteriocin-Nrps	66789	Bacillibactin biosynthetic gene cluster (100% of genes show similarity)	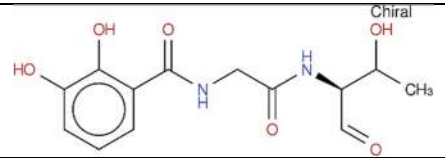

Table S1. Continued.

Strain clusters	Type	Length (bp)	Most similar known clusters	Predicted core clusters
**AP194**				
Cluster 18	Nrps-Transatpks	87890	Fengycin biosynthetic gene cluster (80% of genes show similarity)	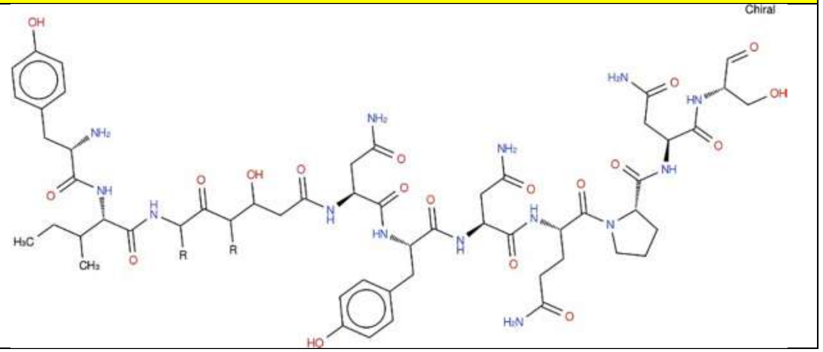

Table S1. Continued.

Strain clusters	Type	Length (bp)	Most similar known clusters	Predicted core clusters
**B25**				
Cluster 1	Nrps	65407	Surfactin biosynthetic gene cluster (78% of genes show similarity)	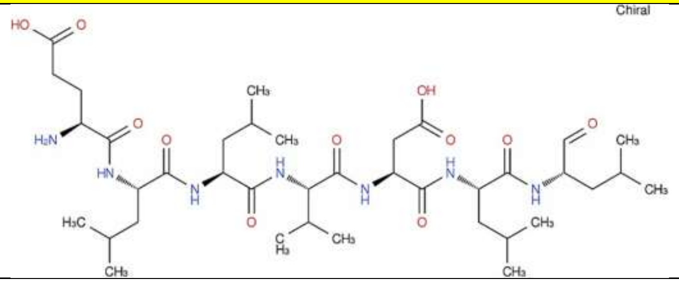
Cluster 2	Thiopeptide	45069	Kijanimicin biosynthetic gene cluster (4% of genes show similarity)	-
Cluster 3	Otherks	41244	Butirosin biosynthetic gene cluster (7% of genes show similarity)	-
Cluster 4	Terpene	20740	-	-
Cluster 5	Lantipeptide	28676	-	-
Cluster 6	Transatpks	85869	Macrolactin biosynthetic gene cluster (100% of genes show similarity)	-
Cluster 7	Transatpks-Nrps	102704	Bacillaene biosynthetic gene cluster (100% of genes show similarity)	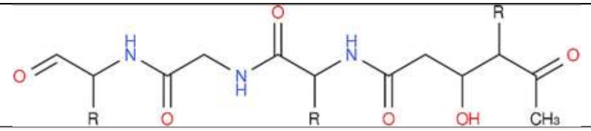
Cluster 8	Transatpks-Nrps	137827	Fengycin biosynthetic gene cluster (100% of genes show similarity)	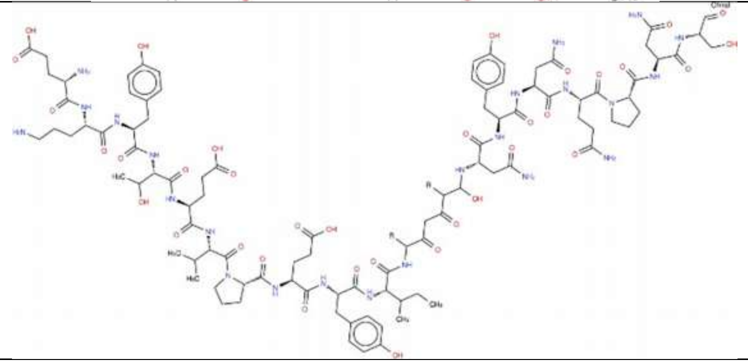

Table S1. Continued.

Strain clusters	Type	Length (bp)	Most similar known clusters	Predicted core clusters
**B25**				
Cluster 9	Terpene	21883	-	-
Cluster 10	T3pks	41109	-	-
Cluster 11	Transatpks	100438	Difficidin biosynthetic gene cluster (100% of genes show similarity)	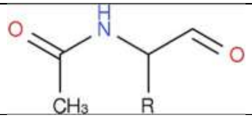
Cluster 12	Bacteriocin-Nrps	66792	Bacillibactin biosynthetic gene cluster (100% of genes show similarity)	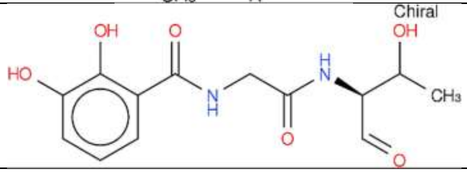
Cluster 13	Other	41418	Bacilysin biosynthetic gene cluster (100% of genes show similarity)	-

Table S1. Continued.

Strain clusters	Type	Length (bp)	Most similar known clusters	Predicted core clusters
**D2-2**				
Cluster 1	Nrps	65407	Surfactin biosynthetic gene cluster (82% of genes show similarity)	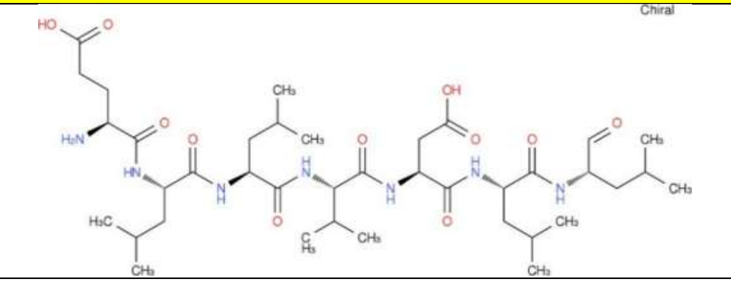
Cluster 2	Otherks	41244	Butirosin biosynthetic gene cluster (7% of genes show similarity)	-
Cluster 3	Terpene	20740	-	-
Cluster 4	Transatpks	85887	Macrolactin biosynthetic gene cluster (100% of genes show similarity)	-
Cluster 5	Transatpks-Nrps	102698	Bacillaene biosynthetic gene cluster (100% of genes show similarity)	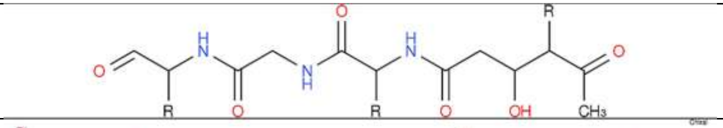
Cluster 6	Transatpks-Nrps	137962	Fengycin biosynthetic gene cluster (100% of genes show similarity)	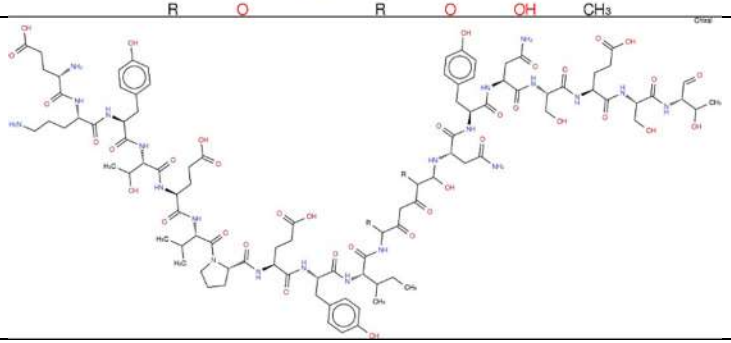
Cluster 7	Terpene	21883	-	-
Cluster 8	T3pks	41100	-	-
Cluster 9	Transatpks	98975	Difficidin biosynthetic gene cluster (100% of genes show similarity)	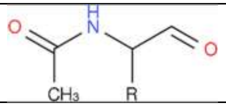

Table S1. Continued.

Strain clusters	Type	Length (bp)	Most similar known clusters	Predicted core clusters
**D2-2**				
Cluster 10	Bacteriocin-Nrps	66796	Bacillibactin biosynthetic genecluster (100% of genes show similarity)	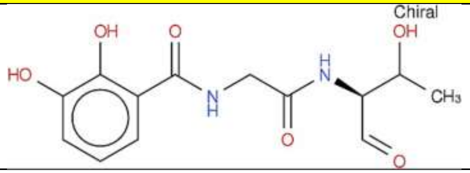
Cluster 11	Other	41418	Bacilysin biosynthetic gene cluster (100% of genes show similarity)	-
Cluster 12	Thiopeptide	29487	-	-

Table S1. Continued.

Strain clusters	Type	Length (bp)	Most similar known clusters	Predicted core clusters
**KACC_18228**				
Cluster 1	Transatpks-Nrps	88593	Fengycin biosynthetic gene cluster (86% of genes show similarity)	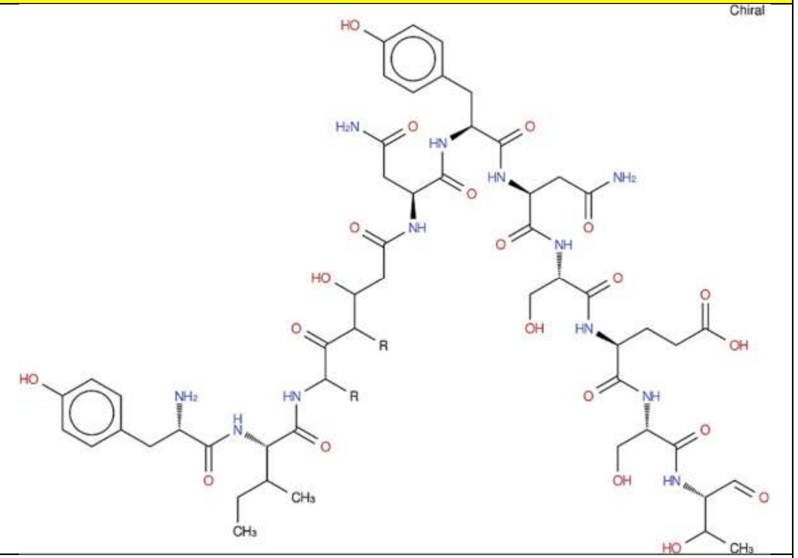
Cluster 2	Transatpks-Nrps	102686	Bacillaene biosynthetic gene cluster (100% of genes show similarity)	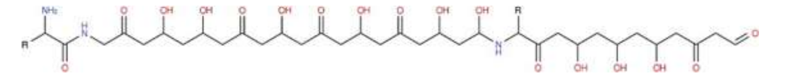
Cluster 3	Transatpks	85907	Macrolactin biosynthetic gene cluster (100% of genes show similarity)	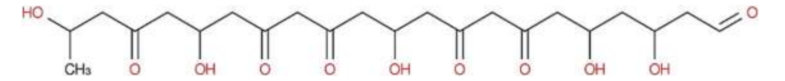
Cluster 4	Nrps	14235	Fengycin biosynthetic gene cluster (26% of genes show similarity)	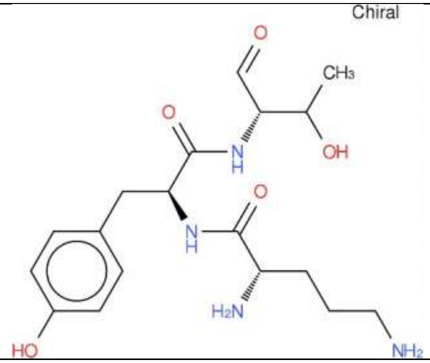

Table S1. Continued.

Strain clusters	Type	Length (bp)	Most similar known clusters	Predicted core clusters
**KACC_18228**				
Cluster 5	Bacteriocin-Nrps	66794	Bacillibactin biosynthetic gene cluster (100% of genes show similarity)	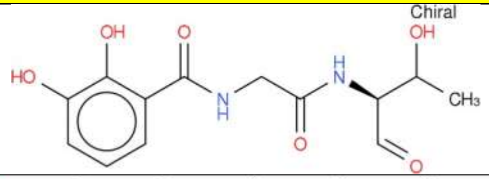
Cluster 6	Transatpks	46832	Difficidin biosynthetic gene cluster (53% of genes show similarity)	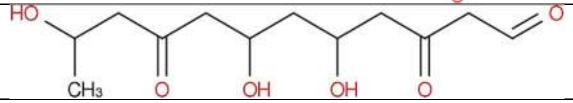
Cluster 7	Otherks	41244	Butirosin biosynthetic gene cluster (7% of genes show similarity)	-
Cluster 8	Terpene	20740	-	-
Cluster 9	Nrps	11190	Fengycin biosynthetic gene cluster (20% of genes show similarity)	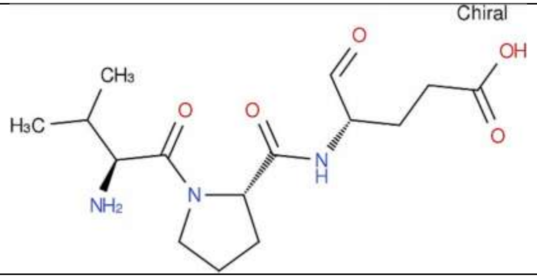
Cluster 10	Other	41418	Bacilysin biosynthetic gene cluster (100% of genes show similarity)	-
Cluster 11	Transatpks	53381	Difficidin biosynthetic gene cluster (60% of genes show similarity)	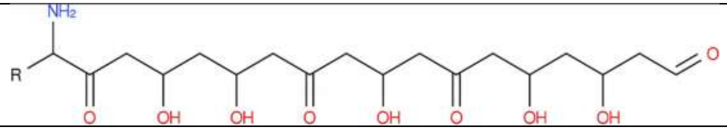
Cluster 12	T3pks	41109	-	-
Cluster 13	Nrps	22464	Plipastatin biosynthetic gene cluster (30% of genes show similarity)	-
Cluster 14	Thiopeptide-Lantipeptide	26819	-	-

Table S1. Continued.

Strain clusters	Type	Length (bp)	Most similar known clusters	Predicted core clusters
**KACC_18228**				
Cluster 15	Nrps	65410	Surfactin biosynthetic gene cluster (82% of genes show similarity)	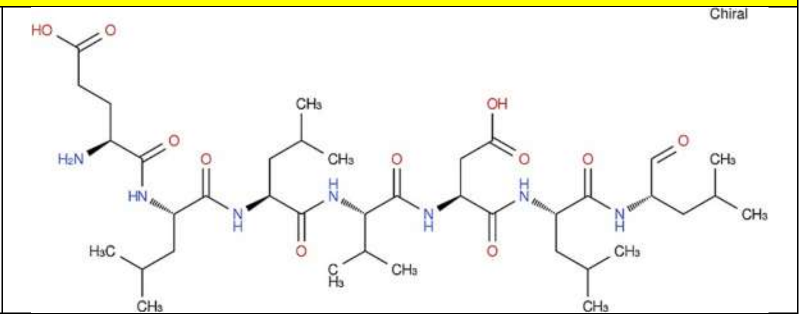

Table S1. Continued.

Strain clusters	Type	Length (bp)	Most similar known clusters	Predicted core clusters
**S3-1**				
Cluster 1	Nrps	65407	Surfactin biosynthetic gene cluster (82% of genes show similarity)	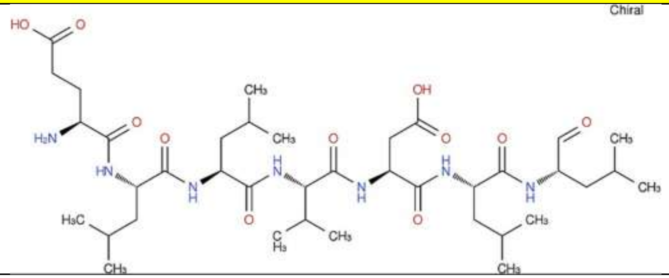
Cluster 2	Otherks	41244	Butirosin biosynthetic gene cluster (7% of genes show similarity)	-
Cluster 3	Terpene	20740	-	-
Cluster 4	Lantipeptide	28888	-	-
Cluster 5	Transatpks	85905	Macrolactin biosynthetic gene cluster (100% of genes show similarity)	-
Cluster 6	Transatpks-Nrps	102674	Bacillaene biosynthetic gene cluster (100% of genes show similarity)	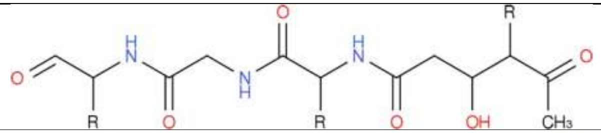
Cluster 7	Transatpks-Nrps	137801	Fengycin biosynthetic gene cluster (100% of genes show similarity)	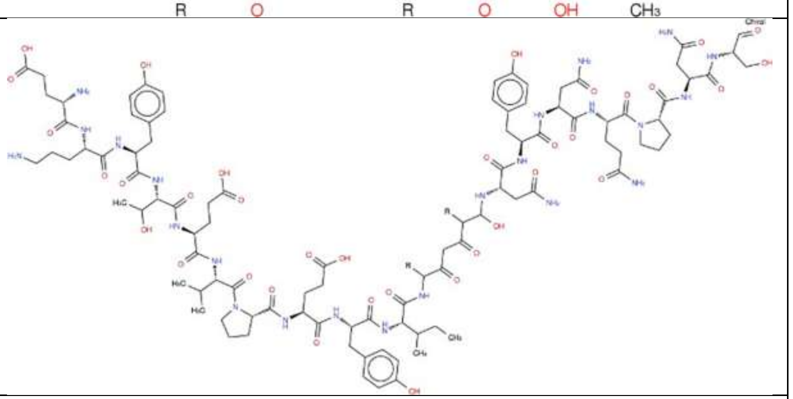
Cluster 8	Terpene	21883	-	-
Cluster 9	T3pks	41109	-	-

Table S1. Continued.

Strain clusters	Type	Length (bp)	Most similar known clusters	Predicted core clusters
**S3-1**				
Cluster 10	Transatpks	100453	Difficidin biosynthetic gene cluster (100% of genes show similarity)	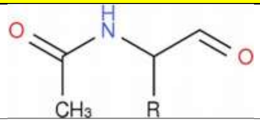
Cluster 18	Bacteriocin-Nrps	66791	Bacillibactin biosynthetic gene cluster (100% of genes show similarity)	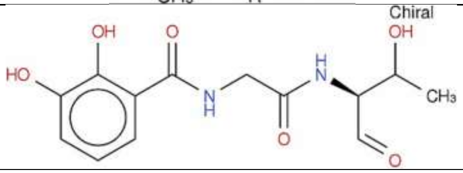
Cluster 19	Other	41418	Bacilysin biosynthetic gene cluster (100% of genes show similarity)	-

Table S1. Continued.

Strain clusters	Type	Length (bp)	Most similar known clusters	Predicted core clusters
**NBIF-003**				
Cluster 1	Nrps-Transatpks	114892	Fengycin biosynthetic gene cluster (93% of genes show similarity)	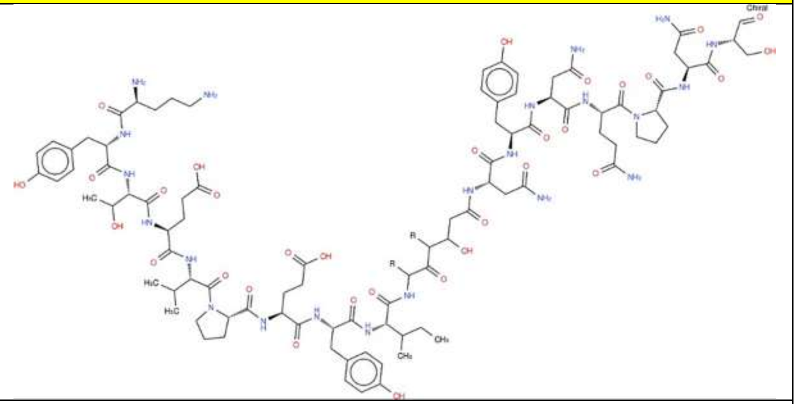
Cluster 2	Transatpks-Nrps	102674	Bacillaene biosynthetic gene cluster (100% of genes show similarity)	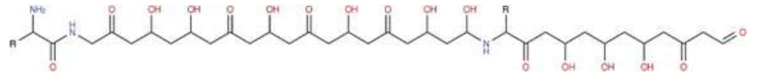
Cluster 3	Transatpks	85905	Macrolactin biosynthetic gene cluster (100% of genes show similarity)	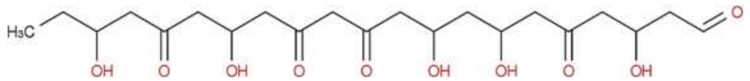
Cluster 4	Transatpks	100453	Difficidin biosynthetic gene cluster (100% of genes show similarity)	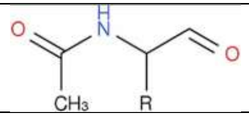
Cluster 5	T3pks	41109	-	-
Cluster 6	Terpene	21883	-	-
Cluster 7	Nrps	22957	Plipastatin biosynthetic gene cluster (30% of genes show similarity)	-
Cluster 8	Bacteriocin-Nrps	51791	Bacillibactin biosynthetic gene cluster (100% of genes show similarity)	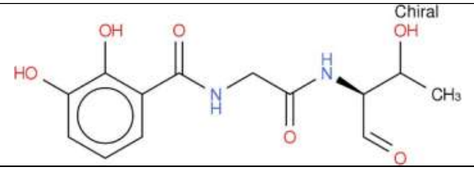
Cluster 9	Other	41418	Bacilysin biosynthetic gene cluster (100% of genes show similarity)	-

Table S1. Continued.

Strain clusters	Type	Length (bp)	Most similar known clusters	Predicted core clusters
**NBIF-003**				
Cluster 10	Nrps	65407	Surfactin biosynthetic gene cluster (82% of genes show similarity)	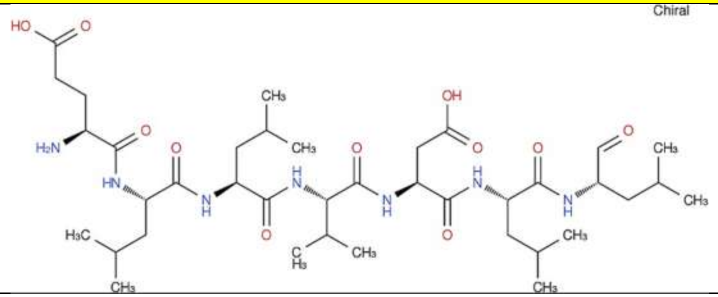
Cluster 11	Otherks	41244	Butirosin biosynthetic gene cluster (7% of genes show similarity)	-
Cluster 12	Terpene	20740	-	-
Cluster 13	Lantipeptide	28888	-	-

Table S1. Continued.

Strain clusters	Type	Length (bp)	Most similar known clusters	Predicted core clusters
**AP214**				
Cluster 1	Transatpks	53090	Difficidin biosynthetic gene cluster (60% of genes show similarity)	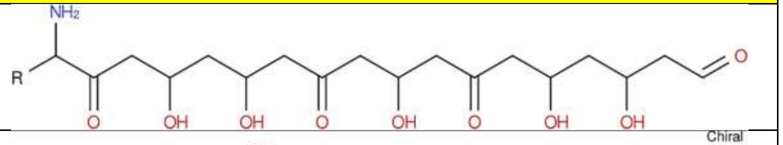
Cluster 2	Transatpks-Nrps	77735	Rhizocticin biosynthetic gene cluster (22% of genes show similarity)	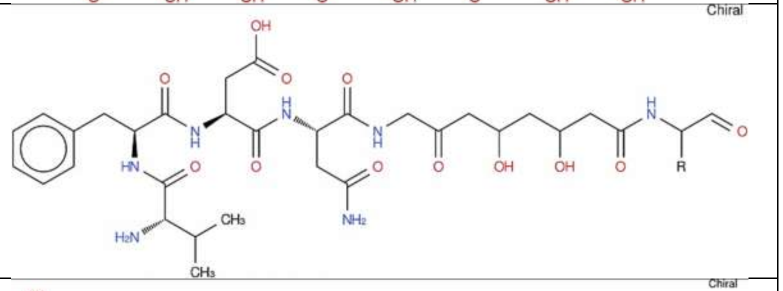
Cluster 3	Transatpks-Nrps	87728	Fengycin biosynthetic gene cluster (80% of genes show similarity)	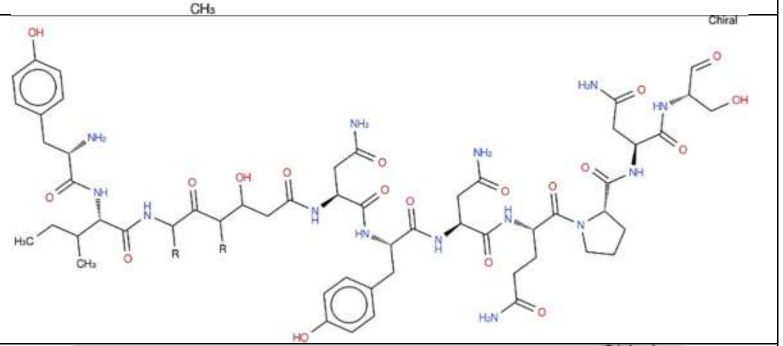
Cluster 4	Nrps	36551	Surfactin biosynthetic gene cluster (47% of genes show similarity)	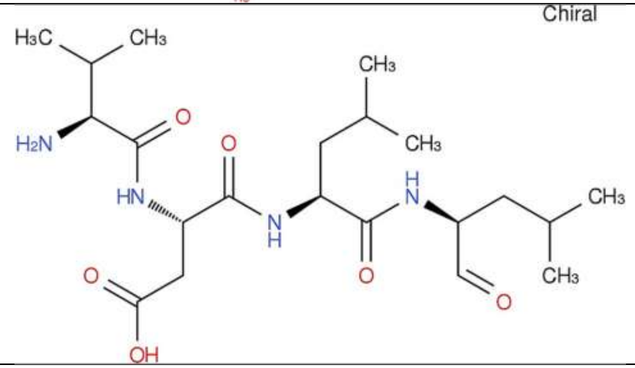

Table S1. Continued.

Strain clusters	Type	Length (bp)	Most similar known clusters	Predicted core clusters
**AP214**				
Cluster 5	Nrps	41963	Bacillibactin biosynthetic gene cluster (100% of genes show similarity)	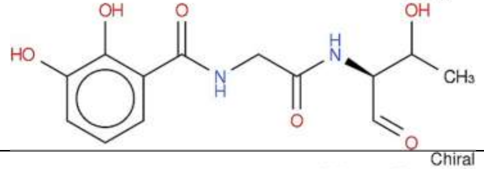
Cluster 6	Nrps	37288	Plipastatin biosynthetic gene cluster (46% of genes show similarity)	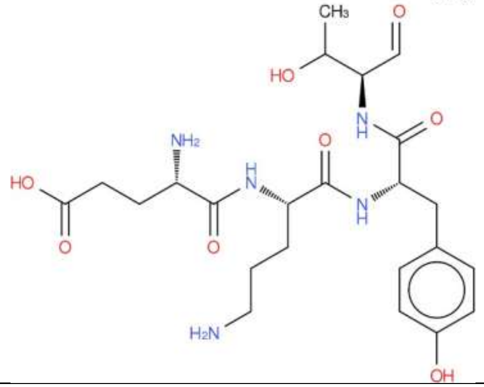
Cluster 7	Transatpks	45022	Difficidin biosynthetic gene cluster (46% of genes show similarity)	-
Cluster 8	T3pks	41109	-	-
Cluster 9	Terpene	21883	-	-
Cluster 10	Nrps	27924	Surfactin biosynthetic gene cluster (47% of genes show similarity)	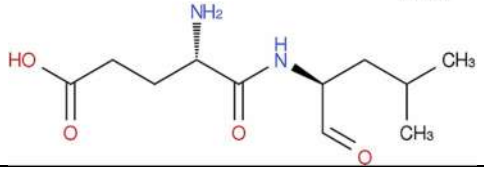
Cluster 11	Transatpks-Nrps	102683	Bacillaene biosynthetic gene cluster (100% of genes show similarity)	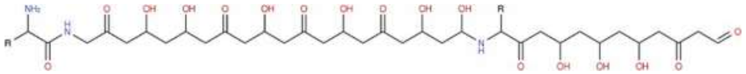

Table S1. Continued.

Strain clusters	Type	Length (bp)	Most similar known clusters	Predicted core clusters
**AP214**				
Cluster 12	Nrps	9651	Fengycin biosynthetic gene cluster (20% of genes show similarity)	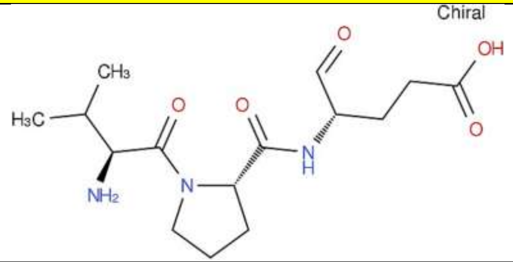
Cluster 13	Terpene	20740	-	-
Cluster 14	Otherks	41244	Butirosin biosynthetic gene cluster (7% of genes show similarity)	-
Cluster 15	Nrps	84176	Bacilysin biosynthetic gene cluster (100% of genes show similarity)	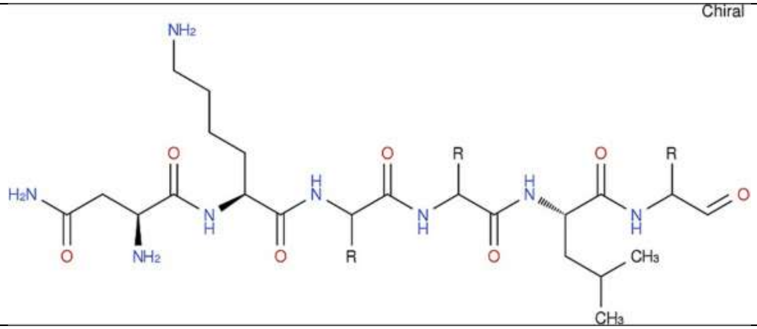
Cluster 16	Transatpks	85888	Macrolactin biosynthetic gene cluster (100% of genes show similarity)	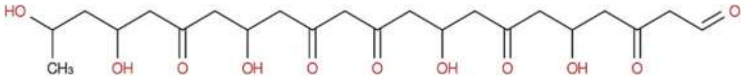

Table S1. Continued.

Strain clusters	Type	Length (bp)	Most similar known clusters	Predicted core clusters
**JS25R**				
Cluster 1	Nrps	65407	Surfactin biosynthetic gene cluster (82% of genes show similarity)	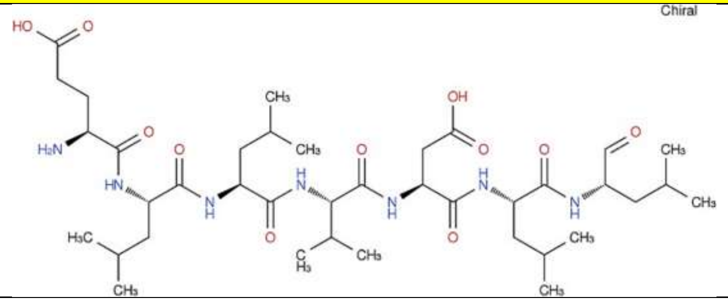
Cluster 2	Phosphonate	40902	-	-
Cluster 3	Otherks	41244	Butirosin biosynthetic gene cluster (7% of genes show similarity)	-
Cluster 4	Terpene	20740	-	-
Cluster 5	Transatpks	85899	Macrolactin biosynthetic gene cluster (100% of genes show similarity)	-
Cluster 6	Nrps-Transatpks	102683	Bacillaene biosynthetic gene cluster (100% of genes show similarity)	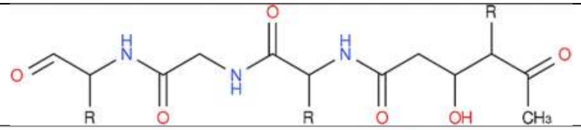
Cluster 7	Nrps-Transatpks	137830	Fengycin biosynthetic gene cluster (100% of genes show similarity)	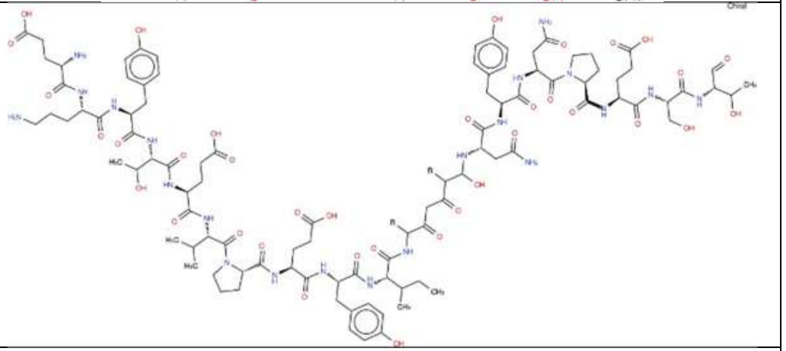
Cluster 8	Terpene	21883	-	-
Cluster 9	T3pks	41109	-	-

Table S1. Continued.

Strain clusters	Type	Length (bp)	Most similar known clusters	Predicted core clusters
**JS25R**				
Cluster 10	Transatpks	100444	Difficidin biosynthetic gene cluster (100% of genes show similarity)	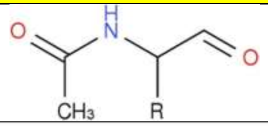
Cluster 11	Bacteriocin-Nrps	66795	Bacillibactin biosynthetic gene cluster (100% of genes show similarity)	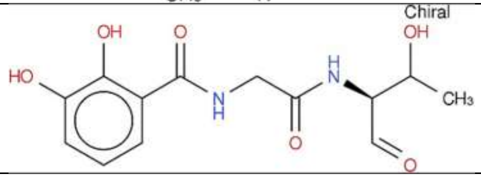
Cluster 12	Nrps	65342	-	-
Cluster 13	Other	41418	Bacilysin biosynthetic gene cluster (100% of genes show similarity)	-

Table S1. Continued.

Strain clusters	Type	Length (bp)	Most similar known clusters	Predicted core clusters
**UCMB5036**				
Cluster 1	Nrps	65407	Surfactin biosynthetic gene cluster (91% of genes show similarity)	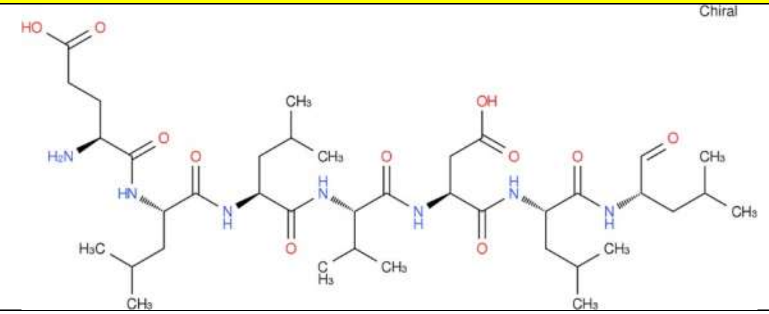
Cluster 2	Otherks	41244	Butirosin biosynthetic gene cluster (7% of genes show similarity)	-
Cluster 3	Terpene	20740	-	-
Cluster 4	Transatpks	85881	Macrolactin biosynthetic gene cluster (100% of genes show similarity)	-
Cluster 5	Transatpks-Nrps	102392	Bacillaene biosynthetic gene cluster (100% of genes show similarity)	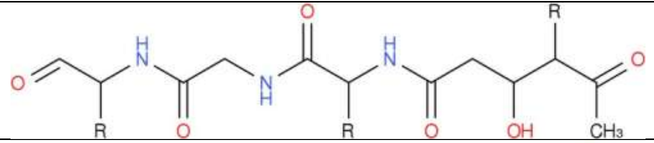
Cluster 6	Transatpks-Nrps	137815	Fengycin biosynthetic gene cluster (100% of genes show similarity)	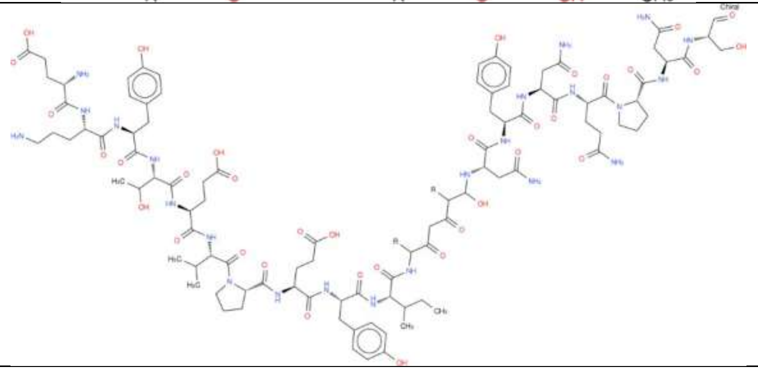
Cluster 7	Terpene	21883	-	-
Cluster 8	T3pks	41100	-	-

Table S1. Continued.

Strain clusters	Type	Length (bp)	Most similar known clusters	Predicted core clusters
**UCMB5036**				
Cluster 9	Transatpks	100447	Difficidin biosynthetic gene cluster (100% of genes show similarity)	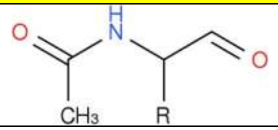
Cluster 10	Bacteriocin-Nrps	66791	Bacillibactin biosynthetic gene cluster (100% of genes show similarity)	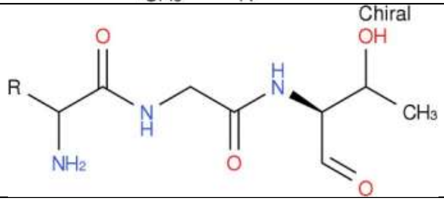
Cluster 11	Nrps	44115	-	-
Cluster 12	Other	41418	Bacilysin biosynthetic gene cluster (100% of genes show similarity)	-

Table S1. Continued.

Strain clusters	Type	Length (bp)	Most similar known clusters	Predicted core clusters
**SB1216**				
Cluster 1	Nrps	65407	Surfactin biosynthetic gene cluster (91% of genes show similarity)	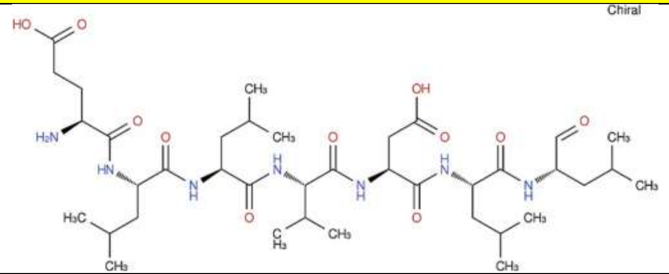
Cluster 2	Otherks	41244	Butirosin biosynthetic gene cluster (7% of genes show similarity)	-
Cluster 3	Terpene	20740	-	-
Cluster 4	Transatpks	83565	Macrolactin biosynthetic gene cluster (100% of genes show similarity)	-
Cluster 5	Nrps-Transatpks	102692	Bacillaene biosynthetic gene cluster (100% of genes show similarity)	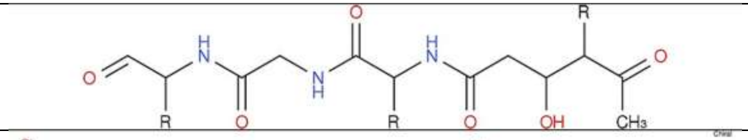
Cluster 6	Nrps-Transatpks	13172	Fengycin biosynthetic gene cluster (100% of genes show similarity)	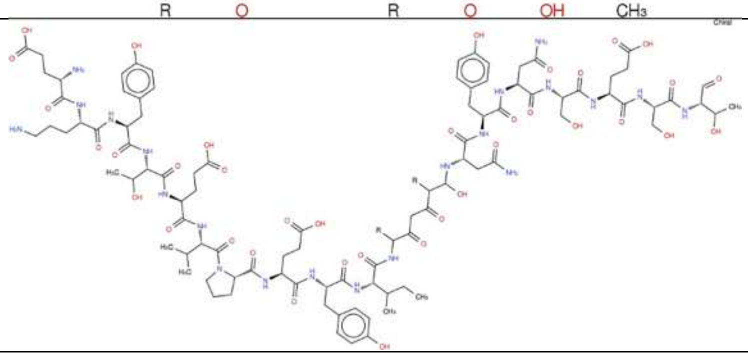
Cluster 7	Other	41418	Bacilysin_biosynthetic_gene_cluster (85% of genes show similarity)	-
Cluster 8	T3pks	41109	-	-

Table S1. Continued.

Strain clusters	Type	Length (bp)	Most similar known clusters	Predicted core clusters
**SB1216**				
Cluster 9	Transatpks	100456	Difficidin biosynthetic gene cluster (100% of genes show similarity)	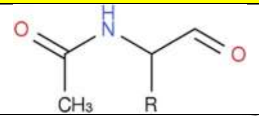
Cluster 10	Bacteriocin-Nrps	66792	Bacillibactin biosynthetic gene cluster (100% of genes show similarity)	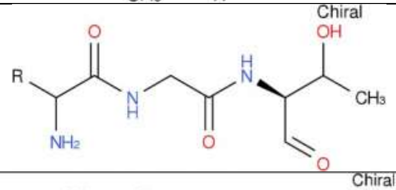
Cluster 11	Terpene	21883	Bacillibactin biosynthetic gene cluster (100% of genes show similarity)	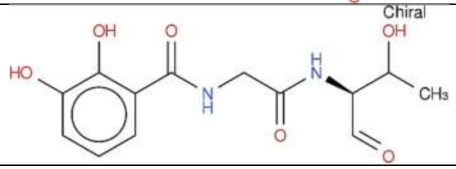

Table S1. Continued.

Strain clusters	Type	Length (bp)	Most similar known clusters	Predicted core clusters
**CC09**				
Cluster 1	Nrps-Transatpks-Bacteriocin	128301	Fengycin biosynthetic gene cluster (93% of genes show similarity)	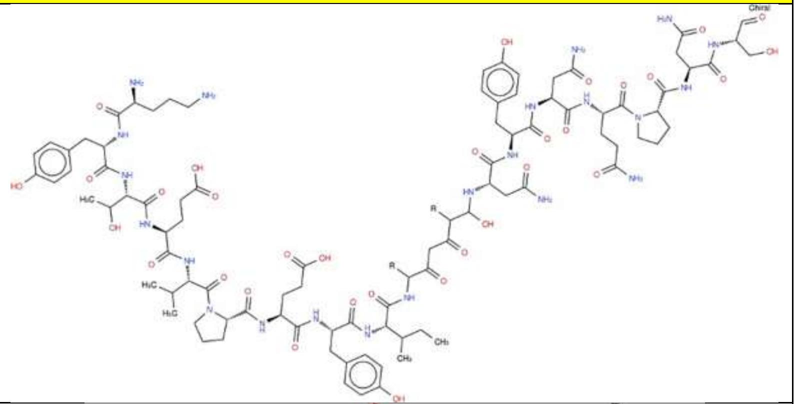
Cluster 2	Transatpks-Nrps	102683	Bacillaene biosynthetic gene cluster (100% of genes show similarity)	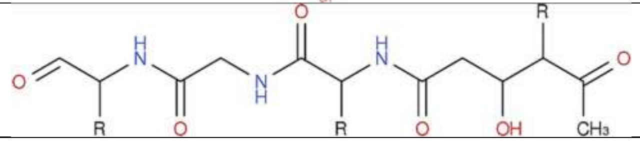
Cluster 3	Transatpks	85887	Macrolactin biosynthetic gene cluster (100% of genes show similarity)	-
Cluster 4	Terpene	20740	-	-
Cluster 5	Otherks	41244	Butirosin biosynthetic gene cluster (7% of genes show similarity)	-
Cluster 6	Nrps	65407	Surfactin biosynthetic gene cluster (91% of genes show similarity)	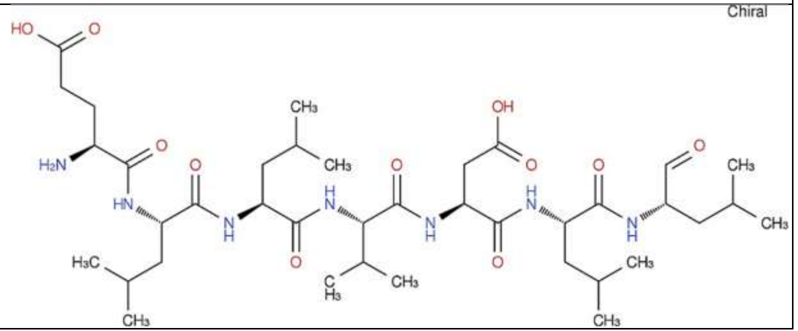

Table S1. Continued.

Strain clusters	Type	Length (bp)	Most similar known clusters	Predicted core clusters
**CC09**				
Cluster 7	Transatpks-Nrps	77757	Rhizocticin biosynthetic gene cluster (22% of genes show similarity)	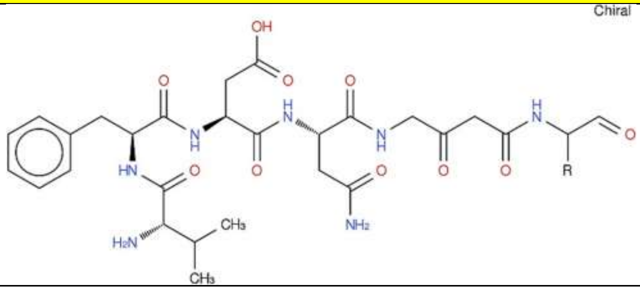
Cluster 8	Other	41418	Bacilysin biosynthetic gene cluster (85% of genes show similarity)	-
Cluster 9	Nrps	60005	-	-
Cluster 10	Bacteriocin-Nrps	51794	Bacillibactin biosynthetic gene cluster (100% of genes show similarity)	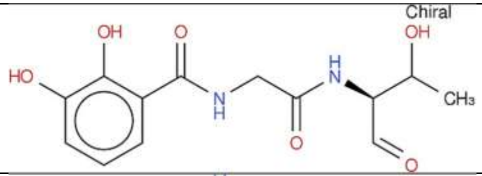
Cluster 11	Transatpks	100450	Difficidin biosynthetic gene cluster (100% of genes show similarity)	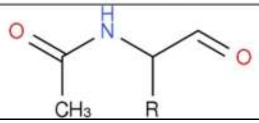
Cluster 12	T3pks	41109	-	-
Cluster 13	Terpene	21883	-	-
Cluster 14	Nrps	22720	Plipastatin biosynthetic gene cluster (30% of genes show similarity)	-

Table S1. Continued.

Strain clusters	Type	Length (bp)	Most similar known clusters	Predicted core clusters
**CFSAN034338**				
Cluster 1	Terpene	13634	-	-
Cluster 2	Terpene	29871	-	-
Cluster 3	Otherks	41244	Butirosin biosynthetic gene cluster (7% of genes show similarity)	-
Cluster 4	Transatpks	68716	Macrolactin biosynthetic gene cluster (100% of genes show similarity)	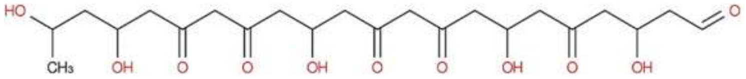
Cluster 5	Transatpks-Nrps	59131	Bacillaene biosynthetic gene cluster (85% of genes show similarity)	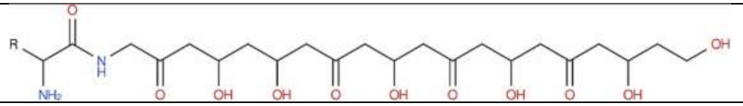
Cluster 6	Other	41418	Bacilysin biosynthetic gene cluster (100% of genes show similarity)	-
Cluster 7	Transatpks	28165	Difficidin biosynthetic gene cluster (46% of genes show similarity)	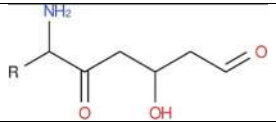
Cluster 8	Nrps	25179	Surfactin biosynthetic gene cluster (43% of genes show similarity)	-
Cluster 9	Bacteriocin-Nrps	26509	Bacillibactin biosynthetic gene cluster (76% of genes show similarity)	-
Cluster 10	T3pks	23877	-	-
Cluster 11	Nrps	27595	Surfactin biosynthetic gene cluster (47% of genes show similarity)	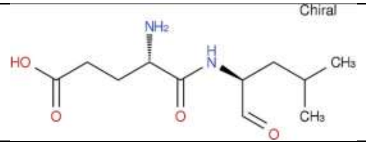
Cluster 12	Transatpks	22682	Difficidin biosynthetic gene cluster (26% of genes show similarity)	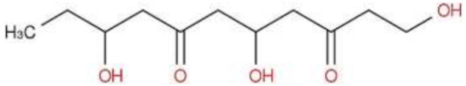
Cluster 13	Transatpks	39780	Bacillaene biosynthetic gene cluster (21% of genes show similarity)	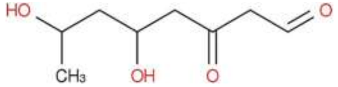

Table S1. Continued.

Strain clusters	Type	Length (bp)	Most similar known clusters	Predicted core clusters
**CFSAN034338**				
Cluster 14	Transatpks-Nrps	76026	Bacillomycin biosynthetic gene cluster (100% of genes show similarity)	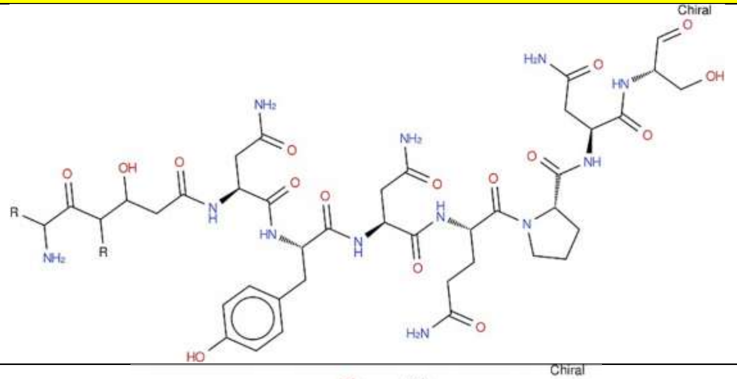
Cluster 15	Nrps	13938	Fengycin biosynthetic gene cluster (20% of genes show similarity)	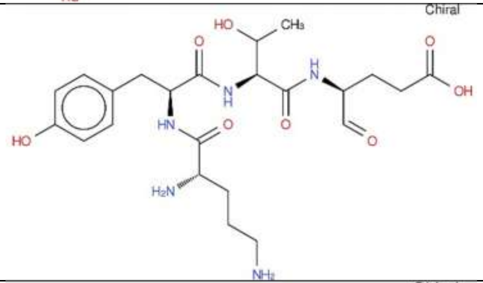
Cluster 16	Nrps	9325	Fengycin biosynthetic gene cluster (13% of genes show similarity)	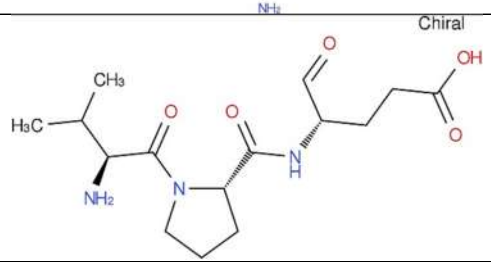
Cluster 17	Nrps	8901	Surfactin biosynthetic gene cluster (8% of genes show similarity)	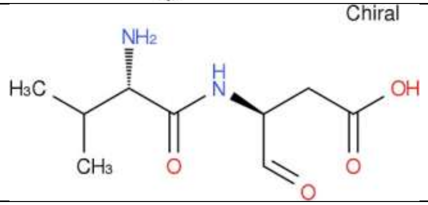

Table S1. Continued.

Strain clusters	Type	Length (bp)	Most similar known clusters	Predicted core clusters
**CFSAN034338**				
Cluster 18	Nrps	7875	Fengycin biosynthetic gene cluster (20% of genes show similarity)	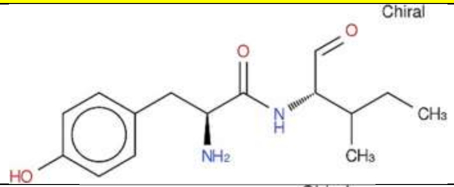
Cluster 19	Nrps	6339	Bacillibactin biosynthetic gene cluster (23% of genes show similarity)	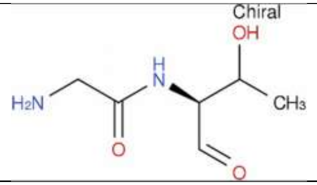
Cluster 20	Other	3125	-	-
Cluster 21	Nrps	1906	-	-
Cluster 22	Transatpks	45630	Difficidin biosynthetic gene cluster (53% of genes show similarity)	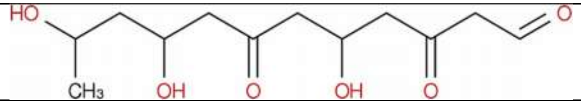

Table S1. Continued.

Strain clusters	Type	Length (bp)	Most similar known clusters	Predicted core clusters
**NRRL_B-4257**				
Cluster 1	Transatpks	100453	Difficidin biosynthetic gene cluster (100% of genes show similarity)	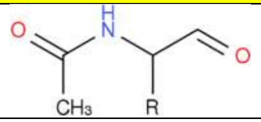
Cluster 2	T3pks	41109	-	-
Cluster 3	Terpene	20740	-	-
Cluster 4	Otherks	41244	Butirosin biosynthetic gene cluster (7% of genes show similarity)	-
Cluster 5	Terpene	21883	-	-
Cluster 6	Nrps	13862	Fengycin biosynthetic gene cluster (26% of genes show similarity)	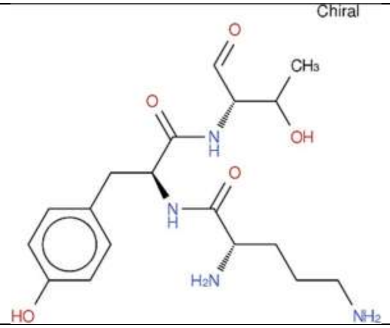
Cluster 7	Nrps	14408	Plipastatin biosynthetic gene cluster (30% of genes show similarity)	-
Cluster 8	Nrps	8922	Surfactin biosynthetic gene cluster (8% of genes show similarity)	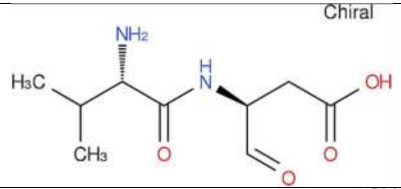
Cluster 9	Nrps	27617	Surfactin biosynthetic gene cluster (47% of genes show similarity)	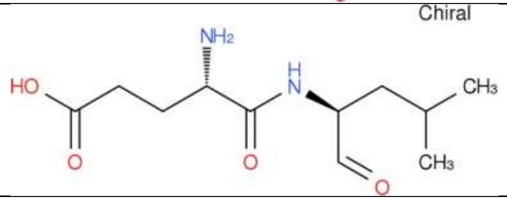

Table S1. Continued.

Strain clusters	Type	Length (bp)	Most similar known clusters	Predicted core clusters
**NRRL_B-4257**				
Cluster 10	Nrps	11967	Fengycin biosynthetic gene cluster (20% of genes show similarity)	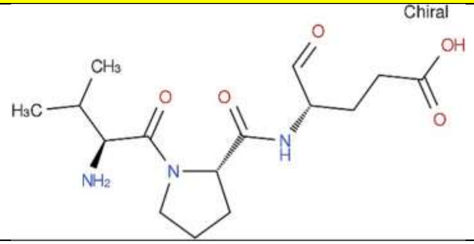
Cluster 11	Nrps	2335	-	-
Cluster 12	Nrps	1883	-	-
Cluster 13	Nrps	1223	-	-
Cluster 14	Phosphonate	40902	-	-
Cluster 15	Transatpks-Nrps	102677	Bacillaene biosynthetic gene cluster (100% of genes show similarity)	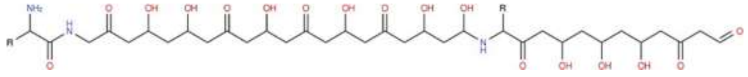
Cluster 16	Transatpks	85902	Macrolactin biosynthetic gene cluster (100% of genes show similarity)	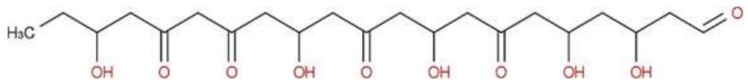
Cluster 17	Nrps	25178	Surfactin biosynthetic gene cluster (39% of genes show similarity)	-
Cluster 18	Nrps-Transatpks	88020	Fengycin biosynthetic gene cluster (80% of genes show similarity)	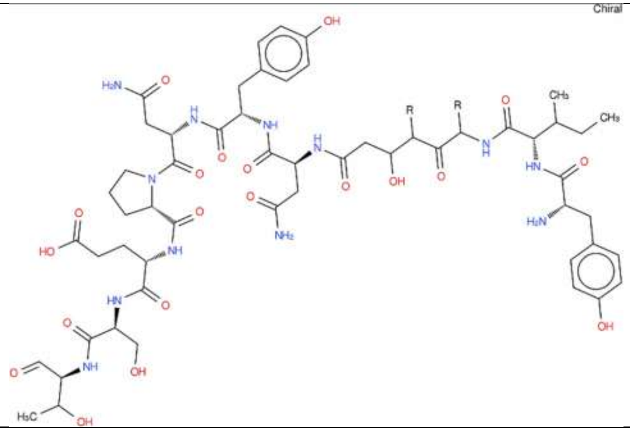

Table S1. Continued.

Strain clusters	Type	Length (bp)	Most similar known clusters	Predicted core clusters
**NRRL_B-4257**				
Cluster 20	Nrps	40295	-	-
Cluster 21	Other	41418	Bacilysin biosynthetic gene cluster (100% of genes show similarity)	-
Cluster 22	Lantipeptide	23984	Mersacidin biosynthetic gene cluster (100% of genes show similarity)	-
Cluster 23	Nrps-Bacteriocin	66793	Bacillibactin biosynthetic gene cluster (100% of genes show similarity)	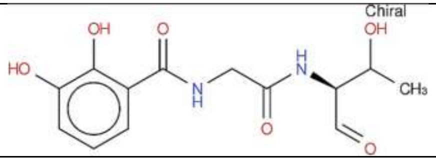

Table S1. Continued.

Strain clusters	Type	Length (bp)	Most similar known clusters	Predicted core clusters
**YAU_B9601-Y2**				
Cluster 1	Nrps	54640	Surfactin biosynthetic gene cluster (82% of genes show similarity)	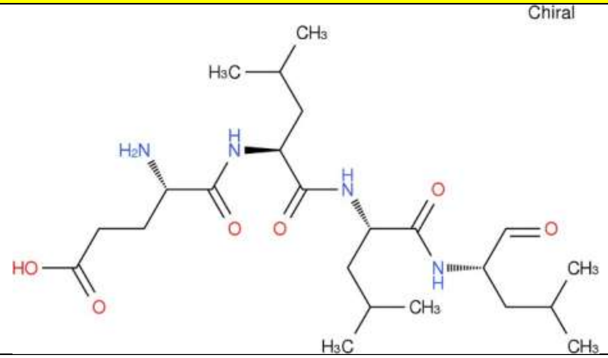
Cluster 2	Phosphonate	40884	-	-
Cluster 3	Otherks	41244	Butirosin biosynthetic gene cluster (7% of genes show similarity)	-
Cluster 4	Terpene	20740	-	-
Cluster 5	Transatpks	85902	Macrolactin biosynthetic gene cluster (100% of genes show similarity)	-
Cluster 6	Transatpks-Nrps	102680	Bacillaene biosynthetic gene cluster (100% of genes show similarity)	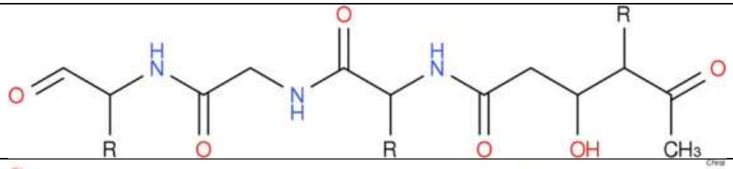
Cluster 7	Transatpks-Nrps	137831	Fengycin biosynthetic gene cluster (100% of genes show similarity)	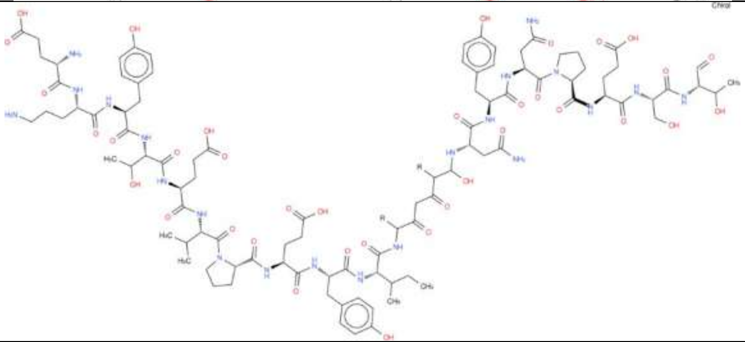

Table S1. Continued.

Strain clusters	Type	Length (bp)	Most similar known clusters	Predicted core clusters
**YAU_B9601-Y2**				
Cluster 8	Terpene	21883	-	-
Cluster 9	T3pks	41100	-	-
Cluster 10	Transatpks	100444	Difficidin biosynthetic gene cluster (100% of genes show similarity)	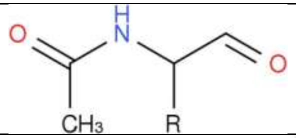
Cluster 11	Bacteriocin-Nrps	66796	Bacillibactin biosynthetic gene cluster (100% of genes show similarity)	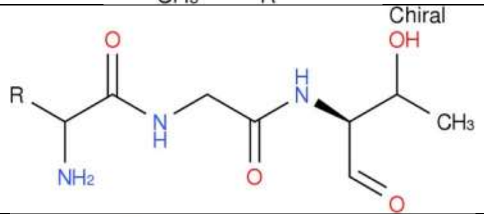
Cluster 12	Nrps	68420	-	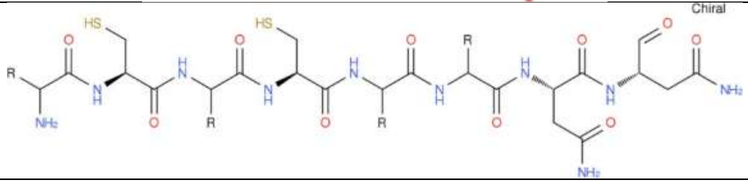
Cluster 13	Other	41400	Bacilysin biosynthetic gene cluster (100% of genes show similarity)	-
Cluster 14	Lantipeptide	23984	Mersacidin biosynthetic gene cluster (100% of genes show similarity)	-

Table S1. Continued.

Strain clusters	Type	Length (bp)	Most similar known clusters	Predicted core clusters
**OB9**				
Cluster 1	Nrps	59996	-	-
Cluster 2	Other	41418	Bacilysin biosynthetic gene cluster (100% of genes show similarity)	-
Cluster 3	Transatpks	29287	Difficidin biosynthetic gene cluster (46% of genes show similarity)	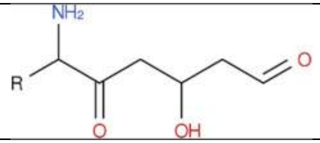
Cluster 4	Terpene	20740	-	-
Cluster 5	Transatpks	85896	Macrolactin biosynthetic gene cluster (100% of genes show similarity)	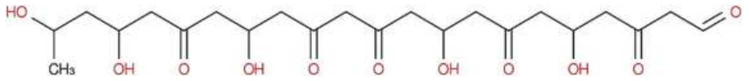
Cluster 6	Nrps-Transatpks	102689	Bacillaene biosynthetic gene cluster (100% of genes show similarity)	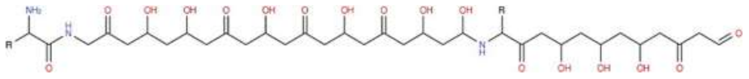
Cluster 7	Nrps-Transatpks	88306	Fengycin biosynthetic gene cluster (86% of genes show similarity)	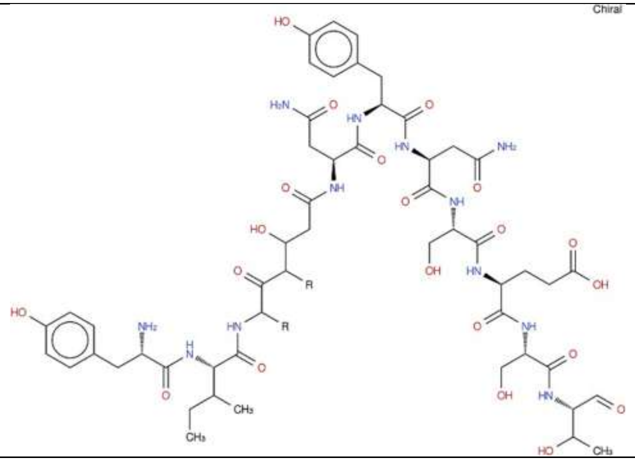
Cluster 8	Transatpks	46467	Difficidin biosynthetic gene cluster (53% of genes show similarity)	-
Cluster 9	T3pks	41109	-	-
Cluster 10	Terpene	21883	-	-

Table S1. Continued.

Strain clusters	Type	Length (bp)	Most similar known clusters	Predicted core clusters
**OB9**				
Cluster 11	Nrps	34385	Plipastatin biosynthetic gene cluster (46% of genes show similarity)	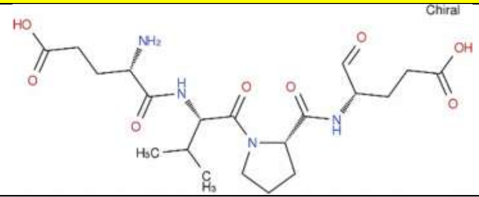
Cluster 12	Nrps	26341	Surfactin biosynthetic gene cluster (47% of genes show similarity)	-
Cluster 13	Nrps	39021	Surfactin biosynthetic gene cluster (52% of genes show similarity)	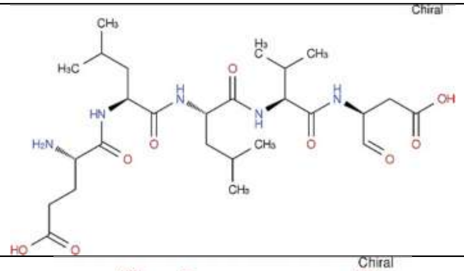
Cluster 14	Bacteriocin-Nrps	51794	Bacillibactin biosynthetic gene cluster (100% of genes show similarity)	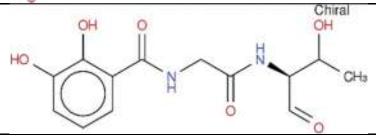
Cluster 15	Otherks	41244	Butirosin biosynthetic gene cluster (7% of genes show similarity)	-
Cluster 16	Transatpks	24960	Difficidin biosynthetic gene cluster (26% of genes show similarity)	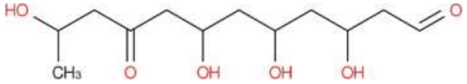
Cluster 17	Nrps	14478	Fengycin biosynthetic gene cluster (26% of genes show similarity)	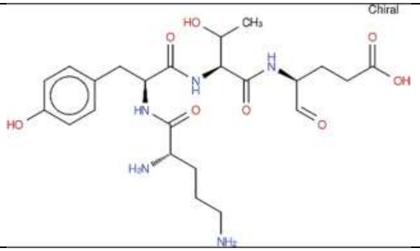

Table S1. Continued.

Strain clusters	Type	Length (bp)	Most similar known clusters	Predicted core clusters
**ZL918**				
Cluster 1	Nrps	65407	Surfactin biosynthetic gene cluster (78% of genes show similarity)	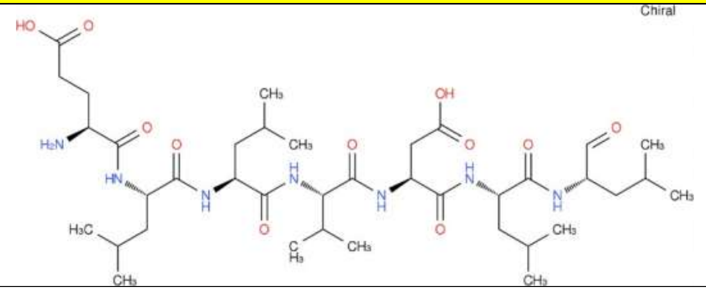
Cluster 2	Thiopeptide	27295	Kijanimicin biosynthetic gene cluster (4% of genes show similarity)	-
Cluster 3	Otherks	41244	Butirosin biosynthetic gene cluster (7% of genes show similarity)	-
Cluster 4	Terpene	20740	-	-
Cluster 5	Transatpks	85887	Macrolactin biosynthetic gene cluster (100% of genes show similarity)	-
Cluster 6	Transatpks-Nrps	102692	Bacillaene biosynthetic gene cluster (100% of genes show similarity)	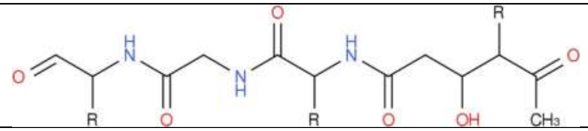
Cluster 7	Transatpks-Nrps	122420	Fengycin biosynthetic gene cluster (93% of genes show similarity)	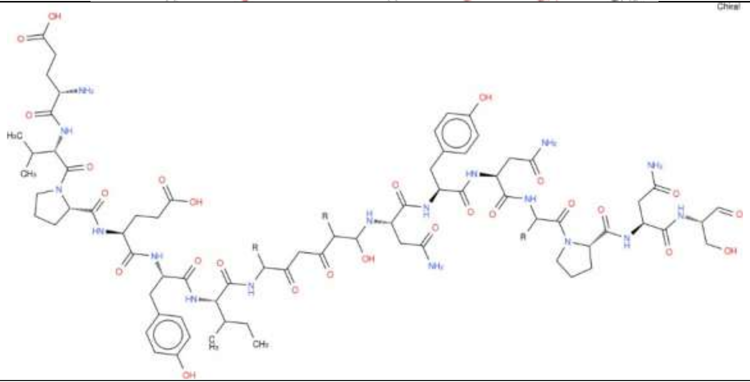

Table S1. Continued.

Strain clusters	Type	Length (bp)	Most similar known clusters	Predicted core clusters
**ZL918**				
Cluster 8	Terpene	21883	-	-
Cluster 9	T3pks	41100	-	-
Cluster 10	Transatpks	100450	Difficidin biosynthetic gene cluster (100% of genes show similarity)	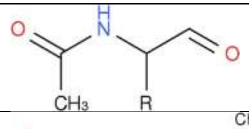
Cluster 11	Bacteriocin-Nrps	66792	Bacillibactin biosynthetic gene cluster (100% of genes show similarity)	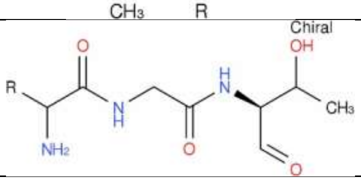
Cluster 12	Other	41418	Bacilysin biosynthetic gene cluster (100% of genes show similarity)	-

Table S1. Continued.

Strain clusters	Type	Length (bp)	Most similar known clusters	Predicted core clusters
**YJ11-1-4**				
Cluster 1	Other	41418	Bacilysin biosynthetic gene cluster (100% of genes show similarity)	-
Cluster 2	Bacteriocin-Nrps	66789	Bacillibactin biosynthetic gene cluster (100% of genes show similarity)	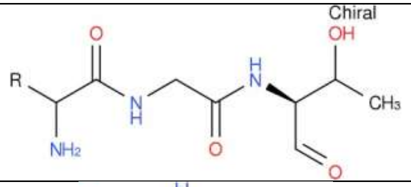
Cluster 3	Transatpks	100451	Difficidin biosynthetic gene cluster (100% of genes show similarity)	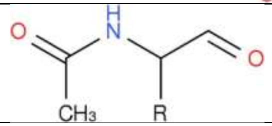
Cluster 4	T3pks	41100	-	-
Cluster 5	Terpene	21883	-	-
Cluster 6	Transatpks-Nrps	137810	Fengycin_biosynthetic_gene_cluster (100% of genes show similarity)	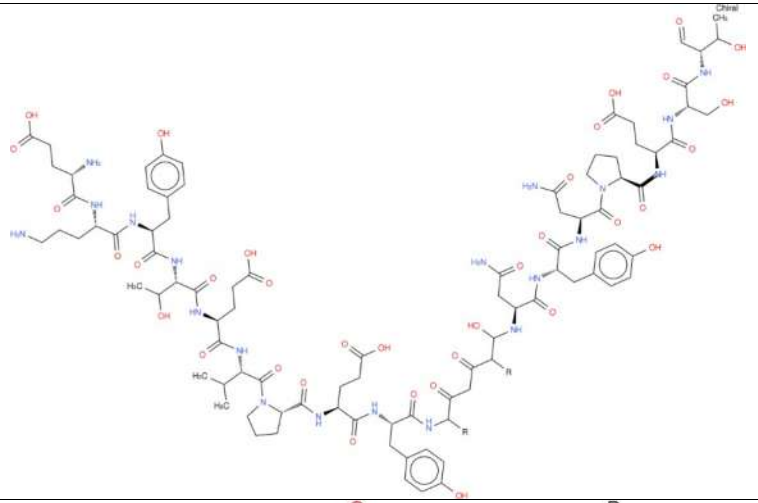
Cluster 7	Transatpks-Nrps	102670	Bacillaene biosynthetic gene cluster (100% of genes show similarity)	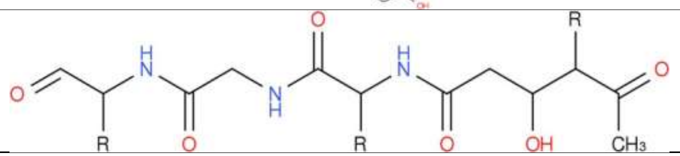

Table S1. Continued.

Strain clusters	Type	Length (bp)	Most similar known clusters	Predicted core clusters
**YJ11-1-4**				
Cluster 8	Transatpks	85889	Macrolactin biosynthetic gene cluster (100% of genes show similarity)	-
Cluster 9	Terpene	20740	-	-
Cluster 10	Otherks	41244	Butirosin biosynthetic gene cluster (7% of genes show similarity)	-
Cluster 11	Transatpks-T1pks	106587	Bacillaene biosynthetic gene cluster (64% of genes show similarity)	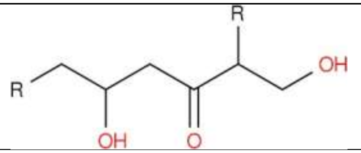
Cluster 12	Nrps	65406	Surfactin biosynthetic gene cluster (78% of genes show similarity)	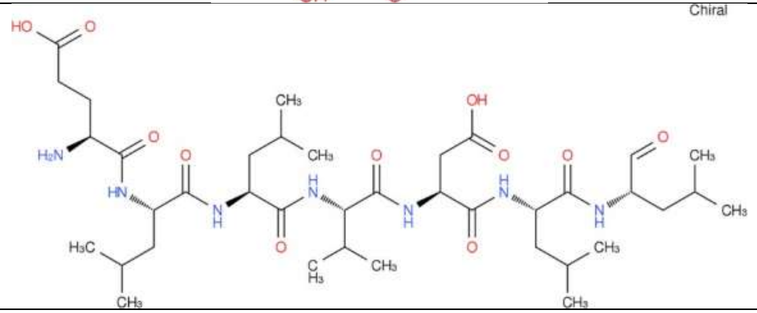

Table S1. Continued.

Strain clusters	Type	Length (bp)	Most similar known clusters	Predicted core clusters
**V4**				
Cluster 1	Other	41418	Bacilysin biosynthetic gene cluster (100% of genes show similarity)	-
Cluster 2	Nrps	25488	Surfactin biosynthetic gene cluster (39% of genes show similarity)	-
Cluster 3	Nrps	28039	Surfactin biosynthetic gene cluster (47% of genes show similarity)	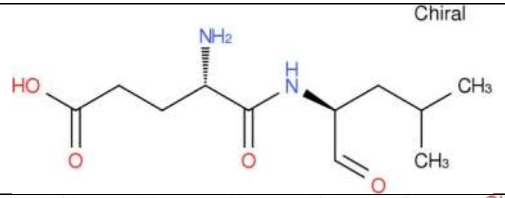
Cluster 4	Transatpks	23766	Difficidin biosynthetic gene cluster (26% of genes show similarity)	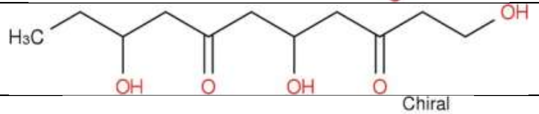
Cluster 5	Nrps	13267	Fengycin biosynthetic gene cluster (20% of genes show similarity)	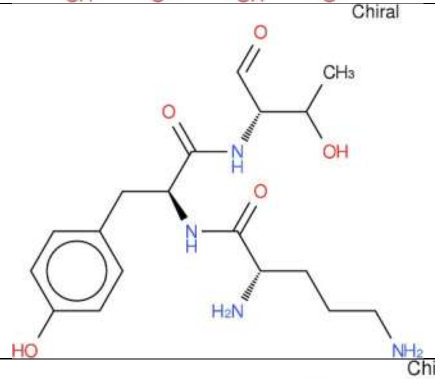
Cluster 6	Nrps	10261	Fengycin biosynthetic gene cluster (20% of genes show similarity)	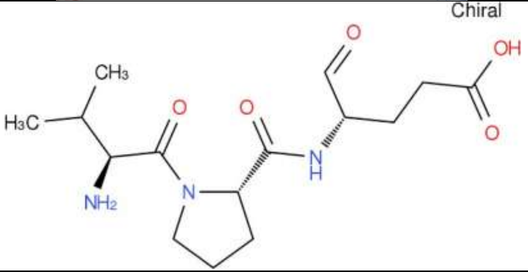

Table S1. Continued.

Strain clusters	Type	Length (bp)	Most similar known clusters	Predicted core clusters
**V4**				
Cluster 7	Bacteriocin-Nrps	66791	Bacillibactin biosynthetic gene cluster (100% of genes show similarity)	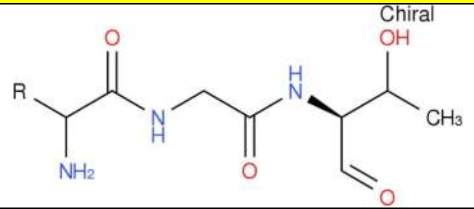
Cluster 8	Nrps	9654	Surfactin biosynthetic gene cluster (8% of genes show similarity)	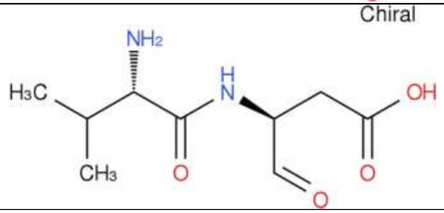
Cluster 9	Transatpks-Nrps	87854	Fengycin biosynthetic gene cluster (80% of genes show similarity)	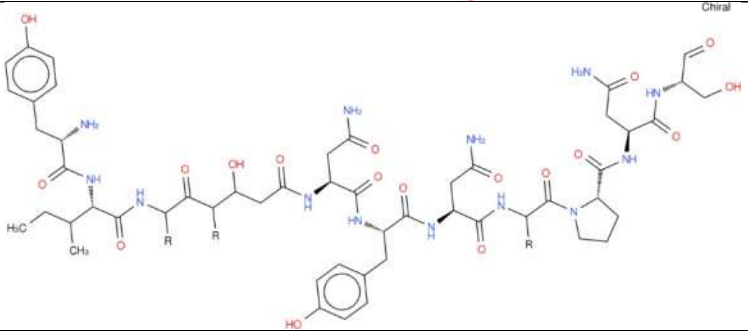
Cluster 10	Transatpks-Nrps	102674	Bacillaene biosynthetic gene cluster (100% of genes show similarity)	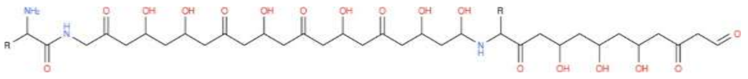
Cluster 11	Transatpks	85905	Macrolactin biosynthetic gene cluster (100% of genes show similarity)	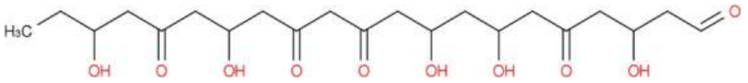
Cluster 12	Transatpks	28966	Difficidin biosynthetic gene cluster (46% of genes show similarity)	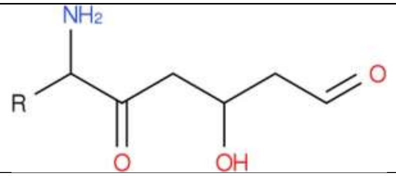

Table S1. Continued.

Strain clusters	Type	Length (bp)	Most similar known clusters	Predicted core clusters
**V4**				
Cluster 13	Otherks	41244	Butirosin biosynthetic gene cluster (7% of genes show similarity)	-
Cluster 14	Terpene	20740	-	-
Cluster 15	Lantipeptide	28888	-	-
Cluster 16	Transatpks	46296	Difficidin biosynthetic gene cluster (53% of genes show similarity)	-
Cluster 17	T3pks	41100	-	-
Cluster 18	Terpene	21883	-	-
Cluster 19	Nrps	21910	Plipastatin biosynthetic gene cluster (30% of genes show similarity)	-

Table S1. Continued.

Strain clusters	Type	Length (bp)	Most similar known clusters	Predicted core clusters
**W2**				
Cluster 1	Nrps	26386	-	-
Cluster 2	Bacteriocin-Nrps	66179	Bacillibactin biosynthetic gene cluster (100% of genes show similarity)	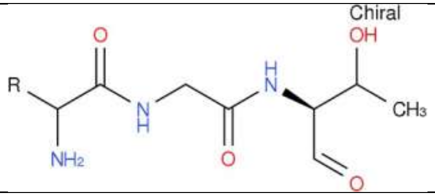
Cluster 3	Otherks	41244	Butirosin biosynthetic gene cluster (7% of genes show similarity)	-
Cluster 4	Terpene	20740	-	-
Cluster 5	Other	41418	Bacilysin biosynthetic gene cluster (100% of genes show similarity)	-
Cluster 6	Nrps	65408	Surfactin biosynthetic gene cluster (91% of genes show similarity)	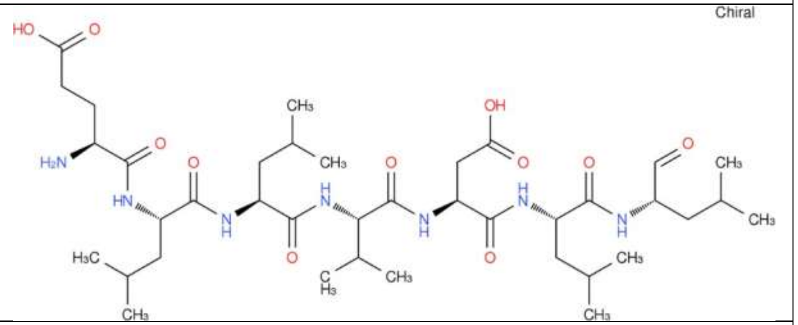
Cluster 7	Transatpks	85899	Macrolactin biosynthetic gene cluster (100% of genes show similarity)	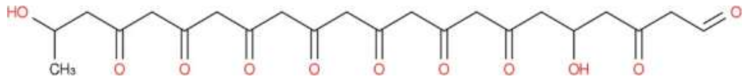
Cluster 8	Transatpks	100449	Difficidin biosynthetic gene cluster (100% of genes show similarity)	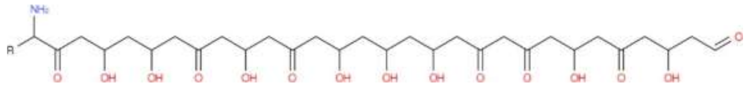
Cluster 9	T3pks	41100	-	-
Cluster 10	Terpene	21883	-	-

Table S1. Continued.

Strain clusters	Type	Length (bp)	Most similar known clusters	Predicted core clusters
**W2**				
Cluster 11	Bacteriocin-Transatpks-Nrps	150962	Fengycin biosynthetic gene cluster (100% of genes show similarity)	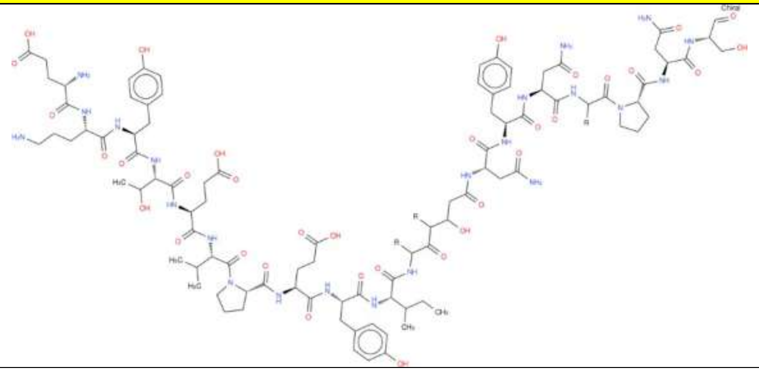
Cluster 12	Transatpks-Nrps	102680	Bacillaene biosynthetic gene cluster (100% of genes show similarity)	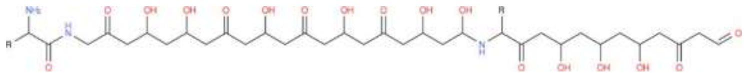
Cluster 13	Other	22150	-	-
Cluster 14	Nrps	8736	-	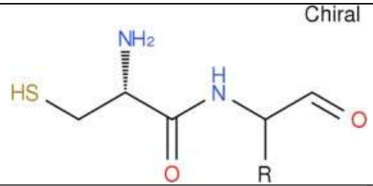
Cluster 15	Nrps	7328	-	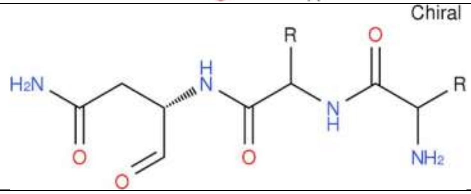
Cluster 16	Nrps	2543	-	-
Cluster 17	Other	1086	-	-

Table S1. Continued.

Strain clusters	Type	Length (bp)	Most similar known clusters	Predicted core clusters
**UCMB5113**				
Cluster 1	Lantipeptide	22615	Locillomycin biosynthetic gene cluster (35% of genes show similarity)	-
Cluster 2	Nrps	65407	Surfactin biosynthetic gene cluster (91% of genes show similarity)	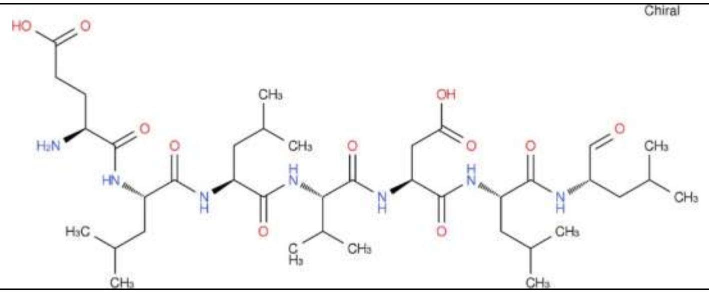
Cluster 3	Ladderane	41121	-	-
Cluster 4	Otherks	41244	Butirosin biosynthetic gene cluster (7% of genes show similarity)	-
Cluster 5	Terpene	20740	-	-
Cluster 6	Transatpks	85899	Macrolactin biosynthetic gene cluster (100% of genes show similarity)	-
Cluster 7	Transatpks-Nrps	102698	Bacillaene biosynthetic gene cluster (100% of genes show similarity)	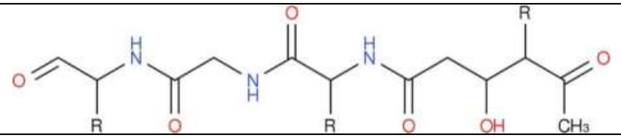
Cluster 8	Transatpks-Nrps	137848	Fengycin biosynthetic gene cluster (100% of genes show similarity)	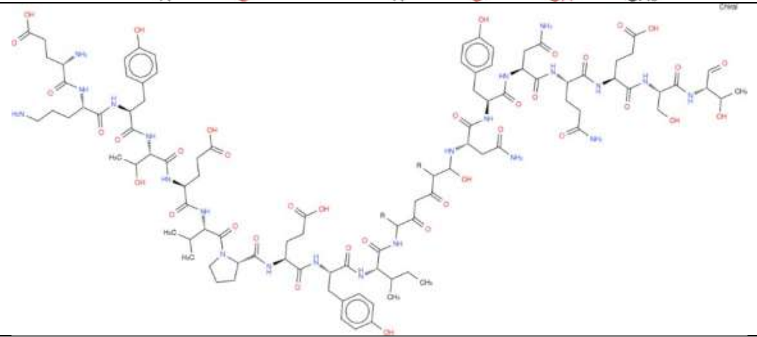

Table S1. Continued.

Strain clusters	Type	Length (bp)	Most similar known clusters	Predicted core clusters
**UCMB5113**				
Cluster 9	Terpene	21883	-	-
Cluster 10	T3pks	41100	-	-
Cluster 11	Transatpks	100453	Difficidin biosynthetic gene cluster (100% of genes show similarity)	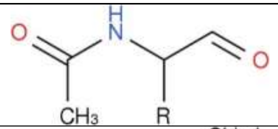
Cluster 12	Nrps	52066	-	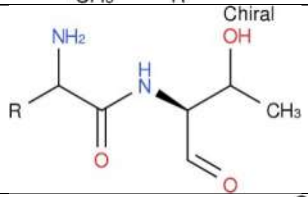
Cluster 13	Bacteriocin-Nrps	66793	Bacillibactin biosynthetic gene cluster (100% of genes show similarity)	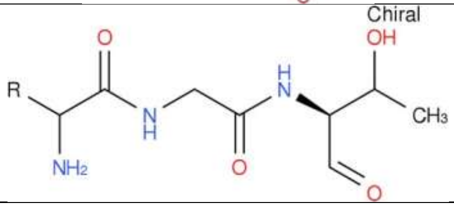
Cluster 14	Other	41418	Bacilysin biosynthetic gene cluster (100% of genes show similarity)	-

Table S1. Continued.

Strain clusters	Type	Length (bp)	Most similar known clusters	Predicted core clusters
**UCMB5033**				
Cluster 1	Transatpks-Nrps	69228	Locillomycin biosynthetic gene cluster (100% of genes show similarity)	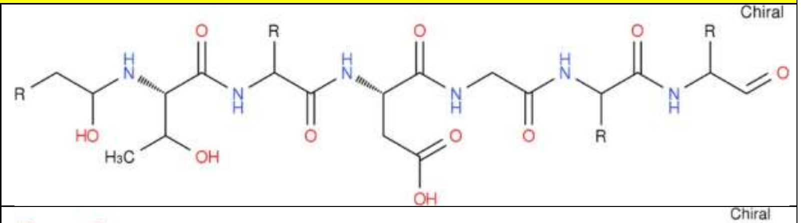
Cluster 2	Nrps	65407	Surfactin biosynthetic gene cluster (91% of genes show similarity)	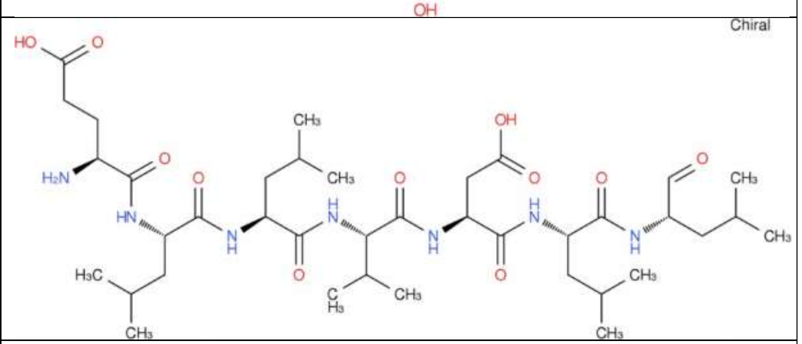
Cluster 3	Otherks	41244	Butirosin biosynthetic gene cluster (7% of genes show similarity)	-
Cluster 4	Terpene	20740	-	-
Cluster 5	Transatpks	85896	Macrolactin biosynthetic gene cluster (100% of genes show similarity)	-
Cluster 6	Transatpks-Nrps	102695	Bacillaene biosynthetic gene cluster (100% of genes show similarity)	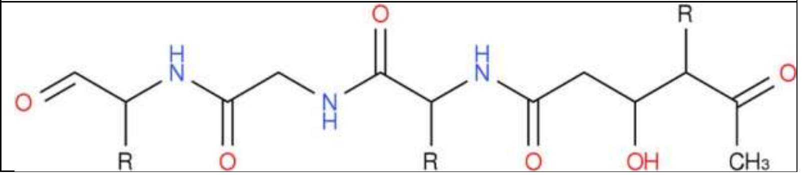

Table S1. Continued.

Strain clusters	Type	Length (bp)	Most similar known clusters	Predicted core clusters
**UCMB5033**				
Cluster 7	Transatpks-Nrps	122436	Fengycin biosynthetic gene cluster (93% of genes show similarity)	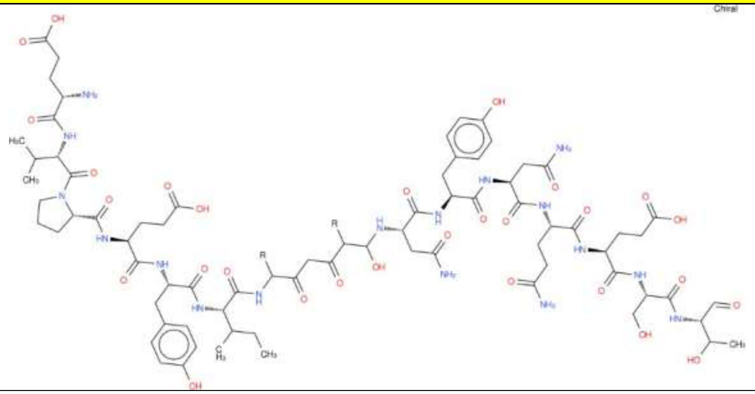
Cluster 8	Terpene	21883	-	-
Cluster 9	T3pks	41100	-	-
Cluster 10	Transatpks	100450	Difficidin biosynthetic gene cluster (100% of genes show similarity)	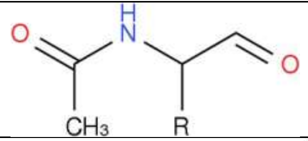
Cluster 11	Bacteriocin-Nrps	66792	Bacillibactin biosynthetic gene cluster (100% of genes show similarity)	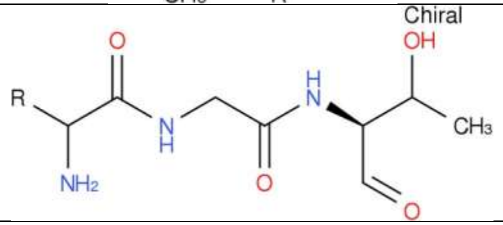
Cluster 12	Other	41418	Bacilysin biosynthetic gene cluster (100% of genes show similarity)	-

Table S1. Continued.

Strain clusters	Type	Length (bp)	Most similar known clusters	Predicted core clusters
**SYBC_H47**				
Cluster 1	Transatpks	75781	Difficidin biosynthetic gene cluster (86% of genes show similarity)	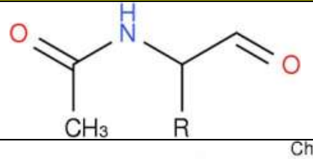
Cluster 2	T3pks-Nrps	87478	Plipastatin biosynthetic gene cluster (23% of genes show similarity)	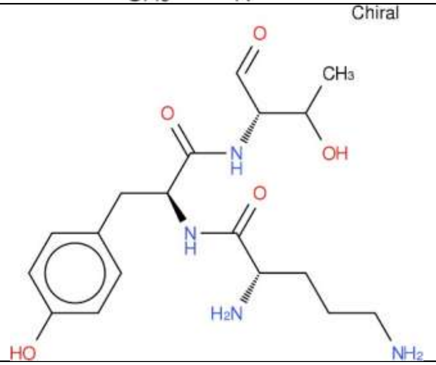
Cluster 3	Terpene	21883	-	-
Cluster 4	Transatpks-Nrps	111281	Fengycin biosynthetic gene cluster (93% of genes show similarity)	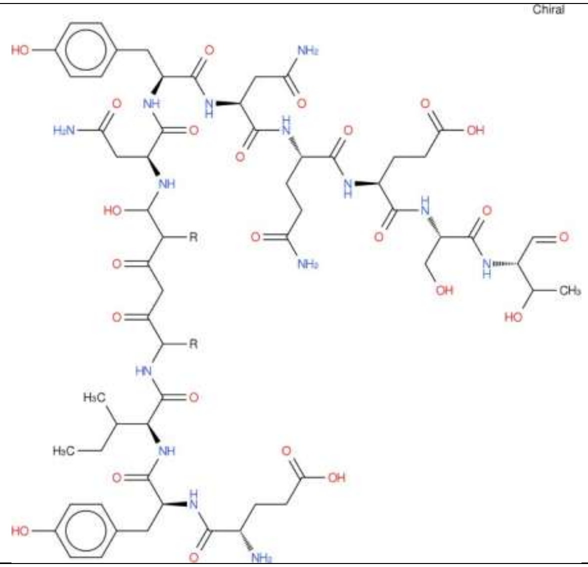

Table S1. Continued.

Strain clusters	Type	Length (bp)	Most similar known clusters	Predicted core clusters
**SYBC_H47**				
Cluster 5	Transatpks-Nrps	102701	Bacillaene biosynthetic gene cluster (100% of genes show similarity)	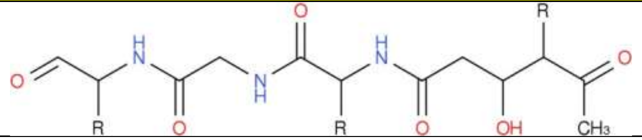
Cluster 6	Transatpks	85877	Macrolactin biosynthetic gene cluster (100% of genes show similarity)	-
Cluster 7	Terpene	20740	-	-
Cluster 8	Otherks	41244	Butirosin biosynthetic gene cluster (7% of genes show similarity)	-
Cluster 9	Nrps	65407	Surfactin biosynthetic gene cluster (91% of genes show similarity)	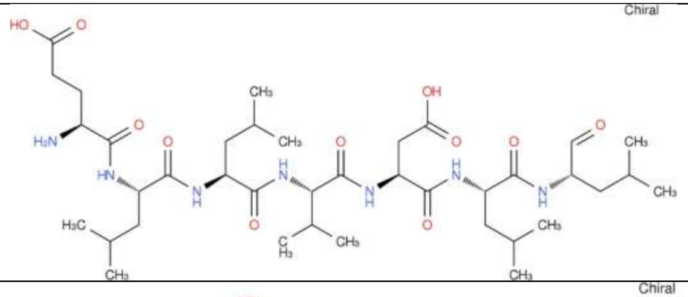
Cluster 10	Transatpks-Nrps	77730	Rhizocticin biosynthetic gene cluster (22% of genes show similarity)	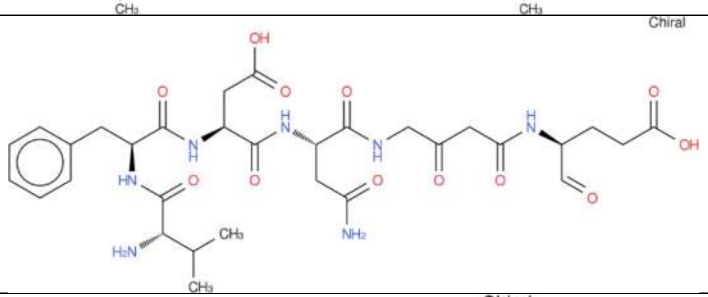
Cluster 11	Bacteriocin-Nrps	66794	Bacillibactin biosynthetic gene cluster (100% of genes show similarity)	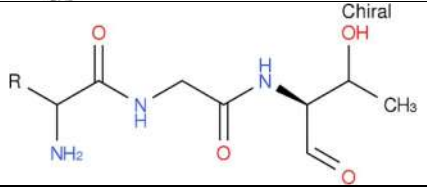

Table S1. Continued.

Strain clusters	Type	Length (bp)	Most similar known clusters	Predicted core clusters
**SYBC_H47**				
Cluster 12	Nrps	68423	-	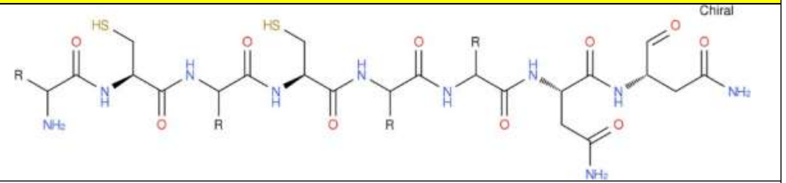
Cluster 13	Other	41418	Bacilysin biosynthetic gene cluster (100% of genes show similarity)	-

Table S1. Continued.

Strain clusters	Type	Length (bp)	Most similar known clusters	Predicted core clusters
**TrigoCor1448**				
Cluster 1	Nrps	65407	Surfactin biosynthetic gene cluster (91% of genes show similarity)	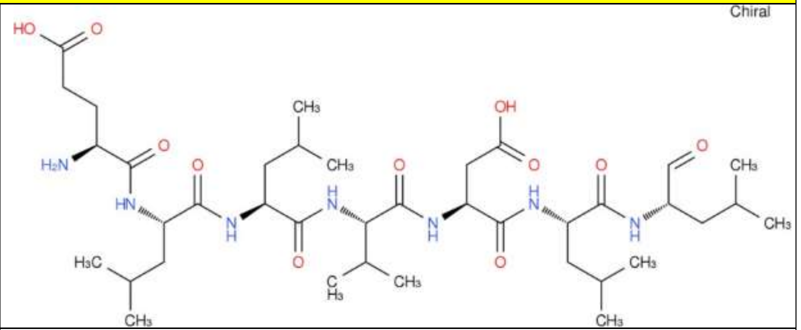
Cluster 2	Otherks	41244	Butirosin biosynthetic gene cluster (7% of genes show similarity)	-
Cluster 3	Terpene	20740	-	-
Cluster 4	Transatpks	85905	Macrolactin biosynthetic gene cluster (100% of genes show similarity)	-
Cluster 5	Transatpks-Nrps	102692	Bacillaene biosynthetic gene cluster (100% of genes show similarity)	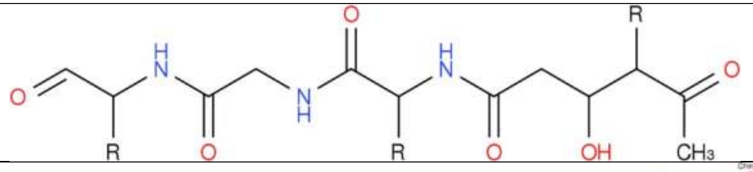
Cluster 6	Transatpks-Nrps	137813	Fengycin biosynthetic gene cluster (100% of genes show similarity)	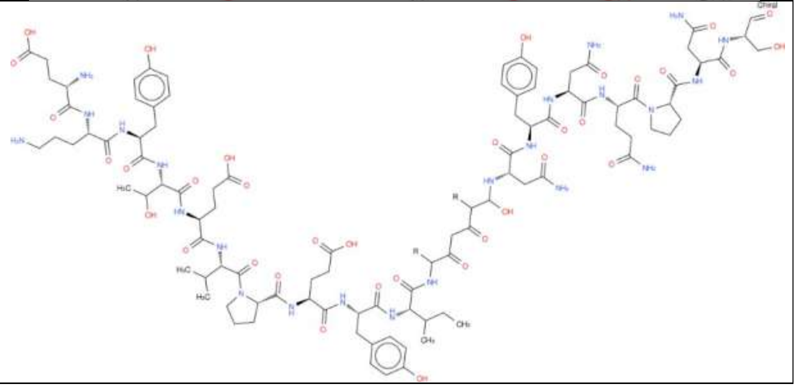

Table S1. Continued.

Strain clusters	Type	Length (bp)	Most similar known clusters	Predicted core clusters
**TrigoCor1448**				
Cluster 7	Terpene	21883	-	-
Cluster 8	T3pks	41100	-	-
Cluster 9	Transatpks	100455	Difficidin biosynthetic gene cluster (100% of genes show similarity)	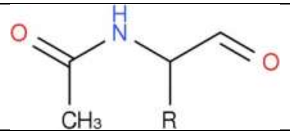
Cluster 10	Nrps	52666	Micrococcin P1 biosynthetic gene cluster (8% of genes show similarity)	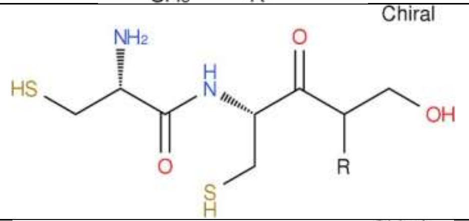
Cluster 11	Bacteriocin-Nrps	66791	Bacillibactin biosynthetic gene cluster (100% of genes show similarity)	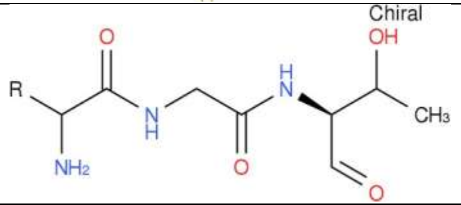
Cluster 12	Other	41418	Bacilysin biosynthetic gene cluster (100% of genes show similarity)	-

Table S1. Continued.

Strain clusters	Type	Length (bp)	Most similar known clusters	Predicted core clusters
**SSBW-19**				
Cluster 1	Other	41418	Bacilysin biosynthetic gene cluster (100% of genes show similarity)	-
Cluster 2	Bacteriocin-Nrps	66794	Bacillibactin biosynthetic gene cluster (100% of genes show similarity)	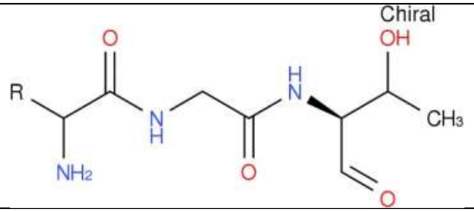
Cluster 3	Otherks	41244	Butirosin biosynthetic gene cluster (7% of genes show similarity)	-
Cluster 4	Terpene	20740	-	-
Cluster 5	Transatpks	85885	Macrolactin biosynthetic gene cluster (100% of genes show similarity)	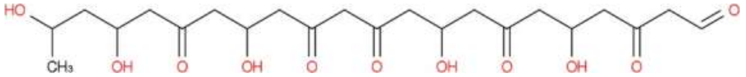
Cluster 6	Transatpks-Nrps	102677	Bacillaene biosynthetic gene cluster (100% of genes show similarity)	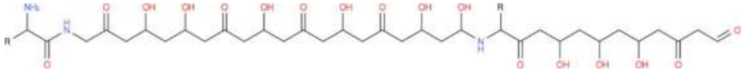
Cluster 7	Transatpks-Nrps	87842	Fengycin biosynthetic gene cluster (80% of genes show similarity)	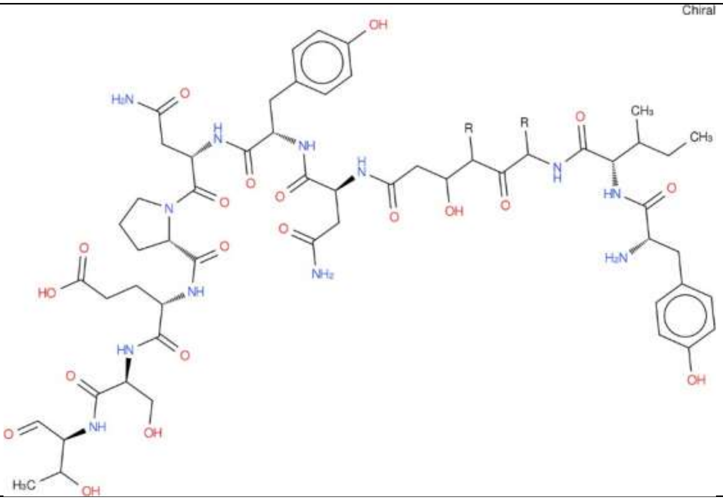

Table S1. Continued.

Strain clusters	Type	Length (bp)	Most similar known clusters	Predicted core clusters
**SSBW-19**				
Cluster 8	Nrps	37950	Plipastatin biosynthetic gene cluster (46% of genes show similarity)	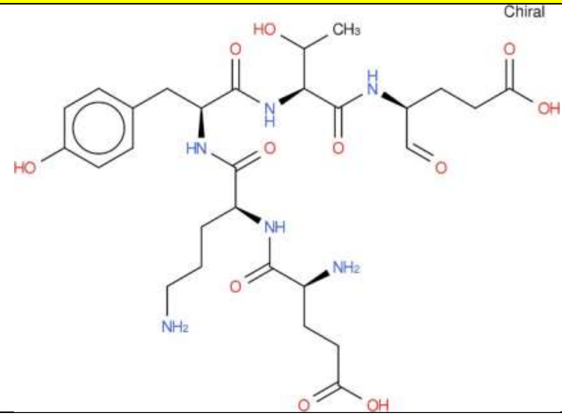
Cluster 9	Terpene	21883	-	-
Cluster 10	T3pks	41100	-	-
Cluster 11	Transatpks	100459	Difficidin biosynthetic gene cluster (100% of genes show similarity)	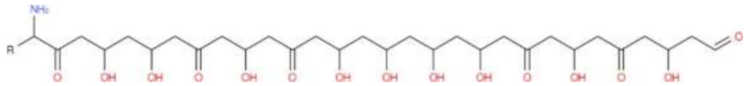
Cluster 12	Nrps	65407	Surfactin biosynthetic gene cluster (78% of genes show similarity)	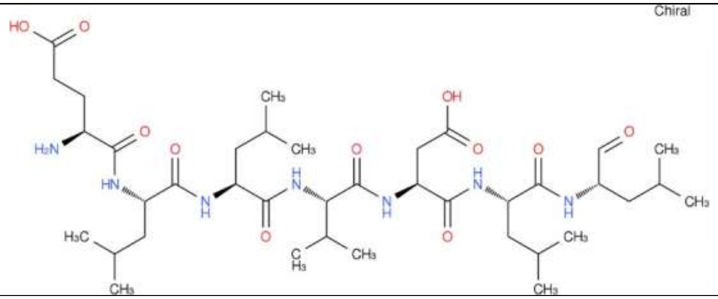
Cluster 13	Lantipeptide	18500	-	-
Cluster 14	Nrps	9486	Fengycin biosynthetic gene cluster (20% of genes show similarity)	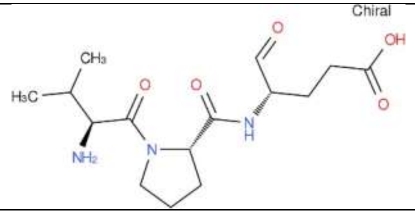

Table S1. Continued.

Strain clusters	Type	Length (bp)	Most similar known clusters	Predicted core clusters
**SSBW-19**				
Cluster 15	Nrps	1525	-	-

Table S1. Continued.

Strain clusters	Type	Length (bp)	Most similar known clusters	Predicted core clusters
**sx01604**				
Cluster 1	Other	41418	Bacilysin biosynthetic gene cluster (100% of genes show similarity)	-
Cluster 2	Bacteriocin-Nrps	66791	Bacillibactin biosynthetic gene cluster (100% of genes show similarity)	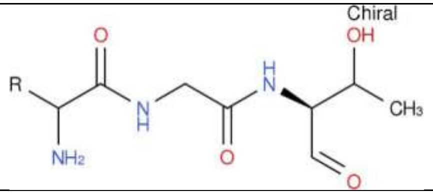
Cluster 3	Transatpks	96784	Difficidin biosynthetic gene cluster (66% of genes show similarity)	-
Cluster 4	T3pks	41100	-	-
Cluster 5	Terpene	21883	-	-
Cluster 6	Transatpks-Nrps	137801	Fengycin biosynthetic gene cluster (100% of genes show similarity)	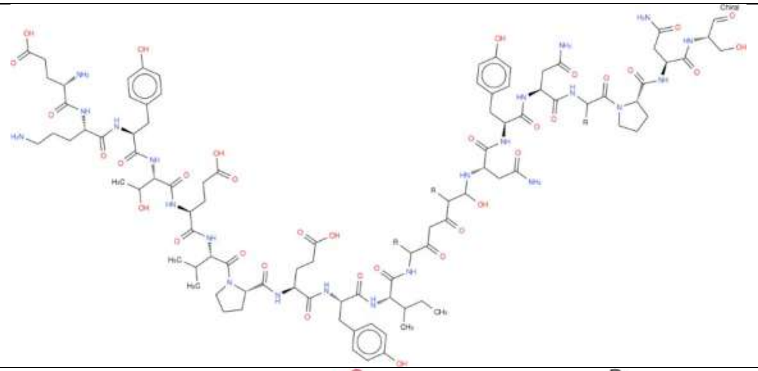
Cluster 7	Transatpks-Nrps	102674	Bacillaene biosynthetic gene cluster (100% of genes show similarity)	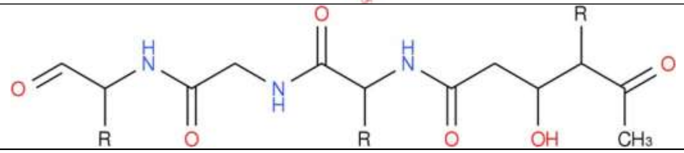
Cluster 8	Transatpks	85905	Macrolactin biosynthetic gene cluster (100% of genes show similarity)	-
Cluster 9	Lantipeptide	28888	-	-

Table S1. Continued.

Strain clusters	Type	Length (bp)	Most similar known clusters	Predicted core clusters
**sx01604**				
Cluster 10	Terpene	20740	-	-
Cluster 11	Otherks	41244	Butirosin biosynthetic gene cluster (7% of genes show similarity)	-
Cluster 12	Nrps	65407	Surfactin biosynthetic gene cluster (82% of genes show similarity)	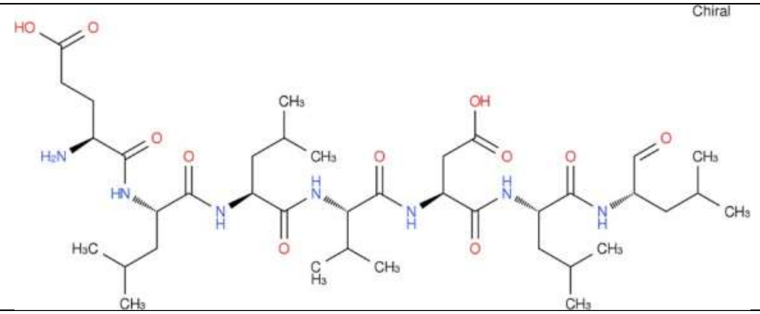

Table S1. Continued.

Strain clusters	Type	Length (bp)	Most similar known clusters	Predicted core clusters
**SSBW-18**				
Cluster 1	Otherks	41244	Butirosin biosynthetic gene cluster (7% of genes show similarity)	-
Cluster 2	Terpene	20740	-	-
Cluster 3	Transatpks	85887	Macrolactin biosynthetic gene cluster (100% of genes show similarity)	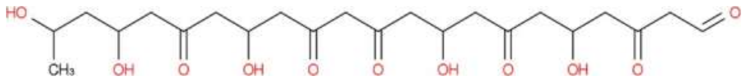
Cluster 4	Transatpks-Nrps	102692	Bacillaene biosynthetic gene cluster (100% of genes show similarity)	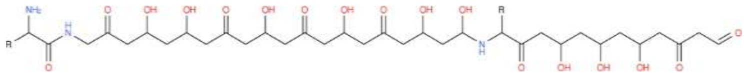
Cluster 5	Transatpks-Nrps	115398	Fengycin biosynthetic gene cluster (93% of genes show similarity)	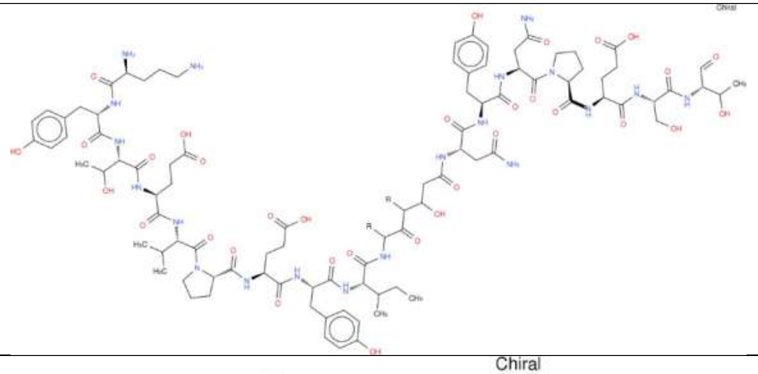
Cluster 6	Bacteriocin-Nrps	66793	Bacillibactin biosynthetic gene cluster (100% of genes show similarity)	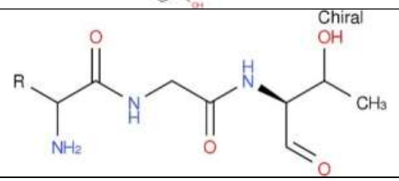
Cluster 7	Other	41418	Bacilysin biosynthetic gene cluster (100% of genes show similarity)	-
Cluster 8	Nrps	21974	Plipastatin biosynthetic gene cluster (30% of genes show similarity)	-
Cluster 9	Terpene	21883	-	-
Cluster 10	T3pks	41100	-	-

Table S1. Continued.

Strain clusters	Type	Length (bp)	Most similar known clusters	Predicted core clusters
**SSBW-18**				
Cluster 11	Transatpks	100453	Difficidin biosynthetic gene cluster (100% of genes show similarity)	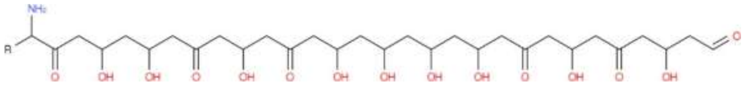
Cluster 12	Nrps	25942	Surfactin biosynthetic gene cluster (39% of genes show similarity)	-
Cluster 13	Nrps	27929	Surfactin biosynthetic gene cluster (47% of genes show similarity)	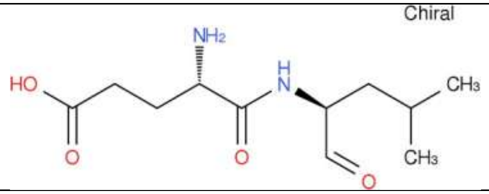
Cluster 14	Lantipeptide	22609	Locillomycin biosynthetic gene cluster (35% of genes show similarity)	-
Cluster 15	Nrps	9998	Surfactin biosynthetic gene cluster (8% of genes show similarity)	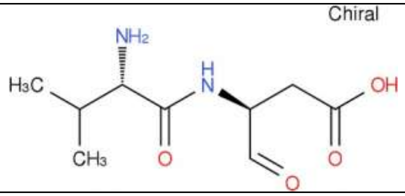

Table S1. Continued.

Strain clusters	Type	Length (bp)	Most similar known clusters	Predicted core clusters
**SSBW-8**				
Cluster 1	Otherks	41244	Butirosin biosynthetic gene cluster (7% of genes show similarity)	-
Cluster 2	Terpene	20740	-	-
Cluster 3	Transatpks	85885	Macrolactin biosynthetic gene cluster (100% of genes show similarity)	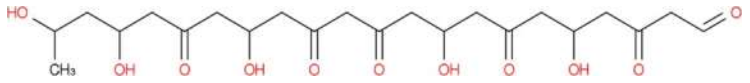
Cluster 4	Transatpks-Nrps	102677	Bacillaene biosynthetic gene cluster (100% of genes show similarity)	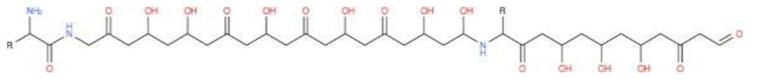
Cluster 5	Transatpks-Nrps	88472	Fengycin biosynthetic gene cluster (86% of genes show similarity)	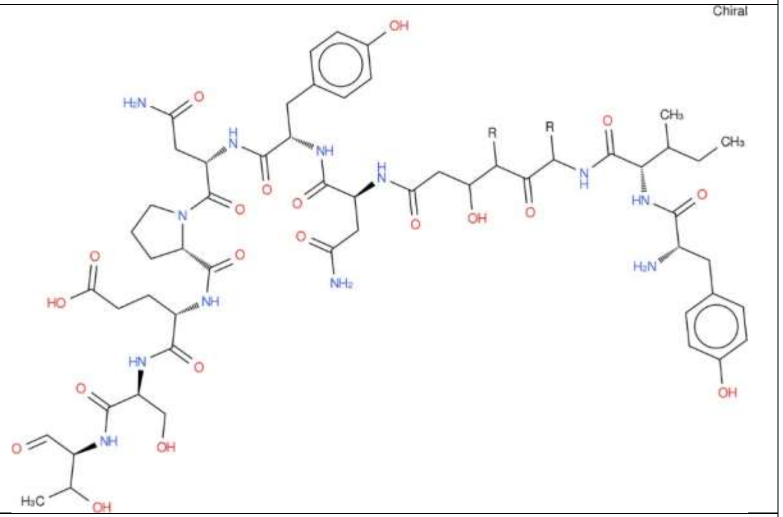
Cluster 6	Other	41418	Bacilysin biosynthetic gene cluster (100% of genes show similarity)	-
Cluster 7	Bacteriocin-Nrps	66794	Bacillibactin biosynthetic gene cluster (100% of genes show similarity)	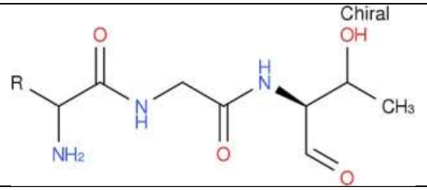

Table S1. Continued.

Strain clusters	Type	Length (bp)	Most similar known clusters	Predicted core clusters
**SSBW-8**				
Cluster 8	T3pks	41100	-	-
Cluster 9	Transatpks	100459	Difficidin biosynthetic gene cluster (100% of genes show similarity)	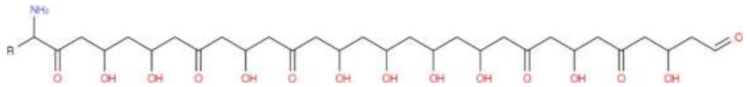
Cluster 10	Nrps	65407	Surfactin biosynthetic gene cluster (78% of genes show similarity)	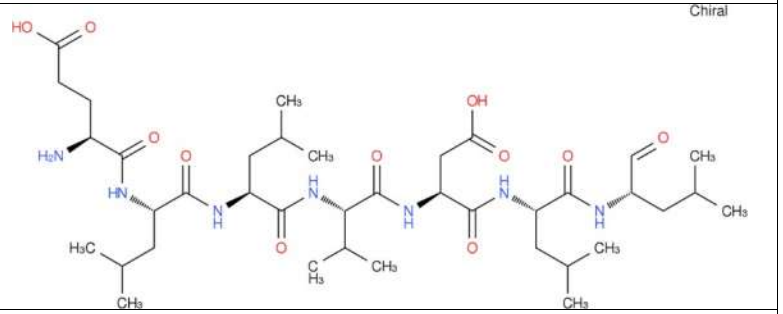
Cluster 11	Terpene	21883	-	-
Cluster 12	Nrps	38094	Plipastatin biosynthetic gene cluster (46% of genes show similarity)	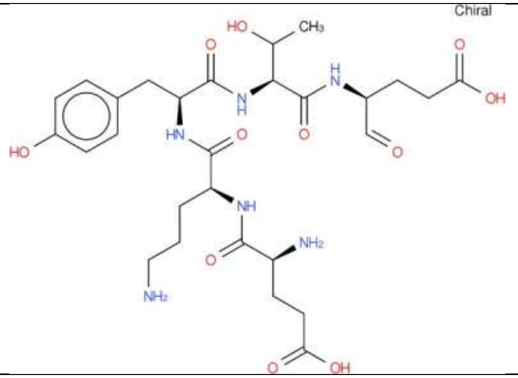
Cluster 13	Lantipeptide	18500	-	-
Cluster 14	Nrps	10260	Fengycin biosynthetic gene cluster (20% of genes show similarity)	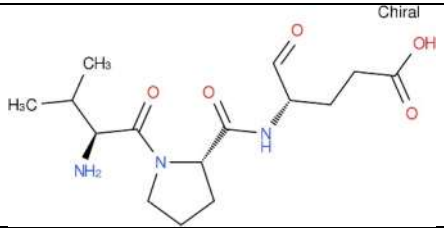

Table S1. Continued.

Strain clusters	Type	Length (bp)	Most similar known clusters	Predicted core clusters
**SSBW-10**				
Cluster 1	Otherks	41244	Butirosin biosynthetic gene cluster (7% of genes show similarity)	-
Cluster 2	Terpene	20740	-	-
Cluster 3	Transatpks	85885	Macrolactin biosynthetic gene cluster (100% of genes show similarity)	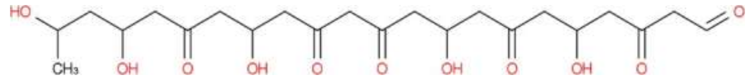
Cluster 4	Transatpks-Nrps	102677	Bacillaene biosynthetic gene cluster (100% of genes show similarity)	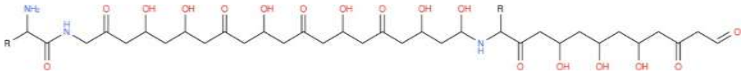
Cluster 5	Transatpks-Nrps	88472	Fengycin biosynthetic gene cluster (86% of genes show similarity)	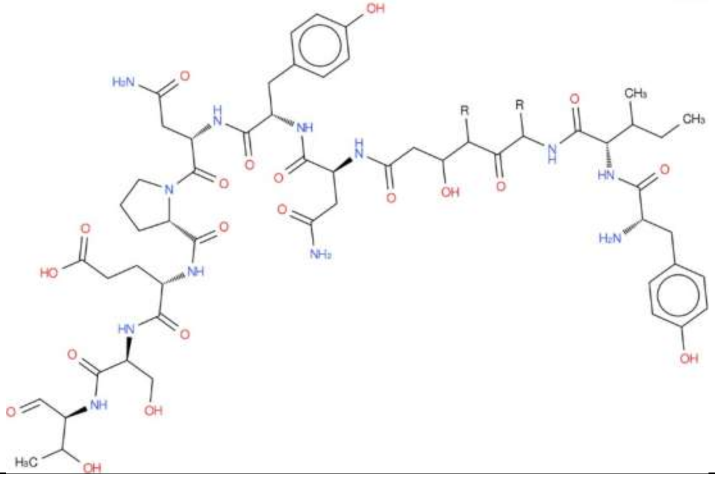
Cluster 6	Other	41418	Bacilysin biosynthetic gene cluster (100% of genes show similarity)	-
Cluster 7	Bacteriocin-Nrps	66794	Bacillibactin biosynthetic gene cluster (100% of genes show similarity)	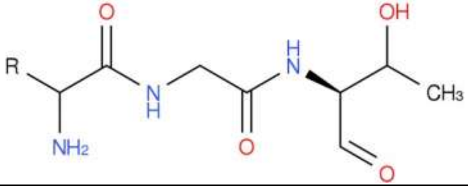

Table S1. Continued.

Strain clusters	Type	Length (bp)	Most similar known clusters	Predicted core clusters
**SSBW-10**				
Cluster 8	Nrps	38094	Plipastatin biosynthetic gene cluster (46% of genes show similarity)	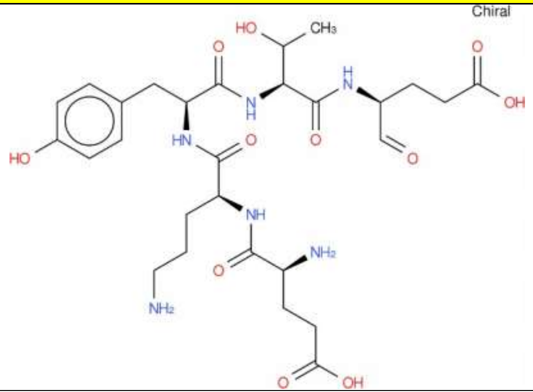
Cluster 9	Terpene	21883	-	-
Cluster 10	T3pks	41100	-	-
Cluster 11	Transatpks	100459	Difficidin biosynthetic gene cluster (100% of genes show similarity)	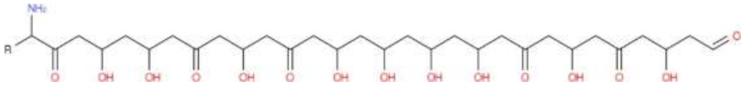
Cluster 12	Nrps	65407	Surfactin biosynthetic gene cluster (78% of genes show similarity)	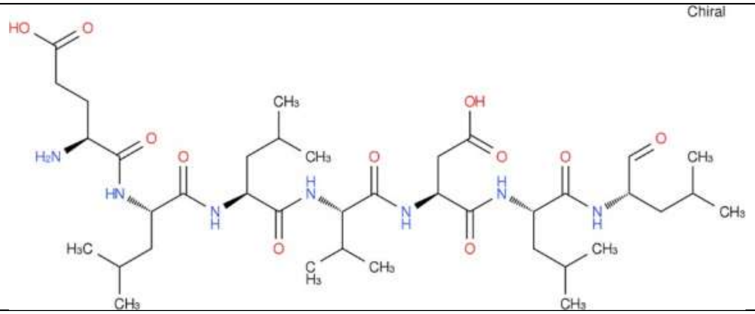
Cluster 13	Lantipeptide	18500	-	-
Cluster 14	Nrps	10260	Fengycin biosynthetic gene cluster (20% of genes show similarity)	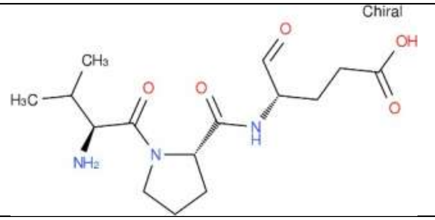

Table S1. Continued.

Strain clusters	Type	Length (bp)	Most similar known clusters	Predicted core clusters
**SSBW-2**				
Cluster 1	Otherks	41244	Butirosin biosynthetic gene cluster (7% of genes show similarity)	-
Cluster 2	Terpene	20740	-	-
Cluster 3	Transatpks	85885	Macrolactin biosynthetic gene cluster (100% of genes show similarity)	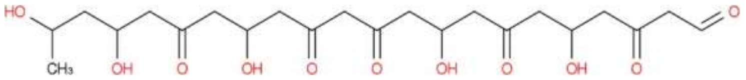
Cluster 4	Transatpks-Nrps	102677	Bacillaene biosynthetic gene cluster (100% of genes show similarity)	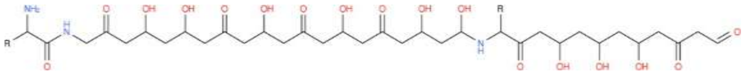
Cluster 5	Transatpks-Nrps	88472	Fengycin biosynthetic gene cluster (86% of genes show similarity)	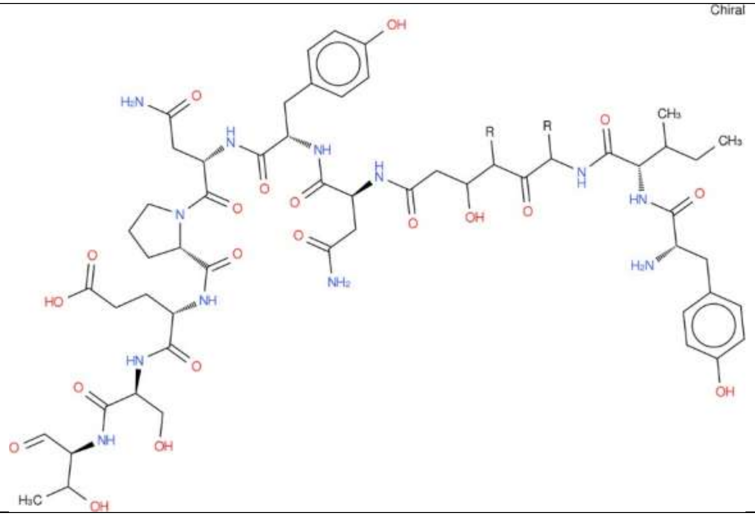
Cluster 6	Other	41418	Bacilysin biosynthetic gene cluster (100% of genes show similarity)	-
Cluster 7	Bacteriocin-Nrps	66794	Bacillibactin biosynthetic gene cluster (100% of genes show similarity)	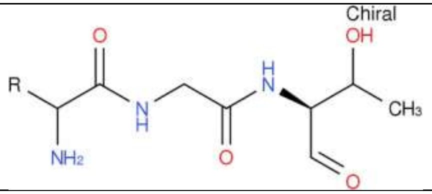

Table S1. Continued.

Strain clusters	Type	Length (bp)	Most similar known clusters	Predicted core clusters
**SSBW-2**				
Cluster 8	Nrps	38094	Plipastatin biosynthetic gene cluster (46% of genes show similarity)	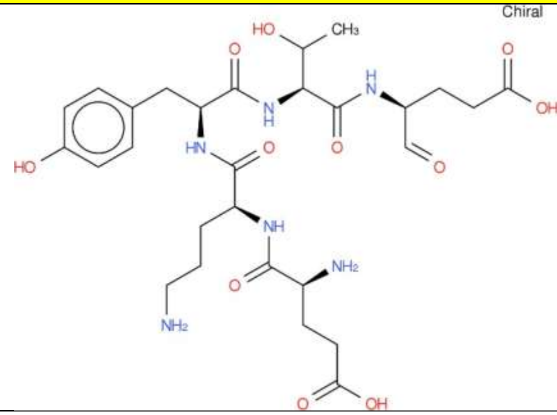
Cluster 9	Terpene	21883	-	-
Cluster 10	T3pks	41100	-	-
Cluster 11	Transatpks	100459	Difficidin biosynthetic gene cluster (100% of genes show similarity)	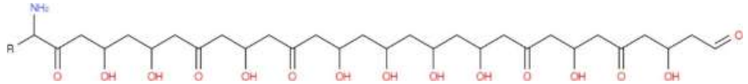
Cluster 12	Nrps	65407	Surfactin biosynthetic gene cluster (78% of genes show similarity)	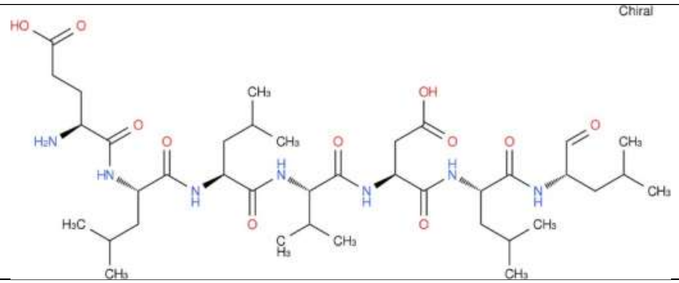
Cluster 13	Lantipeptide	18500	-	-
Cluster 14	Nrps	10260	Fengycin biosynthetic gene cluster (20% of genes show similarity)	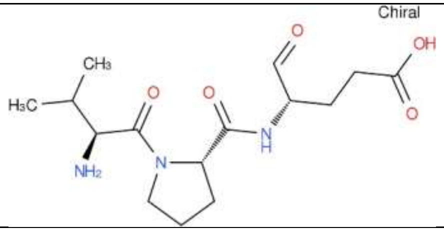

Table S1. Continued.

Strain clusters	Type	Length (bp)	Most similar known clusters	Predicted core clusters
**OEE-1**				
Cluster 1	Nrps	7846	-	-
Cluster 2	Nrps	10988	Surfactin biosynthetic gene cluster (8% of genes show similarity)	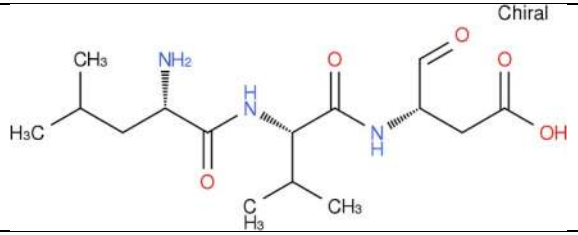
Cluster 3	Nrps	9364	-	-
Cluster 4	Transatpks-Nrps	23017	-	-
Cluster 5	Nrps	4955	-	-
Cluster 6	Terpene	20740	-	-
Cluster 7	Nrps	23700	Locillomycin biosynthetic gene cluster (35% of genes show similarity)	-
Cluster 8	Other	41418	Bacilysin biosynthetic gene cluster (100% of genes show similarity)	-
Cluster 9	Nrps	26782	Bacillibactin biosynthetic gene cluster (30% of genes show similarity)	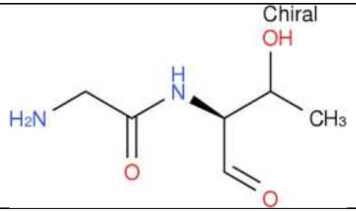
Cluster 10	Nrps	25628	Surfactin biosynthetic gene cluster (39% of genes show similarity)	-
Cluster 11	Nrps	27856	Surfactin biosynthetic gene cluster (43% of genes show similarity)	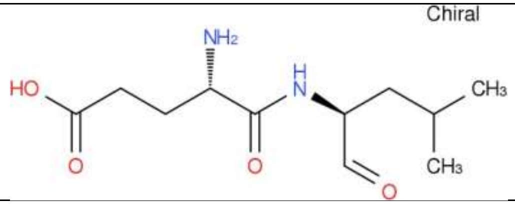

Table S1. Continued.

Strain clusters	Type	Length (bp)	Most similar known clusters	Predicted core clusters
**OEE-1**				
Cluster 12	Nrps	23107	Plipastatin biosynthetic gene cluster (38% of genes show similarity)	-
Cluster 13	Terpene	21883	-	-
Cluster 14	Transatpks-Nrps	82873	Fengycin biosynthetic gene cluster (66% of genes show similarity)	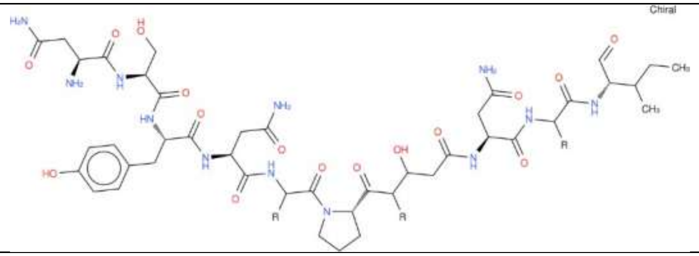
Cluster 15	Otherks	24921	Butirosin biosynthetic gene cluster (7% of genes show similarity)	-
Cluster 16	Bacteriocin-Nrps	40625	Bacillibactin biosynthetic gene cluster (76% of genes show similarity)	-
Cluster 17	Transatpks	15750	Difficidin biosynthetic gene cluster (33% of genes show similarity)	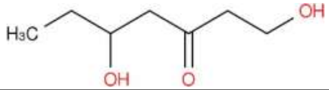
Cluster 18	Nrps	2469	-	-
Cluster 19	Lantipeptide	28889	-	-
Cluster 20	Transatpks-Nrps	35059	Bacillaene biosynthetic gene cluster (28% of genes show similarity)	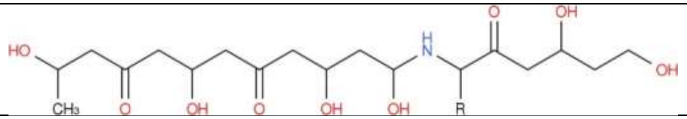
Cluster 21	Nrps	6118	Fengycin biosynthetic gene cluster (26% of genes show similarity)	-
Cluster 22	Nrps	14974	Plipastatin biosynthetic gene cluster (23% of genes show similarity)	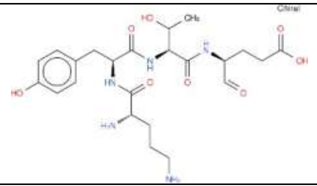

Table S1. Continued.

Strain clusters	Type	Length (bp)	Most similar known clusters	Predicted core clusters
**OEE-1**				
Cluster 23	Transatpks-Nrps	13475	Bacillaene biosynthetic gene cluster (21% of genes show similarity)	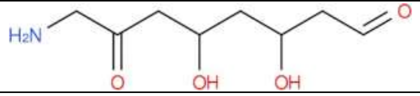
Cluster 24	Nrps	2552	-	-
Cluster 25	Transatpks	75766	Difficidin biosynthetic gene cluster (86% of genes show similarity)	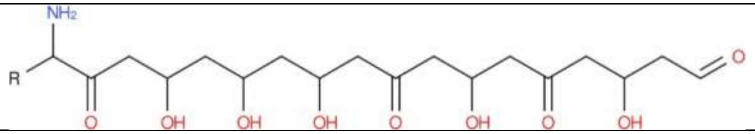
Cluster 26	Transatpks	7967	-	-
Cluster 27	Transatpks	32457	Bacillaene biosynthetic gene cluster (28% of genes show similarity)	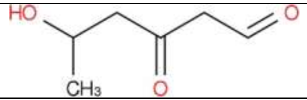
Cluster 28	T3pks	41100	-	-
Cluster 29	Transatpks	85887	Macrolactin biosynthetic gene cluster (100% of genes show similarity)	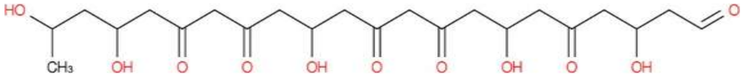

Table S1. Continued.

Strain clusters	Type	Length (bp)	Most similar known clusters	Predicted core clusters
**NRRL_B-41580**				
Cluster 1	Nrps	27617	Surfactin biosynthetic gene cluster (47% of genes show similarity)	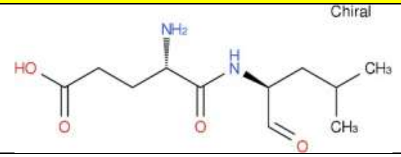
Cluster 2	Nrps	25178	Surfactin biosynthetic gene cluster (39% of genes show similarity)	-
Cluster 3	Nrps	14787	Fengycin biosynthetic gene cluster (26% of genes show similarity)	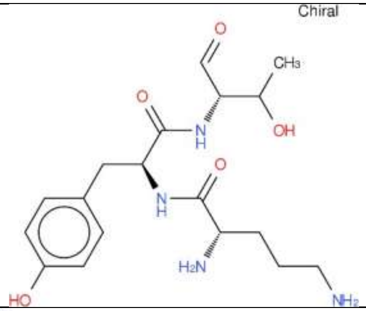
Cluster 4	Transatpks	42887	Bacillaene biosynthetic gene cluster (28% of genes show similarity)	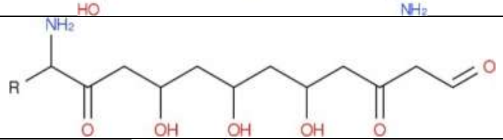
Cluster 5	Transatpks	100453	Difficidin biosynthetic gene cluster (100% of genes show similarity)	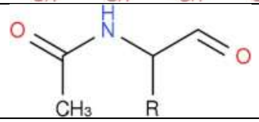
Cluster 6	T3pks	41100	-	-
Cluster 7	Terpene	21883	-	-
Cluster 8	Nrps	2334	-	-
Cluster 9	Nrps	1883	-	-
Cluster 10	Bacteriocin-Nrps	66793	Bacillibactin biosynthetic gene cluster (100% of genes show similarity)	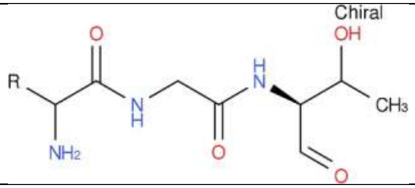

Table S1. Continued.

Strain clusters	Type	Length (bp)	Most similar known clusters	Predicted core clusters
**NRRL_B-41580**				
Cluster 11	Transatpks-Nrps	59808	Bacillaene biosynthetic gene cluster (85% of genes show similarity)	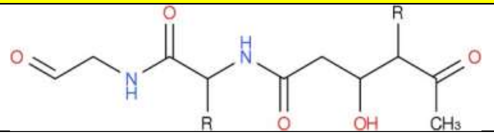
Cluster 12	Transatpks	85902	Macrolactin biosynthetic gene cluster (100% of genes show similarity)	-
Cluster 13	Terpene	20740	-	-
Cluster 14	Otherks	41244	Butirosin biosynthetic gene cluster (7% of genes show similarity)	-
Cluster 15	Nrps	40295	-	-
Cluster 16	Other	41418	Bacilysin biosynthetic gene cluster (100% of genes show similarity)	-
Cluster 17	Lantipeptide	23984	Mersacidin biosynthetic gene cluster (100% of genes show similarity)	-
Cluster 18	Transatpks-Nrps	100306	Fengycin biosynthetic gene cluster (86% of genes show similarity)	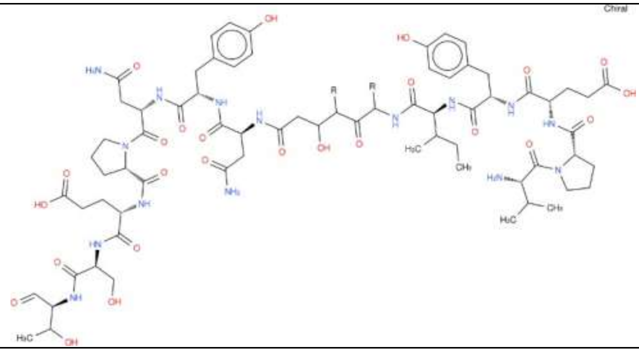
Cluster 19	Phosphonate	40884	-	-
Cluster 20	Nrps	8922	Surfactin biosynthetic gene cluster (8% of genes show similarity)	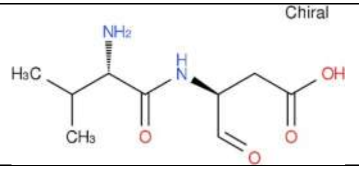

Table S1. Continued.

Strain clusters	Type	Length (bp)	Most similar known clusters	Predicted core clusters
**SRCM100731**				
Cluster 1	Transatpks	85902	Macrolactin biosynthetic gene cluster (100% of genes show similarity)	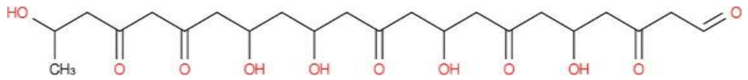
Cluster 2	Transatpks	22266	Bacillaene biosynthetic gene cluster (78% of genes show similarity)	-
Cluster 3	Other	41418	Bacilysin biosynthetic gene cluster (100% of genes show similarity)	-
Cluster 4	Bacteriocin	13078	-	-
Cluster 5	Lantipeptide	28889	-	-
Cluster 6	Bacteriocin-Nrps	66791	Bacillibactin biosynthetic gene cluster (100% of genes show similarity)	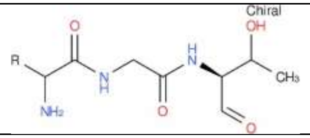
Cluster 7	Transatpks	75781	Difficidin biosynthetic gene cluster (86% of genes show similarity)	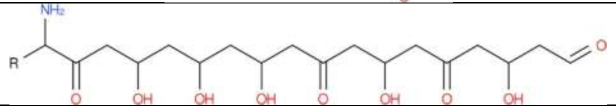
Cluster 8	Terpene	21883	-	-
Cluster 9	Terpene	20740	-	-
Cluster 10	Otherks	41244	Butirosin biosynthetic gene cluster (7% of genes show similarity)	-
Cluster 11	Lassopeptide	22221	-	-
Cluster 12	Transatpks-Nrps	60356	Bacillaene biosynthetic gene cluster (42% of genes show similarity)	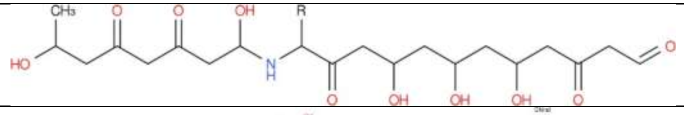
Cluster 13	Transatpks-Nrps	134616	Fengycin biosynthetic gene cluster (93% of genes show similarity)	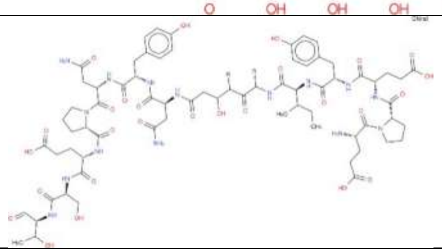

Table S1. Continued.

Strain clusters	Type	Length (bp)	Most similar known clusters	Predicted core clusters
**SRCM100731**				
Cluster 14	Thiopeptide	22491	-	-
Cluster 15	Nrps	65407	Surfactin biosynthetic gene cluster (82% of genes show similarity)	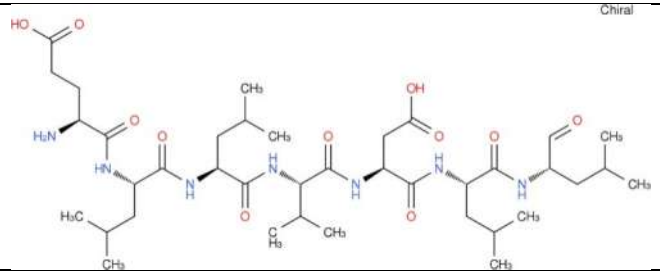

Table S1. Continued.

Strain clusters	Type	Length (bp)	Most similar known clusters	Predicted core clusters
**SRCM100730**				
Cluster 1	Bacteriocin	13078	-	-
Cluster 2	Other	41418	Bacilysin biosynthetic gene cluster (100% of genes show similarity)	-
Cluster 3	Transatpks	75781	Difficidin biosynthetic gene cluster (86% of genes show similarity)	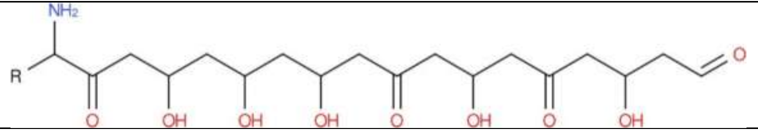
Cluster 4	Terpene	20740	-	-
Cluster 5	Lantipeptide	28889	-	-
Cluster 6	Thiopeptide	22368	-	-
Cluster 7	Terpene	21883	-	-
Cluster 8	Otherks	41244	Butirosin biosynthetic gene cluster (7% of genes show similarity)	
Cluster 9	Lassopeptide	22221	-	-
Cluster 10	Transatpks	22266	Bacillaene biosynthetic gene cluster (78% of genes show similarity)	-
Cluster 11	Transatpks	85902	Macrolactin biosynthetic gene cluster (100% of genes show similarity)	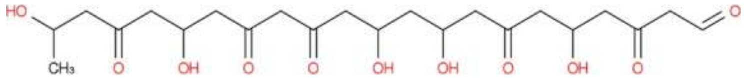
Cluster 12	Transatpks-Nrps	134616	Fengycin biosynthetic gene cluster (93% of genes show similarity)	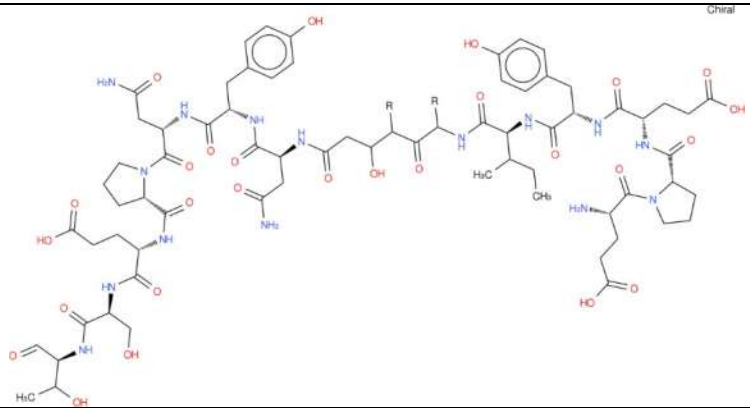

Table S1. Continued.

Strain clusters	Type	Length (bp)	Most similar known clusters	Predicted core clusters
**SRCM100730**				
Cluster 13	Transatpks-Nrps	60217	Bacillaene biosynthetic gene cluster (42% of genes show similarity)	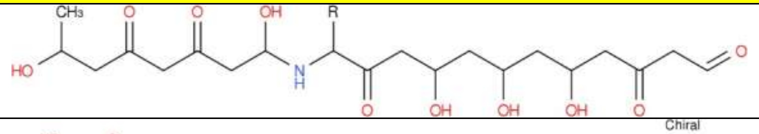
Cluster 14	Nrps	65407	Surfactin biosynthetic gene cluster (82% of genes show similarity)	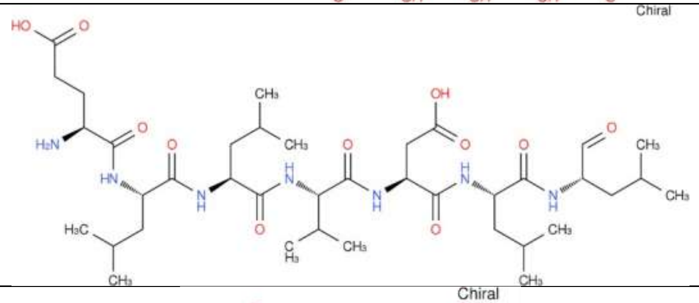
Cluster 15	Bacteriocin-Nrps	66791	Bacillibactin biosynthetic gene cluster (100% of genes show similarity)	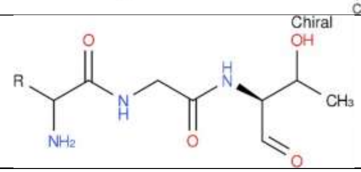

Table S1. Continued.

Strain clusters	Type	Length (bp)	Most similar known clusters	Predicted core clusters
**SQR9**				
Cluster 1	Nrps	65407	Surfactin biosynthetic gene cluster (78% of genes show similarity)	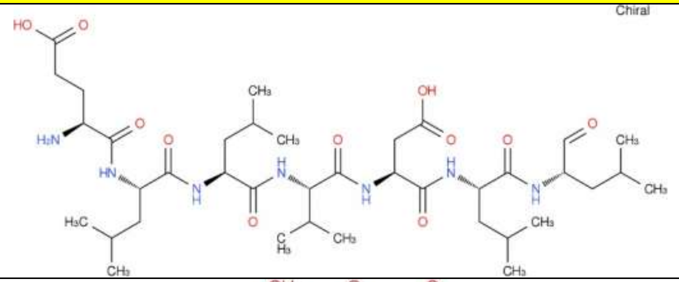
Cluster 2	Transatpks-T1pks	110709	Phormidolide biosynthetic gene cluster (26% of genes show similarity)	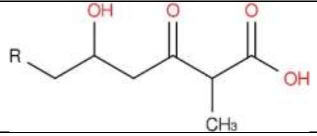
Cluster 3	Otherks	41244	Butirosin biosynthetic gene cluster (7% of genes show similarity)	-
Cluster 4	Terpene	20740	-	-
Cluster 5	Transatpks	85890	Macrolactin biosynthetic gene cluster (100% of genes show similarity)	-
Cluster 6	Transatpks-Nrps	102671	Bacillaene biosynthetic gene cluster (100% of genes show similarity)	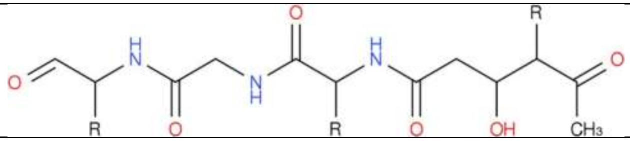
Cluster 7	Transatpks-Nrps	137813	Fengycin biosynthetic gene cluster (100% of genes show similarity)	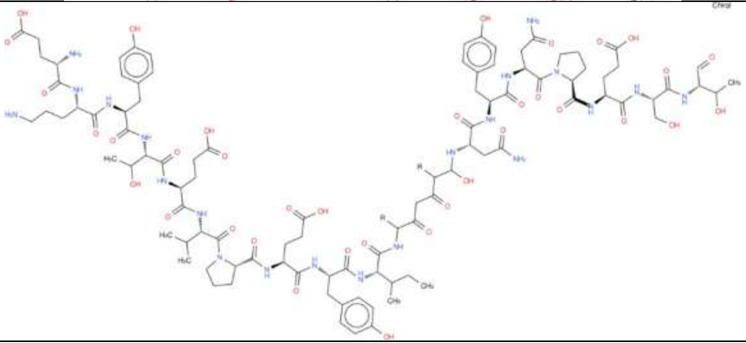

Table S1. Continued.

Strain clusters	Type	Length (bp)	Most similar known clusters	Predicted core clusters
**SQR9**				
Cluster 8	Terpene	21883	-	-
Cluster 9	T3pks	41100	-	-
Cluster 10	Transatpks	100453	Difficidin biosynthetic gene cluster (100% of genes show similarity)	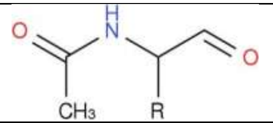
Cluster 11	Bacteriocin-Nrps	66791	Bacillibactin biosynthetic gene cluster (100% of genes show similarity)	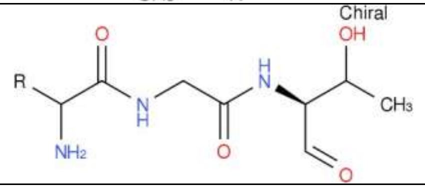
Cluster 12	Other	41418	Bacilysin biosynthetic gene cluster (100% of genes show similarity)	-

Table S1. Continued.

Strain clusters	Type	Length (bp)	Most similar known clusters	Predicted core clusters
**KCTC_13012**				
Cluster 1	Terpene	21883	-	-
Cluster 2	T3pks	41100	-	-
Cluster 3	Transatpks	100473	Difficidin biosynthetic gene cluster (100% of genes show similarity)	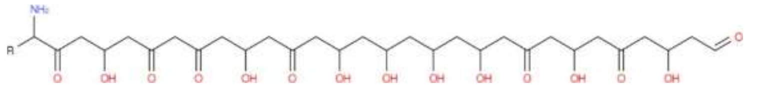
Cluster 4	Transatpks-Nrps	102677	Bacillaene biosynthetic gene cluster (100% of genes show similarity)	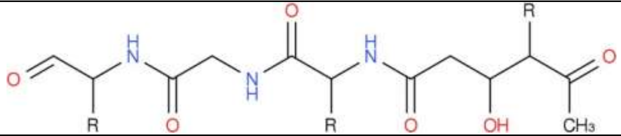
Cluster 5	Transatpks	85902	Macrolactin biosynthetic gene cluster (100% of genes show similarity)	-
Cluster 6	Terpene	20740	-	-
Cluster 7	Otherks	41244	Butirosin biosynthetic gene cluster (7% of genes show similarity)	-
Cluster 8	Transatpks-Nrps	88416	Fengycin biosynthetic gene cluster (86% of genes show similarity)	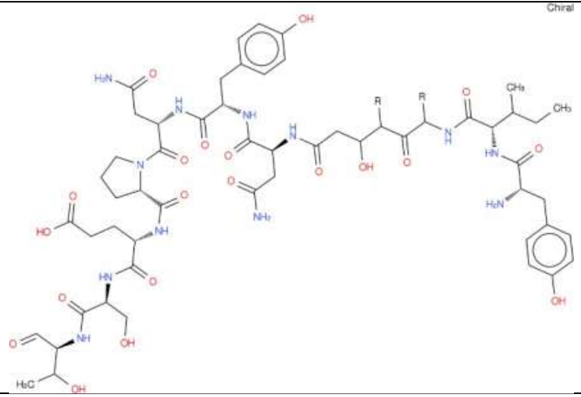
Cluster 9	Nrps	23335	-	-
Cluster 10	Bacteriocin-Nrps	66793	Bacillibactin biosynthetic gene cluster (100% of genes show similarity)	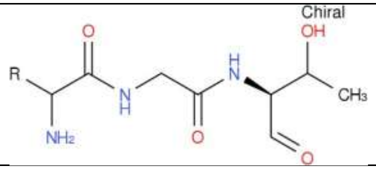

Table S1. Continued.

Strain clusters	Type	Length (bp)	Most similar known clusters	Predicted core clusters
**KCTC_13012**				
Cluster 11	Phosphonate	40884	-	-
Cluster 12	Lantipeptide	23984	Mersacidin biosynthetic gene cluster (100% of genes show similarity)	-
Cluster 13	Other	41418	Bacilysin biosynthetic gene cluster (100% of genes show similarity)	-
Cluster 14	Nrps	42050	-	-
Cluster 15	Nrps	27660	Surfactin biosynthetic gene cluster (47% of genes show similarity)	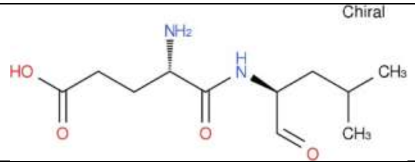
Cluster 16	Nrps	2587	-	-
Cluster 17	Nrps	25221	Surfactin biosynthetic gene cluster (39% of genes show similarity)	-
Cluster 18	Nrps	1961	-	-
Cluster 19	Nrps	14360	Fengycin biosynthetic gene cluster (33% of genes show similarity)	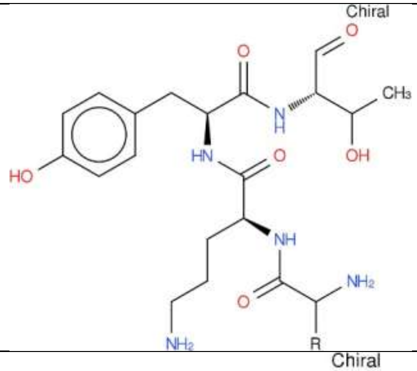
Cluster 20	Nrps	9008	Surfactin biosynthetic gene cluster (8% of genes show similarity)	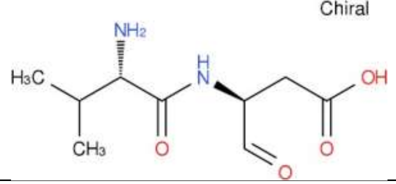

Table S1. Continued.

Strain clusters	Type	Length (bp)	Most similar known clusters	Predicted core clusters
**KCTC_13012**				
Cluster 21	Nrps	10378	Fengycin biosynthetic gene cluster (20% of genes show similarity)	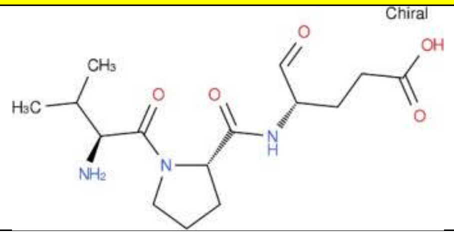
Cluster 22	Nrps	1883	-	-

Table S1. Continued.

Strain clusters	Type	Length (bp)	Most similar known clusters	Predicted core clusters
**NB91**				
Cluster 1	Otherks	41244	Butirosin biosynthetic gene cluster (7% of genes show similarity)	-
Cluster 2	Terpene	20740	-	-
Cluster 3	Transatpks	85884	Macrolactin biosynthetic gene cluster (100% of genes show similarity)	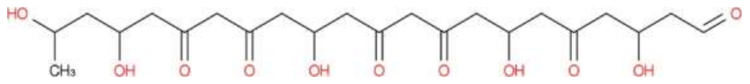
Cluster 4	Transatpks-Nrps	102698	Bacillaene biosynthetic gene cluster (100% of genes show similarity)	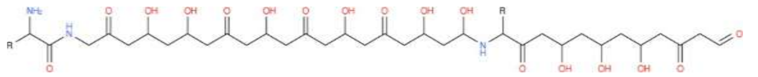
Cluster 5	Transatpks-Nrps	88134	Fengycin biosynthetic gene cluster (86% of genes show similarity)	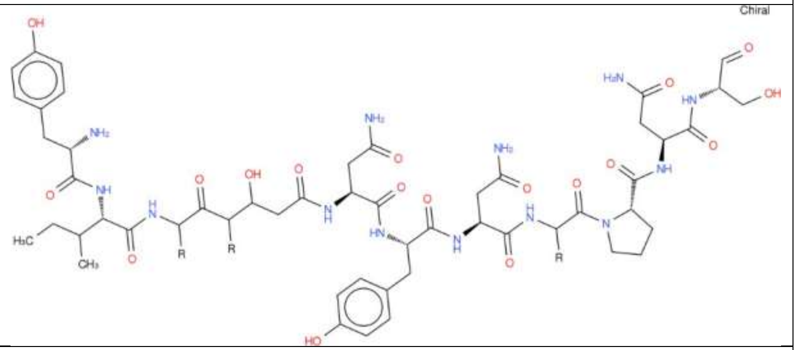
Cluster 6	Other	41418	Bacilysin biosynthetic gene cluster (100% of genes show similarity)	-
Cluster 7	Bacteriocin-Nrps	66792	Bacillibactin biosynthetic gene cluster (100% of genes show similarity)	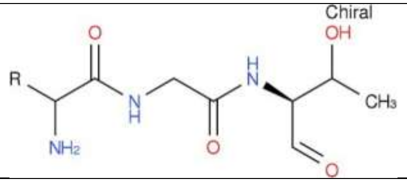
Cluster 8	Terpene	21883	-	-
Cluster 9	T3pks	41100	-	-
Cluster 10	Transatpks	100438	Difficidin biosynthetic gene cluster (100% of genes show similarity)	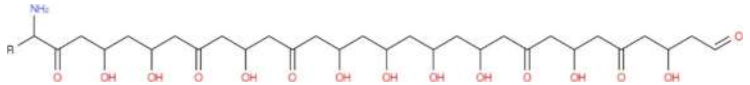

Table S1. Continued.

Strain clusters	Type	Length (bp)	Most similar known clusters	Predicted core clusters
**NB91**				
Cluster 11	Nrps	65407	Surfactin biosynthetic gene cluster (82% of genes show similarity)	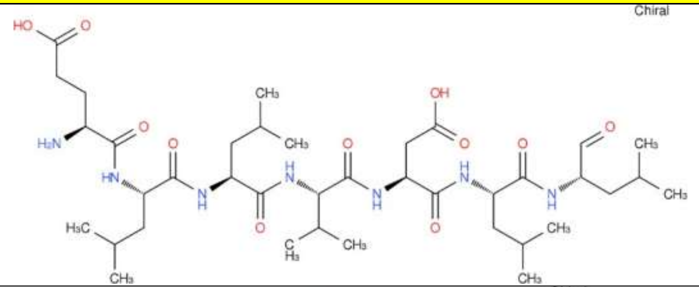
Cluster 12	Nrps	15277	Fengycin biosynthetic gene cluster (26% of genes show similarity)	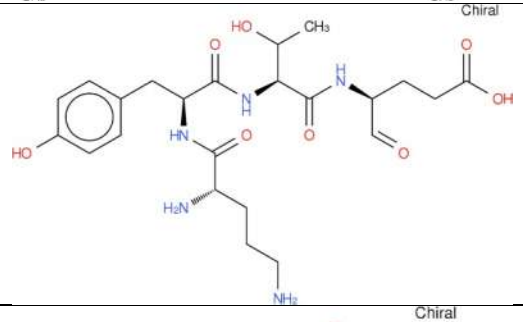
Cluster 13	Nrps	9685	Fengycin biosynthetic gene cluster (13% of genes show similarity)	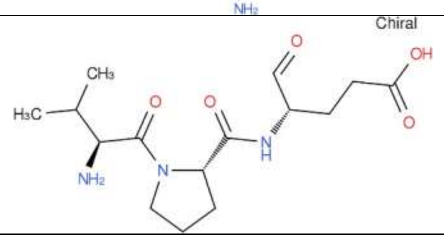
Cluster 14	Nrps	8965	Plipastatin biosynthetic gene cluster (30% of genes show similarity)	-

Table S1. Continued.

Strain clusters	Type	Length (bp)	Most similar known clusters	Predicted core clusters
**KACC_13105**				
Cluster 1	Nrps	36146	Surfactin biosynthetic gene cluster (39% of genes show similarity)	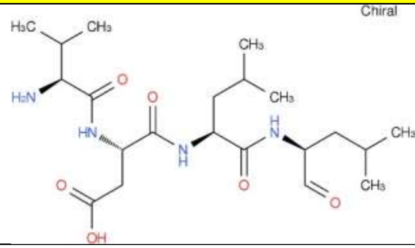
Cluster 2	Transatpks	85905	Macrolactin biosynthetic gene cluster (100% of genes show similarity)	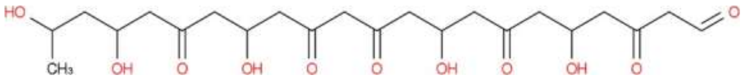
Cluster 3	Terpene	21883	-	-
Cluster 4	Nrps	23186	Plipastatin biosynthetic gene cluster (38% of genes show similarity)	-
Cluster 5	Transatpks-Nrps	114098	Fengycin biosynthetic gene cluster (93% of genes show similarity)	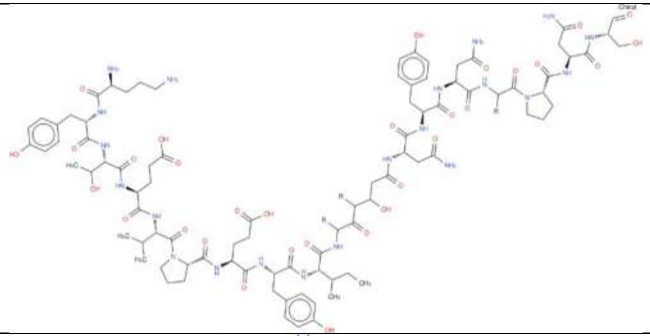
Cluster 6	Transatpks	100453	Difficidin biosynthetic gene cluster (100% of genes show similarity)	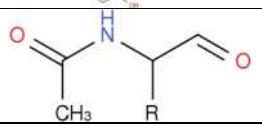
Cluster 7	T3pks	41100	-	-
Cluster 8	Bacteriocin-Nrps	66791	Bacillibactin biosynthetic gene cluster (100% of genes show similarity)	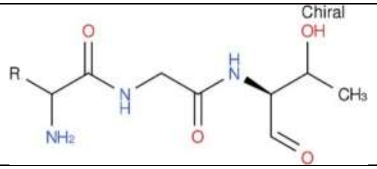

Table S1. Continued.

Strain clusters	Type	Length (bp)	Most similar known clusters	Predicted core clusters
**KACC_13105**				
Cluster 1	Nrps	36146	Surfactin biosynthetic gene cluster (39% of genes show similarity)	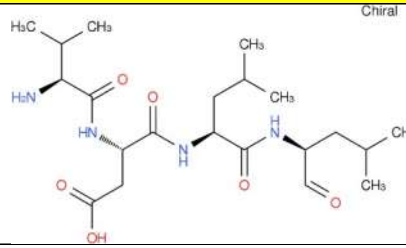
Cluster 2	Transatpks	85905	Macrolactin biosynthetic gene cluster (100% of genes show similarity)	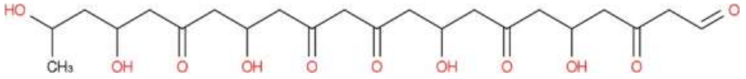
Cluster 3	Terpene	21883	-	-
Cluster 4	Nrps	23186	Plipastatin biosynthetic gene cluster (38% of genes show similarity)	-
Cluster 5	Transatpks-Nrps	114098	Fengycin biosynthetic gene cluster (93% of genes show similarity)	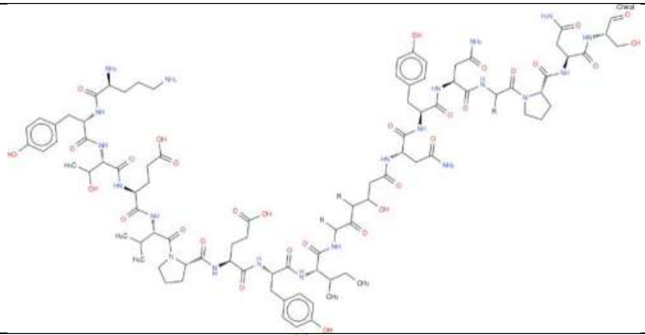
Cluster 6	Transatpks	100453	Difficidin biosynthetic gene cluster (100% of genes show similarity)	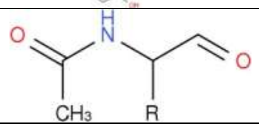
Cluster 7	T3pks	41100	-	-
Cluster 8	Bacteriocin-Nrps	66791	Bacillibactin biosynthetic gene cluster (100% of genes show similarity)	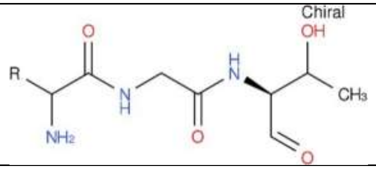

Table S1. Continued.

Strain clusters	Type	Length (bp)	Most similar known clusters	Predicted core clusters
**KACC_13105**				
Cluster 9	Other	41418	Bacilysin biosynthetic gene cluster (100% of genes show similarity)	-
Cluster 10	Transatpks-Nrps	102674	Bacillaene biosynthetic gene cluster (100% of genes show similarity)	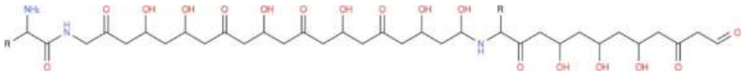
Cluster 11	Nrps	29170	Surfactin biosynthetic gene cluster (52% of genes show similarity)	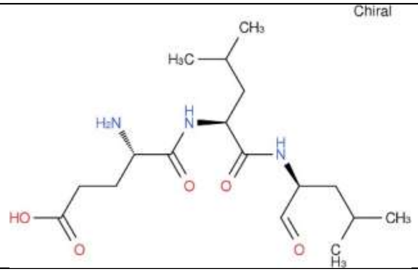
Cluster 12	Otherks	41244	Butirosin biosynthetic gene cluster (7% of genes show similarity)	-
Cluster 13	Terpene	20740	-	-
Cluster 14	Lantipeptide	28888	-	-

Table S1. Continued.

Strain clusters	Type	Length (bp)	Most similar known clusters	Predicted core clusters
**CFSAN034340**				
Cluster 1	Terpene	20740	-	-
Cluster 2	Transatpks	28132	Difficidin biosynthetic gene cluster (46% of genes show similarity)	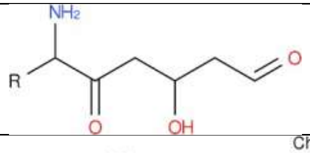
Cluster 3	Nrps	27638	Surfactin biosynthetic gene cluster (47% of genes show similarity)	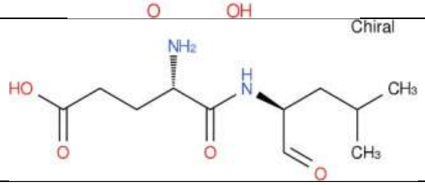
Cluster 4	Nrps	25179	Surfactin biosynthetic gene cluster (39% of genes show similarity)	-
Cluster 5	Other	41418	Bacilysin biosynthetic gene cluster (100% of genes show similarity)	-
Cluster 6	Bacteriocin-Nrps	66792	Bacillibactin biosynthetic gene cluster (100% of genes show similarity)	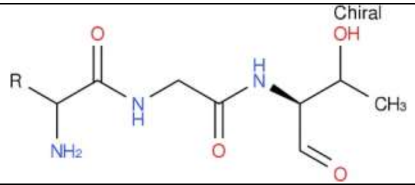
Cluster 7	Lantipeptide-Nrps	38496	Locillomycin biosynthetic gene cluster (35% of genes show similarity)	-
Cluster 8	Nrps	22475	Plipastatin biosynthetic gene cluster (30% of genes show similarity)	
Cluster 9	Terpene	21883	-	-
Cluster 10	T3pks	41100	-	-
Cluster 11	Transatpks	45652	Difficidin biosynthetic gene cluster (53% of genes show similarity)	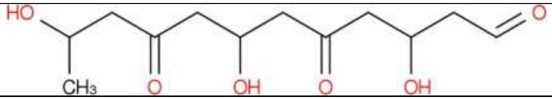
Cluster 12	Transatpks-Nrps	32808	-	-

Table S1. Continued.

Strain clusters	Type	Length (bp)	Most similar known clusters	Predicted core clusters
**CFSAN034340**				
Cluster 13	Transatpks	23278	Difficidin biosynthetic gene cluster (26% of genes show similarity)	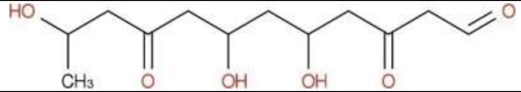
Cluster 14	Transatpks	86193	Macrolactin biosynthetic gene cluster (100% of genes show similarity)	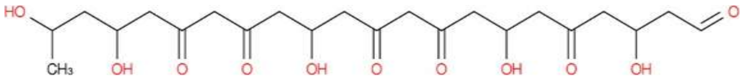
Cluster 15	Nrps	12704	Fengycin biosynthetic gene cluster (20% of genes show similarity)	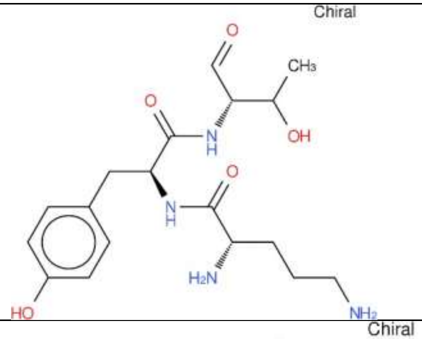
Cluster 16	Nrps	9119	Fengycin biosynthetic gene cluster (13% of genes show similarity)	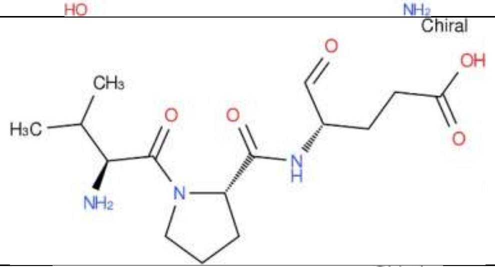
Cluster 17	Nrps	8944	Surfactin biosynthetic gene cluster (8% of genes show similarity)	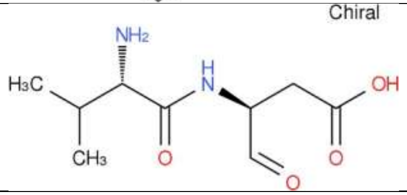
Cluster 18	Transatpks	8685	-	-
Cluster 19	Transatpks	7213	Bacillaene biosynthetic gene cluster (14% of genes show similarity)	-
Cluster 20	Nrps	6519	-	-

Table S1. Continued.

Strain clusters	Type	Length (bp)	Most similar known clusters	Predicted core clusters
**CFSAN034340**				
Cluster 21	Other	3084	-	-
Cluster 22	Transatpks	31155	Bacillaene biosynthetic gene cluster (28% of genes show similarity)	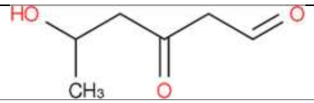
Cluster 23	Transatpks-Nrps	87560	Fengycin biosynthetic gene cluster (80% of genes show similarity)	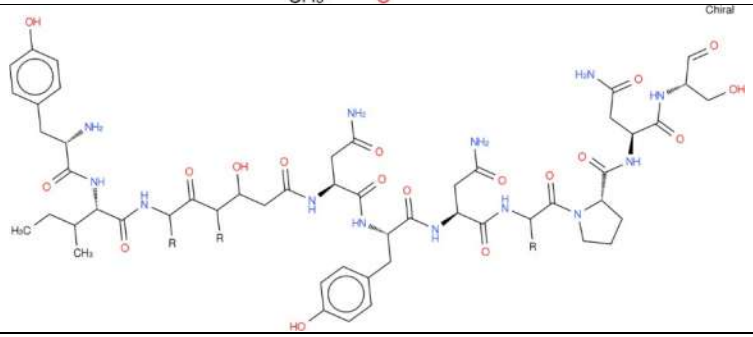
Cluster 24	Nrps	1863	-	-
Cluster 25	Otherks	41244	Butirosin biosynthetic gene cluster (7% of genes show similarity)	-
Cluster 26	Transatpks-Nrps	52635	Bacillaene biosynthetic gene cluster (85% of genes show similarity)	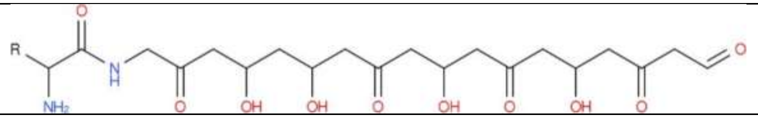

Table S1. Continued.

Strain clusters	Type	Length (bp)	Most similar known clusters	Predicted core clusters
**NJN-6**				
Cluster 1	Transatpks-Nrps	77723	Rhizocticin biosynthetic gene cluster (22% of genes show similarity)	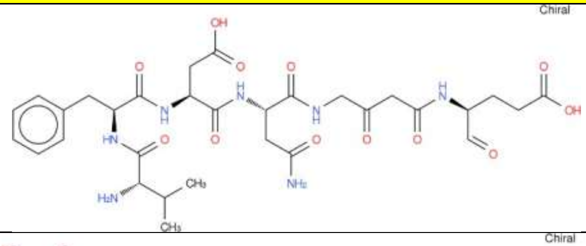
Cluster 2	Nrps	65406	Surfactin biosynthetic gene cluster (78% of genes show similarity)	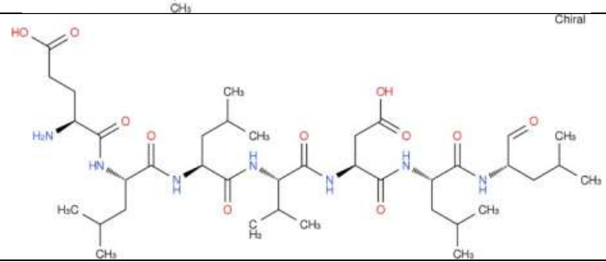
Cluster 3	Otherks	41244	Butirosin biosynthetic gene cluster (7% of genes show similarity)	-
Cluster 4	Terpene	20740	-	-
Cluster 5	Lantipeptide	28889	-	-
Cluster 6	Transatpks	85887	Macrolactin biosynthetic gene cluster (100% of genes show similarity)	-
Cluster 7	Transatpks-Nrps	102704	Bacillaene biosynthetic gene cluster (100% of genes show similarity)	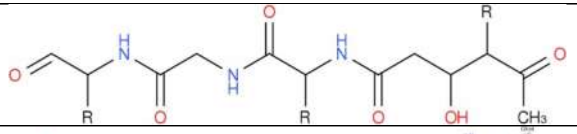
Cluster 8	Transatpks-Nrps	13828	Fengycin biosynthetic gene cluster (100% of genes show similarity)	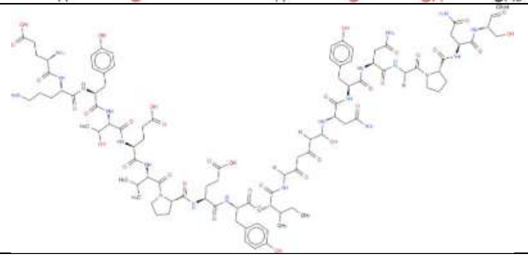

Table S1. Continued.

Strain clusters	Type	Length (bp)	Most similar known clusters	Predicted core clusters
**NJN-6**				
Cluster 9	Terpene	21883	-	-
Cluster 10	T3pks	41100	-	-
Cluster 11	Transatpks	100438		
Cluster 12	Bacteriocin-Nrps	66792	Bacillibactin biosynthetic gene cluster (100% of genes show similarity)	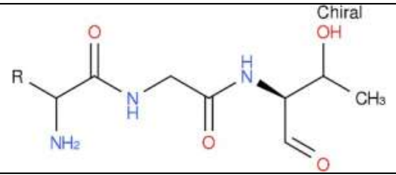
Cluster 13	Other	41418	Bacilysin biosynthetic gene cluster (100% of genes show similarity)	-

Table S1. Continued.

Strain clusters	Type	Length (bp)	Most similar known clusters	Predicted core clusters
**GR4-5**				
Cluster 1	Nrps	28112	Surfactin biosynthetic gene cluster (43% of genes show similarity)	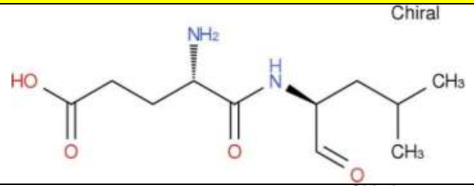
Cluster 2	Nrps	12207	Surfactin biosynthetic gene cluster (8% of genes show similarity)	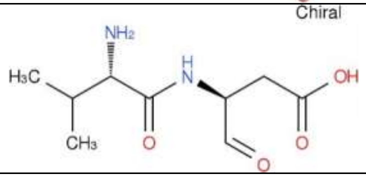
Cluster 3	Nrps	25694	Surfactin biosynthetic gene cluster (39% of genes show similarity)	-
Cluster 4	Ladderane	41121	-	-
Cluster 5	Nrps	27397	Bacitracin biosynthetic gene cluster (55% of genes show similarity)	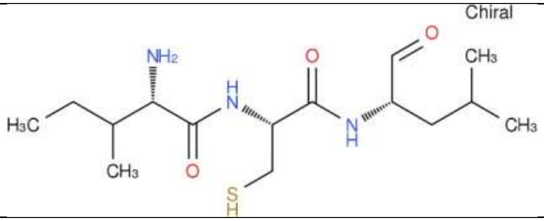
Cluster 6	Other	22533	Bacitracin biosynthetic gene cluster (33% of genes show similarity)	-
Cluster 7	Otherks	41244	Butirosin biosynthetic gene cluster (7% of genes show similarity)	-
Cluster 8	Terpene	20740	-	-
Cluster 9	Transatpks	85890	Macrolactin biosynthetic gene cluster (100% of genes show similarity)	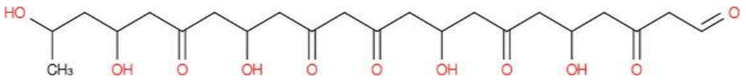
Cluster 10	Transatpks-Nrps	102683	Bacillaene biosynthetic gene cluster (100% of genes show similarity)	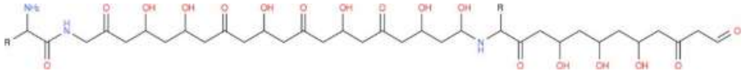

Table S1. Continued.

Strain clusters	Type	Length (bp)	Most similar known clusters	Predicted core clusters
**GR4-5**				
Cluster 11	Transatpks-Nrps	88084	Fengycin_biosynthetic_gene_cluster (80% of genes show similarity)	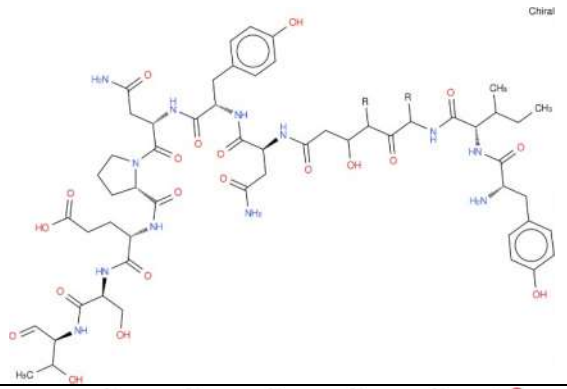
Cluster 12	Transatpks	24569	Difficidin biosynthetic gene cluster (26% of genes show similarity)	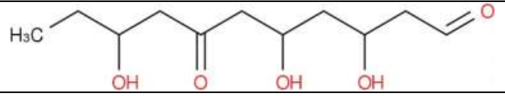
Cluster 13	Nrps	6577	Bacitracin biosynthetic gene cluster (33% of genes show similarity)	-
Cluster 14	Nrps	1880	-	-
Cluster 15	Nrps	15022	Fengycin biosynthetic gene cluster (20% of genes show similarity)	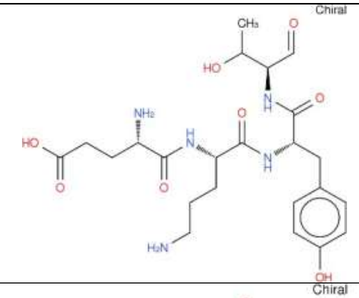
Cluster 16	Nrps	10690	Fengycin biosynthetic gene cluster (20% of genes show similarity)	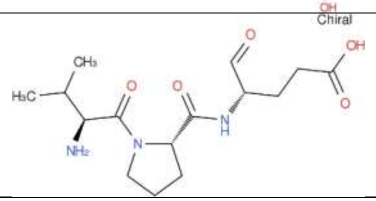

Table S1. Continued.

Strain clusters	Type	Length (bp)	Most similar known clusters	Predicted core clusters
**GR4-5**				
Cluster 17	Nrps	21836	Plipastatin biosynthetic gene cluster (30% of genes show similarity)	-
Cluster 18	Terpene	41883	-	-
Cluster 19	T3pks	41100	-	-
Cluster 20	Transatpks	47216	Difficidin biosynthetic gene cluster (53% of genes show similarity)	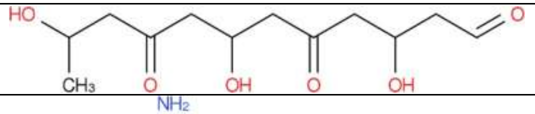
Cluster 21	Transatpks	28411	Difficidin biosynthetic gene cluster (46% of genes show similarity)	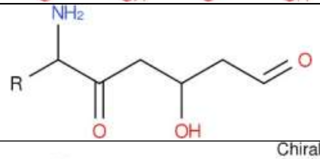
Cluster 22	Bacteriocin-Nrps	66792	Bacillibactin biosynthetic gene cluster (100% of genes show similarity)	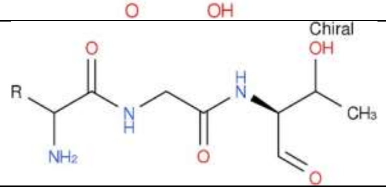
Cluster 23	Other	41418	Bacilysin biosynthetic gene cluster (100% of genes show similarity)	-
Cluster 24	Nrps	13235	Bacitracin biosynthetic gene cluster (33% of genes show similarity)	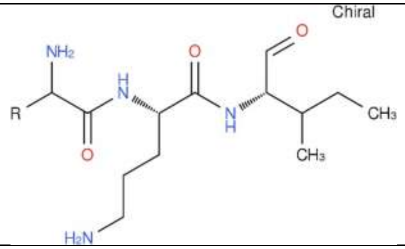
Cluster 25	Nrps	4143	-	-

Table S1. Continued.

Strain clusters	Type	Length (bp)	Most similar known clusters	Predicted core clusters
**M75**				
Cluster 1	Nrps	65407	Surfactin biosynthetic gene cluster (82% of genes show similarity)	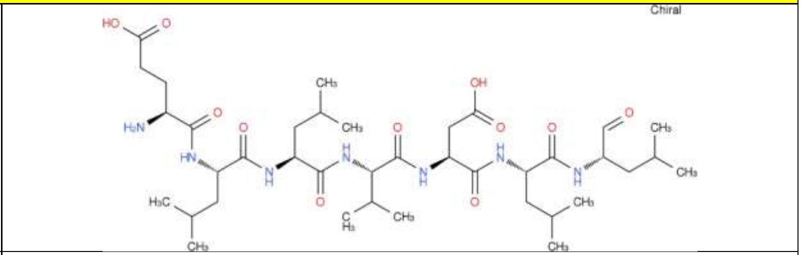
Cluster 2	Thiopeptide	27301	Kijanimicin biosynthetic gene cluster (4% of genes show similarity)	-
Cluster 3	Otherks	41244	Butirosin biosynthetic gene cluster (7% of genes show similarity)	-
Cluster 4	Terpene	20740	-	-
Cluster 5	Lantipeptide	28889	-	-
Cluster 6	Transatpks	85884	Macrolactin biosynthetic gene cluster (100% of genes show similarity)	-
Cluster 7	Transatpks-Nrps	102701	Bacillaene biosynthetic gene cluster (100% of genes show similarity)	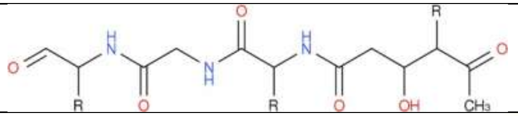
Cluster 8	Transatpks-Nrps	137829	Fengycin biosynthetic gene cluster (100% of genes show similarity)	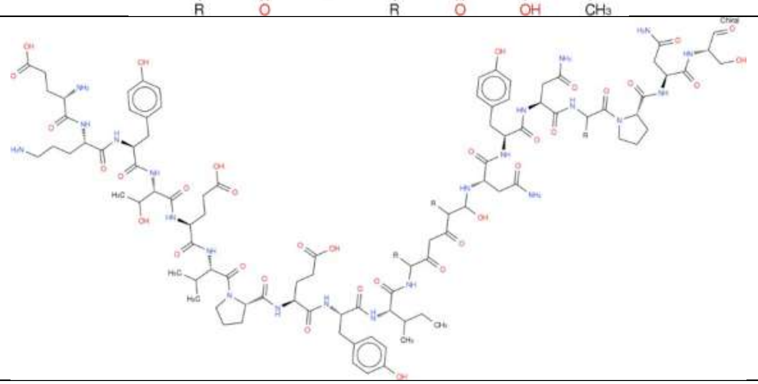
Cluster 9	Terpene	21883	-	-

Table S1. Continued.

Strain clusters	Type	Length (bp)	Most similar known clusters	Predicted core clusters
**M75**				
Cluster 10	T3pks	41100	-	-
Cluster 11	Transatpks	100447	Difficidin biosynthetic gene cluster (100% of genes show similarity)	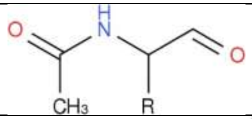
Cluster 12	Bacteriocin-Nrps	66791	Bacillibactin biosynthetic gene cluster (100% of genes show similarity)	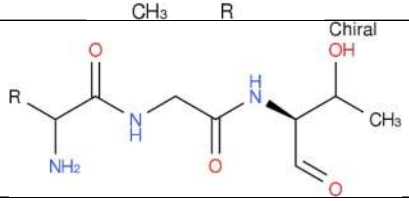
Cluster 13	Other	41418	Bacilysin biosynthetic gene cluster (100% of genes show similarity)	-

Table S1. Continued.

Strain clusters	Type	Length (bp)	Most similar known clusters	Predicted core clusters
**GB1**				
Cluster 1	Phosphonate	40884	-	-
Cluster 2	Nrps	65407	Surfactin biosynthetic gene cluster (82% of genes show similarity)	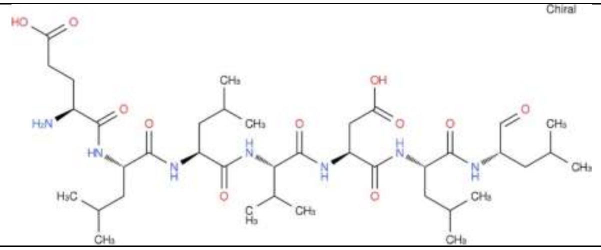
Cluster 3	Lantipeptide	23984	Mersacidin biosynthetic gene cluster (100% of genes show similarity)	-
Cluster 4	Other	41418	Bacilysin biosynthetic gene cluster (100% of genes show similarity)	-
Cluster 5	Nrps	68420	-	-
Cluster 6	Bacteriocin-Nrps	66793	Bacillibactin biosynthetic gene cluster (100% of genes show similarity)	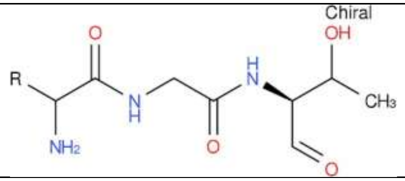
Cluster 7	Transatpks	100453	Difficidin biosynthetic gene cluster (100% of genes show similarity)	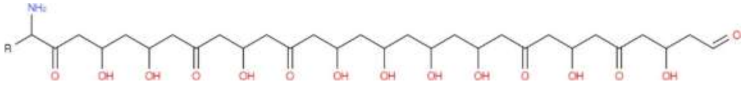
Cluster 8	T3pks	24956	-	-
Cluster 9	Terpene	21883	-	-
Cluster 10	Transatpks-Nrps	137832	Fengycin biosynthetic gene cluster (100% of genes show similarity)	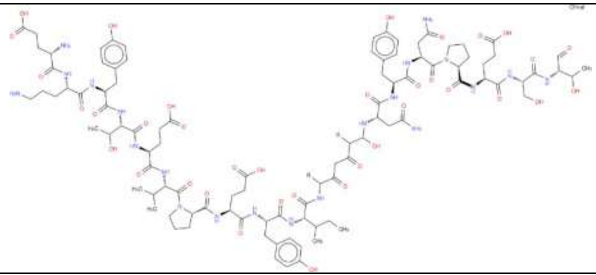

Table S1. Continued.

Strain clusters	Type	Length (bp)	Most similar known clusters	Predicted core clusters
**GB1**				
Cluster 11	Transatpks-Nrps	102680	Bacillaene biosynthetic gene cluster (100% of genes show similarity)	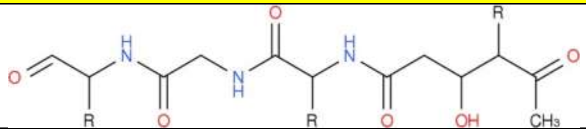
Cluster 12	Transatpks	85902	Macrolactin biosynthetic gene cluster (100% of genes show similarity)	-
Cluster 13	Terpene	20740	-	-
Cluster 14	Otherks	41244	Butirosin biosynthetic gene cluster (7% of genes show similarity)	-

Table S1. Continued.

Strain clusters	Type	Length (bp)	Most similar known clusters	Predicted core clusters
**JTYP2**				
Cluster 1	Nrps	65407	Surfactin biosynthetic gene cluster (82% of genes show similarity)	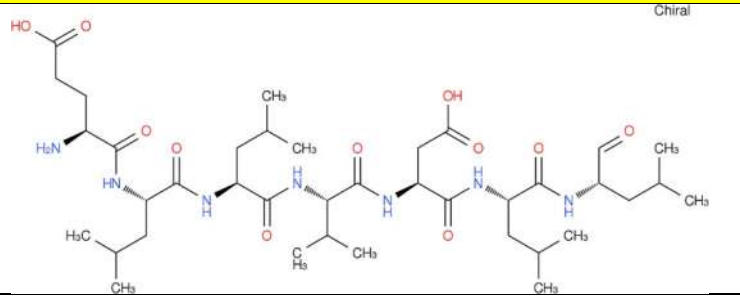
Cluster 2	Otherks	41244	Butirosin biosynthetic gene cluster (7% of genes show similarity)	-
Cluster 3	Terpene	20740	-	-
Cluster 4	Lantipeptide	28888	-	-
Cluster 5	Transatpks	85904	Macrolactin biosynthetic gene cluster (100% of genes show similarity)	-
Cluster 6	Transatpks-Nrps	102674	Bacillaene biosynthetic gene cluster (100% of genes show similarity)	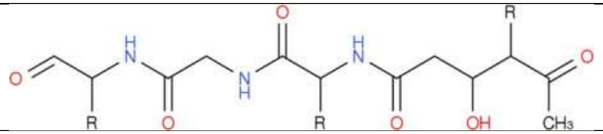
Cluster 7	Transatpks-Nrps	137801	Fengycin biosynthetic gene cluster (100% of genes show similarity)	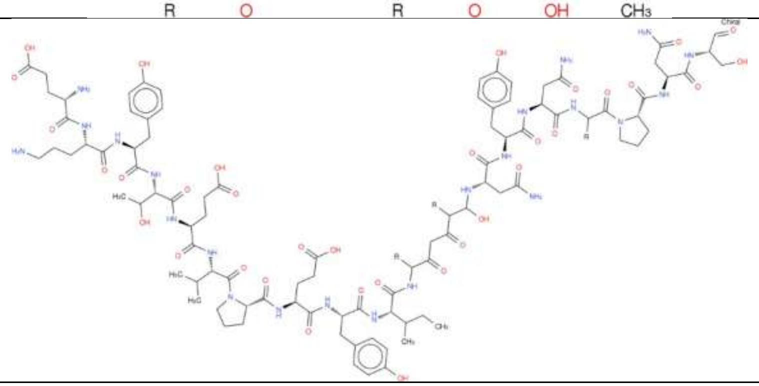
Cluster 8	Terpene	21883	-	-
Cluster 9	T3pks	41100	-	-

Table S1. Continued.

Strain clusters	Type	Length (bp)	Most similar known clusters	Predicted core clusters
**JTYP2**				
Cluster 10	Transatpks	100453	Difficidin biosynthetic gene cluster (100% of genes show similarity)	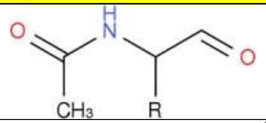
Cluster 11	Bacteriocin-Nrps	66792	Bacillibactin biosynthetic gene cluster (100% of genes show similarity)	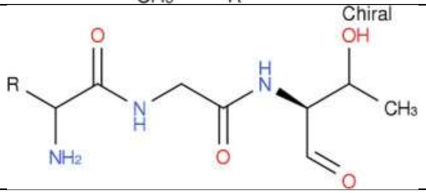
Cluster 12	Other	41418	Bacilysin biosynthetic gene cluster (100% of genes show similarity)	-

Table S1. Continued.

Strain clusters	Type	Length (bp)	Most similar known clusters	Predicted core clusters
**HJ18-4**				
Cluster 1	Transatpks-Nrps	77723	Rhizocticin biosynthetic gene cluster (22% of genes show similarity)	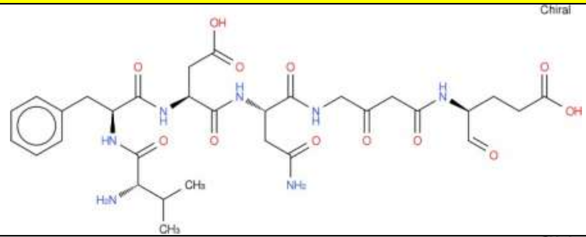
Cluster 2	Nrps	65434	Surfactin biosynthetic gene cluster (78% of genes show similarity)	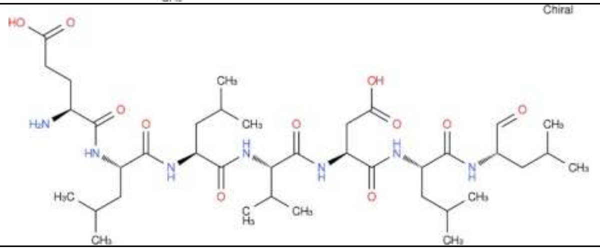
Cluster 3	Otherks	41244	Butirosin biosynthetic gene cluster (7% of genes show similarity)	-
Cluster 4	Terpene	20740	-	-
Cluster 5	Lantipeptide	28889	-	-
Cluster 6	Transatpks	85890	Macrolactin biosynthetic gene cluster (100% of genes show similarity)	-
Cluster 7	Transatpks-Nrps	102689	Bacillaene biosynthetic gene cluster (100% of genes show similarity)	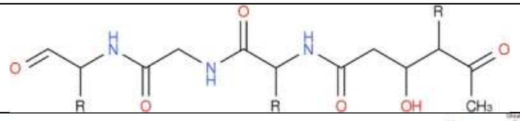
Cluster 8	Transatpks-Nrps	137838	Fengycin biosynthetic gene cluster (100% of genes show similarity)	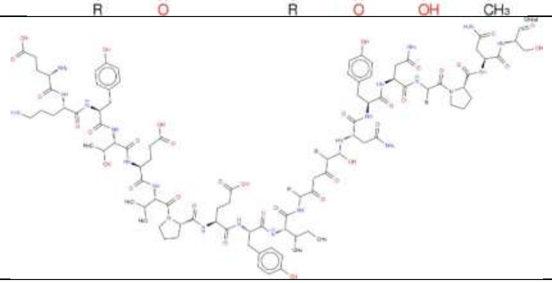

Table S1. Continued.

Strain clusters	Type	Length (bp)	Most similar known clusters	Predicted core clusters
**HJ18-4**				
Cluster 9	Terpene	21883	-	-
Cluster 10	T3pks	41100	-	-
Cluster 11	Transatpks	100435	Difficidin biosynthetic gene cluster (100% of genes show similarity)	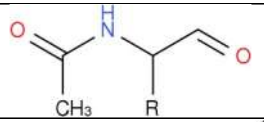
Cluster 12	Bacteriocin-Nrps	66793	Bacillibactin biosynthetic gene cluster (100% of genes show similarity)	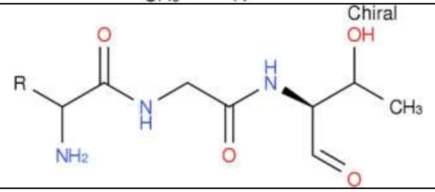
Cluster 13	Other	41418	Bacilysin biosynthetic gene cluster (100% of genes show similarity)	-

Table S1. Continued.

Strain clusters	Type	Length (bp)	Most similar known clusters	Predicted core clusters
**CFSAN034339**				
Cluster 1	Bacteriocin-Nrps	66055	Bacillibactin biosynthetic gene cluster (100% of genes show similarity)	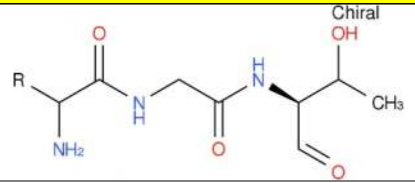
Cluster 2	Transatpks	23024	Difficidin biosynthetic gene cluster (26% of genes show similarity)	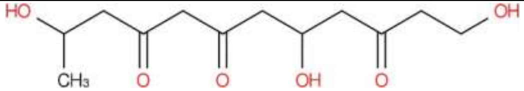
Cluster 3	Nrps	13961	Fengycin biosynthetic gene cluster (20% of genes show similarity)	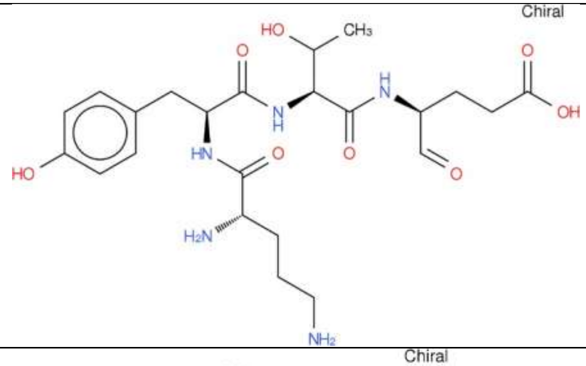
Cluster 4	Nrps	10021	Surfactin biosynthetic gene cluster (8% of genes show similarity)	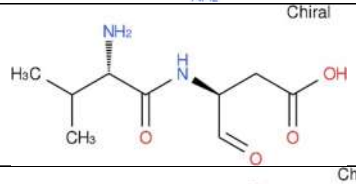
Cluster 5	Nrps	9308	Fengycin biosynthetic gene cluster (13% of genes show similarity)	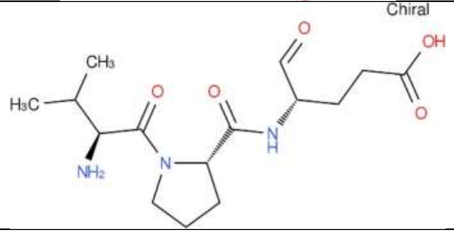
Cluster 6	Otherks	41244	Butirosin biosynthetic gene cluster (7% of genes show similarity)	-

Table S1. Continued.

Strain clusters	Type	Length (bp)	Most similar known clusters	Predicted core clusters
**CFSAN034339**				
Cluster 7	Terpene	20740	-	-
Cluster 8	Transatpks	85892	Macrolactin biosynthetic gene cluster (100% of genes show similarity)	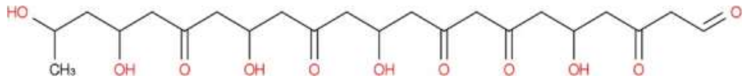
Cluster 9	Transatpks-Nrps	102671	Bacillaene biosynthetic gene cluster (100% of genes show similarity)	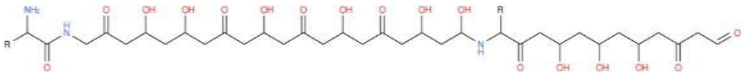
Cluster 10	Transatpks-Nrps	87722	Fengycin biosynthetic gene cluster (80% of genes show similarity)	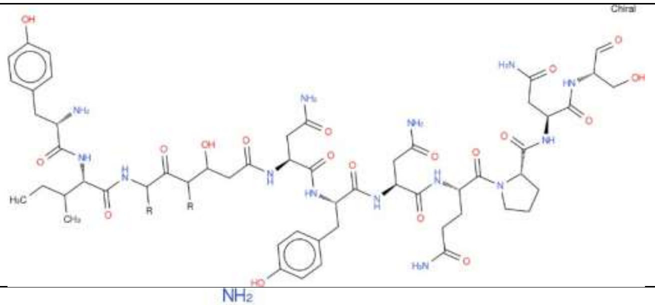
Cluster 11	Transatpks	28377	Difficidin biosynthetic gene cluster (46% of genes show similarity)	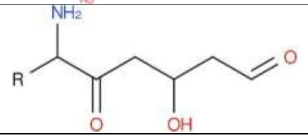
Cluster 12	Other	41418	Bacilysin biosynthetic gene cluster (100% of genes show similarity)	-
Cluster 13	Nrps	39773	-	-
Cluster 14	Lantipeptide	27057	Subtilin biosynthetic gene cluster (100% of genes show similarity)	-
Cluster 15	T3pks	41100	-	-
Cluster 16	Terpene	21883	-	-
Cluster 17	Nrps	23565	Plipastatin biosynthetic gene cluster (38% of genes show similarity)	-
Cluster 18	Nrps	25682	Surfactin biosynthetic gene cluster (39% of genes show similarity)	-

Table S1. Continued.

Strain clusters	Type	Length (bp)	Most similar known clusters	Predicted core clusters
**CFSAN034339**				
Cluster 19	Transatpks-Nrps	77735	Rhizocticin biosynthetic gene cluster (22% of genes show similarity)	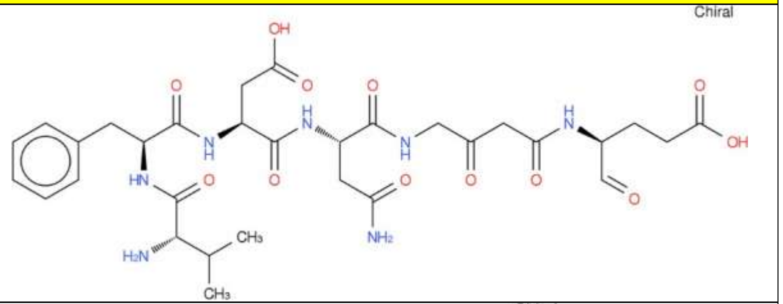
Cluster 20	Nrps	28212	Surfactin biosynthetic gene cluster (47% of genes show similarity)	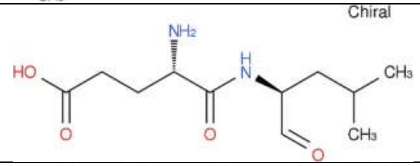
Cluster 21	Transatpks	46427	Difficidin biosynthetic gene cluster (53% of genes show similarity)	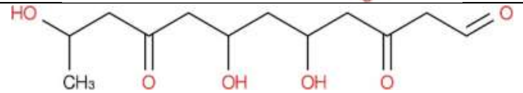

Table S1. Continued.

Strain clusters	Type	Length (bp)	Most similar known clusters	Predicted core clusters
**CH13**				
Cluster 1	Nrps	65632	Surfactin biosynthetic gene cluster (91% of genes show similarity)	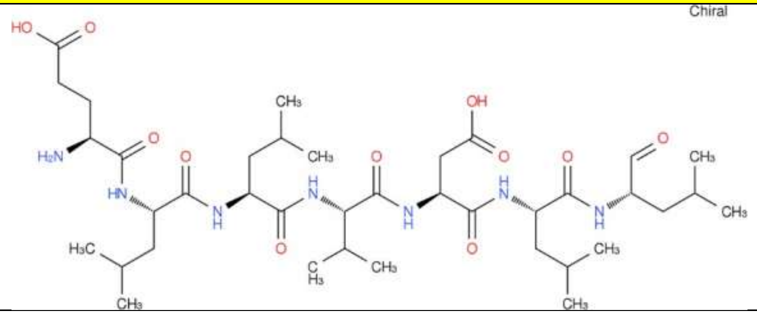
Cluster 2	Otherks	41244	Butirosin biosynthetic gene cluster (7% of genes show similarity)	-
Cluster 3	Terpene	20740	-	-
Cluster 4	Transatpks	85899	Macrolactin biosynthetic gene cluster (100% of genes show similarity)	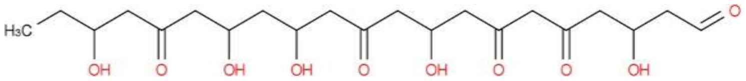
Cluster 5	Transatpks-Nrps	102683	Bacillaene biosynthetic gene cluster (100% of genes show similarity)	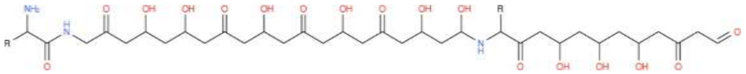
Cluster 6	Transatpks-Nrps	114360	Fengycin biosynthetic gene cluster (93% of genes show similarity)	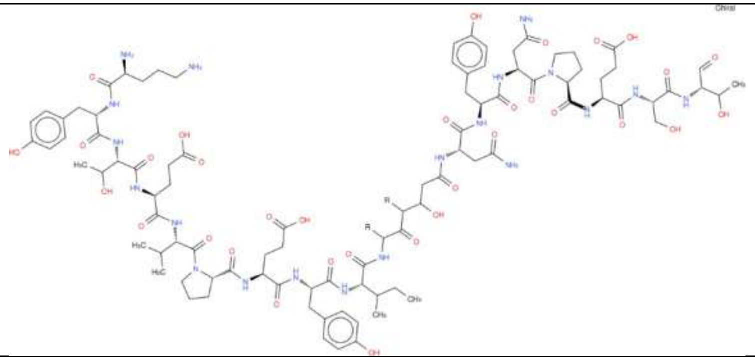
Cluster 7	Nrps	22514	Plipastatin_biosynthetic_gene_cluster (30% of genes show similarity)	-
Cluster 8	Terpene	21883	-	-

Table S1. Continued.

Strain clusters	Type	Length (bp)	Most similar known clusters	Predicted core clusters
**CH13**				
Cluster 9	T3pks	41100	-	-
Cluster 10	Transatpks	71039	Difficidin biosynthetic gene cluster (66% of genes show similarity)	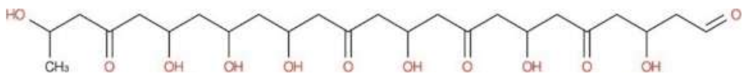
Cluster 11	Transatpks	28584	Difficidin biosynthetic gene cluster (46% of genes show similarity)	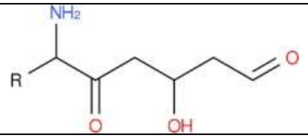
Cluster 12	Nrps	30002	-	-
Cluster 13	Nrps	22198	-	-
Cluster 14	Bacteriocin-Nrps	66791	Bacillibactin biosynthetic gene cluster (100% of genes show similarity)	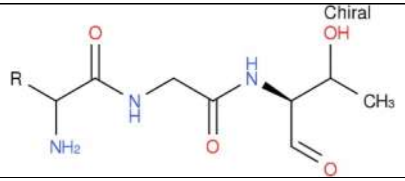
Cluster 15	Other	41418	Bacilysin biosynthetic gene cluster (100% of genes show similarity)	-

Table S1. Continued.

Strain clusters	Type	Length (bp)	Most similar known clusters	Predicted core clusters
**GH1-13**				
Cluster 1	Transatpks-Nrps	77727	Rhizocticin biosynthetic gene cluster (22% of genes show similarity)	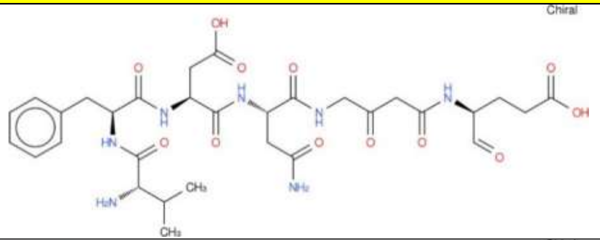
Cluster 2	Nrps	65410	Surfactin biosynthetic gene cluster (82% of genes show similarity)	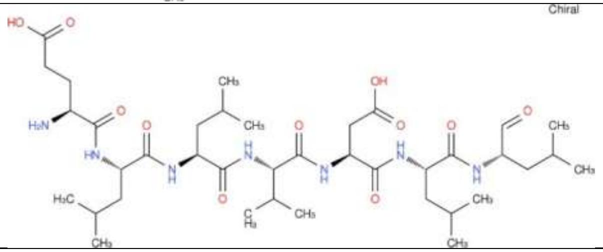
Cluster 3	Otherks	41244	Butirosin biosynthetic gene cluster (7% of genes show similarity)	-
Cluster 4	Terpene	20740	-	-
Cluster 5	Transatpks	85884	Macrolactin biosynthetic gene cluster (100% of genes show similarity)	-
Cluster 6	Transatpks-Nrps	102701	Bacillaene biosynthetic gene cluster (100% of genes show similarity)	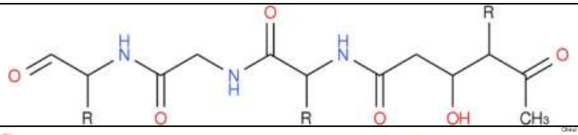
Cluster 7	Transatpks-Nrps	137853	Fengycin biosynthetic gene cluster (100% of genes show similarity)	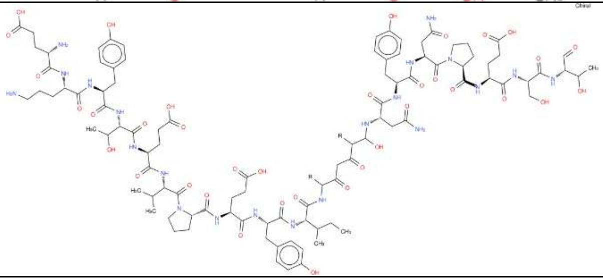

Table S1. Continued.

Strain clusters	Type	Length (bp)	Most similar known clusters	Predicted core clusters
**GH1-13**				
Cluster 8	Terpene	21883	-	-
Cluster 9	T3pks	41100	-	-
Cluster 10	Transatpks	100447	Difficidin biosynthetic gene cluster (100% of genes show similarity)	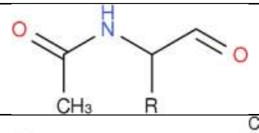
Cluster 11	Bacteriocin-Nrps	66793	Bacillibactin biosynthetic gene cluster (100% of genes show similarity)	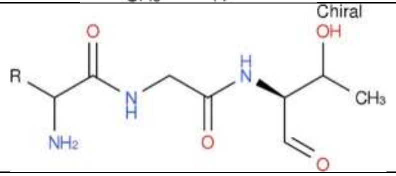
Cluster 12	Other	41418	Bacilysin biosynthetic gene cluster (100% of genes show similarity)	-
Cluster 13	Lantipeptide	23983	Mersacidin biosynthetic gene cluster (100% of genes show similarity)	-

Table S1. Continued.

Strain clusters	Type	Length (bp)	Most similar known clusters	Predicted core clusters
**AH159-1**				
Cluster 1	Nrps	9337	Fengycin biosynthetic gene cluster (13% of genes show similarity)	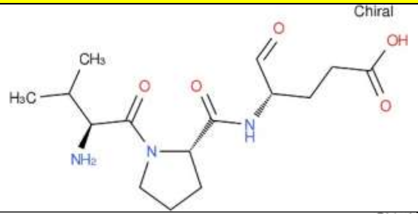
Cluster 2	Nrps	29784	Fengycin biosynthetic gene cluster (73% of genes show similarity)	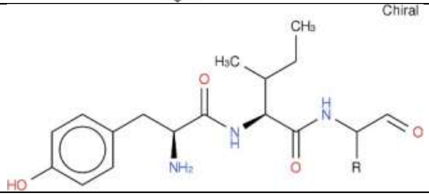
Cluster 3	Transatpks-Nrps	41926	Bacillomycin biosynthetic gene cluster (60% of genes show similarity)	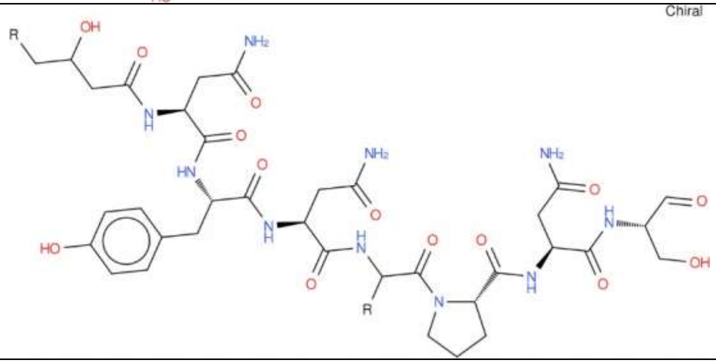
Cluster 4	Transatpks	10567	Bacillaene biosynthetic gene cluster (14% of genes show similarity)	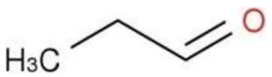
Cluster 5	Transatpks-Nrps	21950	Bacillaene biosynthetic gene cluster (35% of genes show similarity)	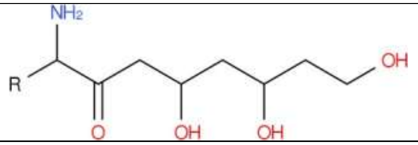
Cluster 6	Transatpks-Nrps	47403	Bacillaene biosynthetic gene cluster (85% of genes show similarity)	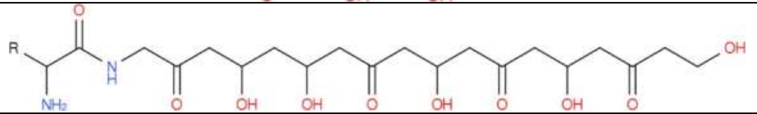

Table S1. Continued.

Strain clusters	Type	Length (bp)	Most similar known clusters	Predicted core clusters
**AH159-1**				
Cluster 7	Transatpks	71015	Macrolactin biosynthetic gene cluster (90% of genes show similarity)	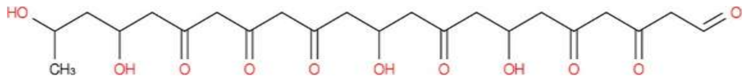
Cluster 8	Terpene	15536	-	-
Cluster 9	Nrps	2846	-	-
Cluster 10	Terpene	14261	-	-
Cluster 11	T3pks	15359	-	-
Cluster 12	Transatpks	30066	Difficidin biosynthetic gene cluster (33% of genes show similarity)	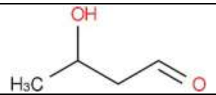
Cluster 13	Transatpks	15801	Difficidin biosynthetic gene cluster (26% of genes show similarity)	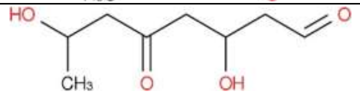
Cluster 14	Transatpks	22943	Difficidin biosynthetic gene cluster (26% of genes show similarity)	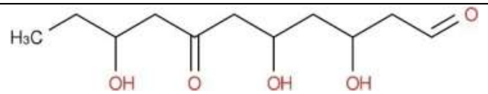
Cluster 15	Transatpks	14496	Difficidin biosynthetic gene cluster (46% of genes show similarity)	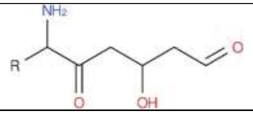
Cluster 16	Lantipeptide	2029	-	-
Cluster 17	Other	24406	Bacilysin biosynthetic gene cluster (100% of genes show similarity)	-
Cluster 18	Nrps	7132	Surfactin biosynthetic gene cluster (21% of genes show similarity)	-
Cluster 19	Nrps	9506	Surfactin biosynthetic gene cluster (8% of genes show similarity)	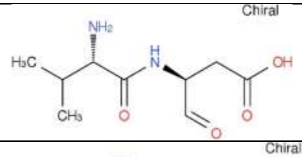
Cluster 20	Nrps	27847	Surfactin biosynthetic gene cluster (47% of genes show similarity)	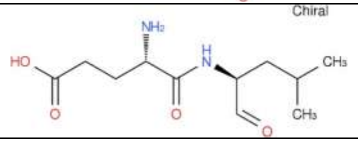

Table S1. Continued.

Strain clusters	Type	Length (bp)	Most similar known clusters	Predicted core clusters
**AH159-1**				
Cluster 21	Bacteriocin	8561	Amylocyclicin biosynthetic gene cluster (100% of genes show similarity)	-
Cluster 22	Nrps	30192	Bacillibactin biosynthetic gene cluster (100% of genes show similarity)	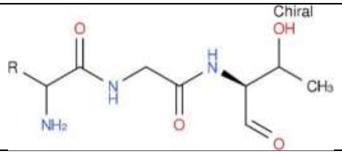
Cluster 23	Ladderane	41121	-	-
Cluster 24	Nrps	12665	Fengycin biosynthetic gene cluster (20% of genes show similarity)	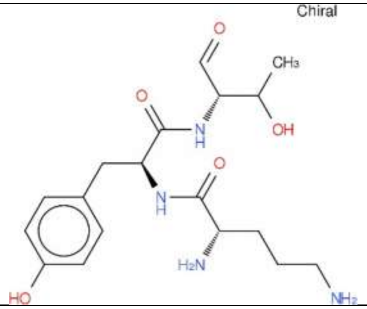

Table S1. Continued.

Strain clusters	Type	Length (bp)	Most similar known clusters	Predicted core clusters
**2A-2B**				
Cluster 1	Transatpks	85899	Macrolactin biosynthetic gene cluster (100% of genes show similarity)	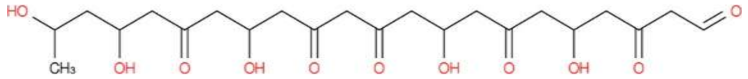
Cluster 2	Transatpks-Nrps	102686	Bacillaene biosynthetic gene cluster (100% of genes show similarity)	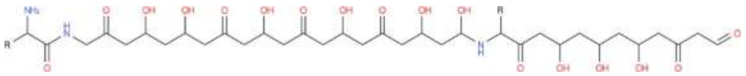
Cluster 3	Transatpks-Nrps	87921	Fengycin biosynthetic gene cluster (80% of genes show similarity)	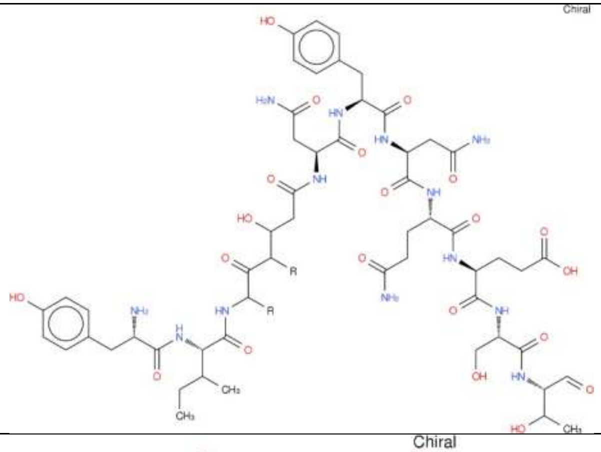
Cluster 4	Bacteriocin-Nrps	66791	Bacillibactin biosynthetic gene cluster (100% of genes show similarity)	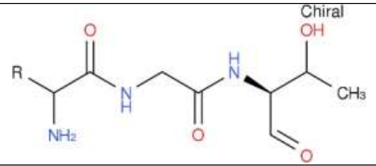
Cluster 5	Nrps	35335	-	-
Cluster 6	Terpene	21883	-	-
Cluster 7	T3pks	41100	-	-
Cluster 8	Transatpks	45760	Difficidin biosynthetic gene cluster (53% of genes show similarity)	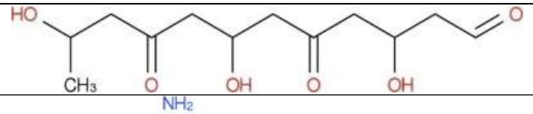
Cluster 9	Transatpks	28251	Difficidin biosynthetic gene cluster (46% of genes show similarity)	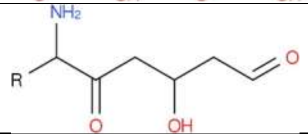

Table S1. Continued.

Strain clusters	Type	Length (bp)	Most similar known clusters	Predicted core clusters
**2A-2B**				
Cluster 10	Terpene	20740	-	-
Cluster 11	Otherks	41244	Butirosin biosynthetic gene cluster (7% of genes show similarity)	-
Cluster 12	Other	41418	Bacilysin biosynthetic gene cluster (100% of genes show similarity)	-
Cluster 13	Nrps	24050	-	-
Cluster 14	Nrps	28056	Surfactin biosynthetic gene cluster (47% of genes show similarity)	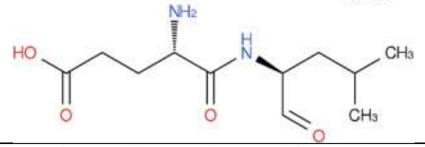
Cluster 15	Transatpks	22899	Difficidin biosynthetic gene cluster (26% of genes show similarity)	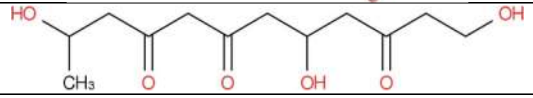
Cluster 16	Nrps	15095	Surfactin biosynthetic gene cluster (39% of genes show similarity)	-
Cluster 17	Nrps	12809	Fengycin biosynthetic gene cluster (26% of genes show similarity)	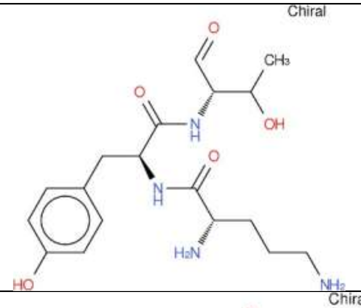
Cluster 18	Nrps	9454	Fengycin biosynthetic gene cluster (20% of genes show similarity)	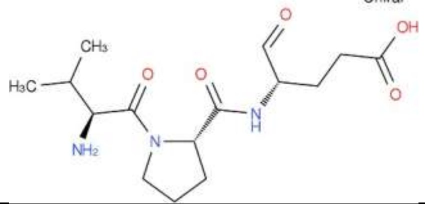

Table S1. Continued.

Strain clusters	Type	Length (bp)	Most similar known clusters	Predicted core clusters
**2A-2B**				
Cluster 19	Nrps	8988	Surfactin biosynthetic gene cluster (8% of genes show similarity)	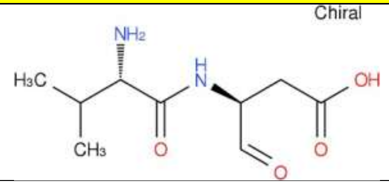
Cluster 20	Nrps	7816	-	-
Cluster 21	Nrps	1482	-	-
Cluster 22	Nrps	1413	-	-

Table S1. Continued.

Strain clusters	Type	Length (bp)	Most similar known clusters	Predicted core clusters
**ARYD01000001**				
Cluster 1	Nrps	23248	-	-
Cluster 2	Other	41418	Bacilysin biosynthetic gene cluster (100% of genes show similarity)	-
Cluster 3	Otherks	41244	Butirosin biosynthetic gene cluster (7% of genes show similarity)	-
Cluster 4	Terpene	20740	-	-
Cluster 5	Transatpks-Nrps	99966	Bacillaene biosynthetic gene cluster (100% of genes show similarity)	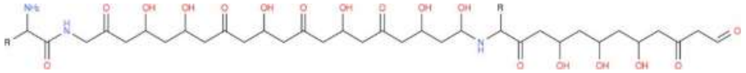
Cluster 6	Transatpks	45842	Difficidin biosynthetic gene cluster (53% of genes show similarity)	-
Cluster 7	T3pks	41100	-	-
Cluster 8	Transatpks	85902	Macrolactin biosynthetic gene cluster (100% of genes show similarity)	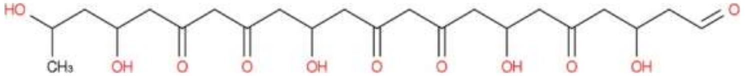
Cluster 9	Bacteriocin-Nrps	65849	Bacillibactin biosynthetic gene cluster (100% of genes show similarity)	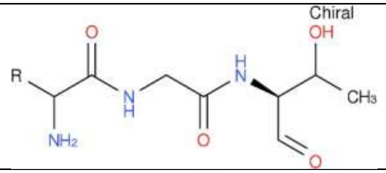
Cluster 10	Transatpks-Nrps	88037	Fengycin biosynthetic gene cluster (86% of genes show similarity)	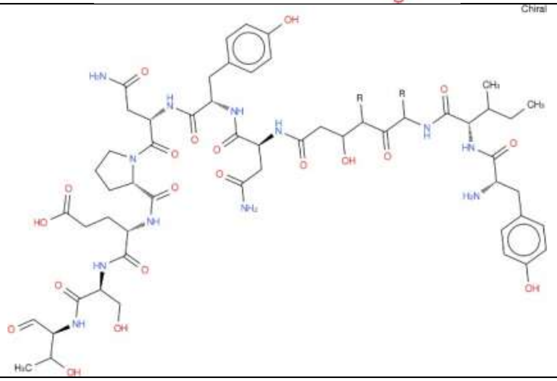

Table S1. Continued.

Strain clusters	Type	Length (bp)	Most similar known clusters	Predicted core clusters
**ARYD01000001**				
Cluster 11	Transatpks	28283	Difficidin biosynthetic gene cluster (46% of genes show similarity)	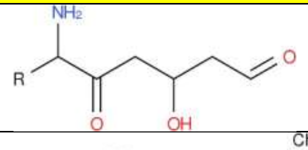
Cluster 12	Nrps	27868	Surfactin biosynthetic gene cluster (47% of genes show similarity)	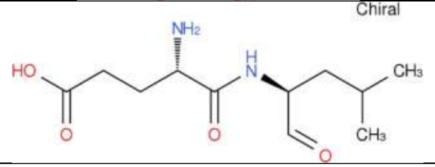
Cluster 13	Phosphonate	40890	-	-
Cluster 14	Terpene	21883	-	-
Cluster 15	Nrps	21780	Plipastatin biosynthetic gene cluster (30% of genes show similarity)	-
Cluster 16	Transatpks	23101	Difficidin biosynthetic gene cluster (26% of genes show similarity)	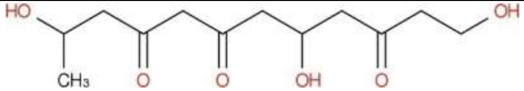
Cluster 17	Nrps	15228	Surfactin biosynthetic gene cluster (39% of genes show similarity)	-
Cluster 18	Nrps	13069	Fengycin biosynthetic gene cluster (20% of genes show similarity)	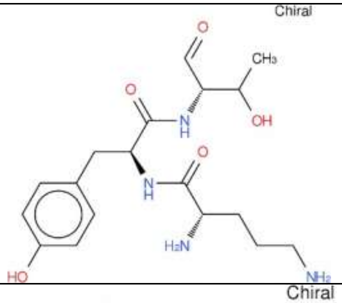
Cluster 19	Nrps	9721	Surfactin biosynthetic gene cluster (8% of genes show similarity)	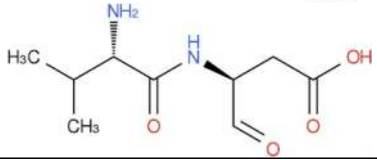

Table S1. Continued.

Strain clusters	Type	Length (bp)	Most similar known clusters	Predicted core clusters
**ARYD01000001**				
Cluster 20	Nrps	9091	Fengycin biosynthetic gene cluster (13% of genes show similarity)	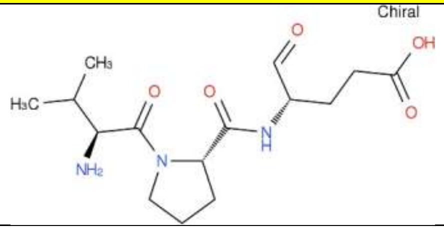
Cluster 21	Nrps	6895	-	-
Cluster 22	Nrps	6499	-	-

Table S1. Continued.

Strain clusters	Type	Length (bp)	Most similar known clusters	Predicted core clusters
**9D-6**				
Cluster 1	Other	41418	Bacilysin biosynthetic gene cluster (100% of genes show similarity)	-
Cluster 2	Nrps	65407	Surfactin biosynthetic gene cluster (91% of genes show similarity)	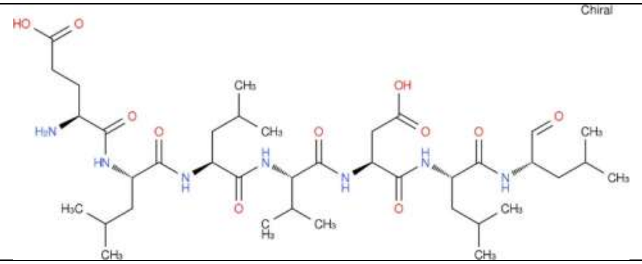
Cluster 3	Ladderane	41121	-	-
Cluster 4	Otherks	41244	Butirosin biosynthetic gene cluster (7% of genes show similarity)	-
Cluster 5	Terpene	20740	-	-
Cluster 6	Lantipeptide	28888	-	-
Cluster 7	Transatpks	85893	Macrolactin biosynthetic gene cluster (100% of genes show similarity)	-
Cluster 8	Transatpks-Nrps	102686	Bacillaene biosynthetic gene cluster (100% of genes show similarity)	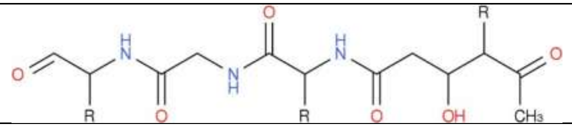
Cluster 9	Bacteriocin-Transatpks-Nrps	125093	Fengycin biosynthetic gene cluster (93% of genes show similarity)	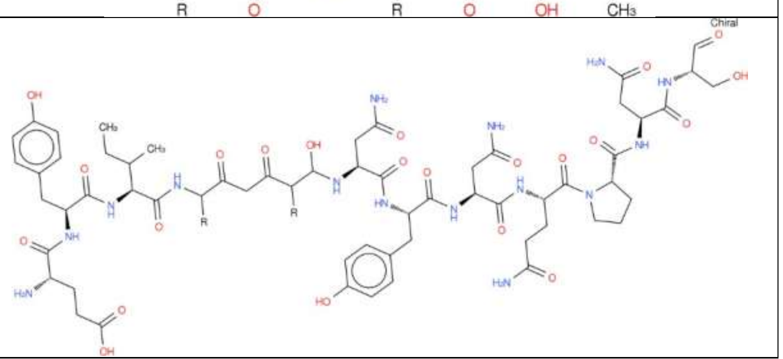

Table S1. Continued.

Strain clusters	Type	Length (bp)	Most similar known clusters	Predicted core clusters
**9D-6**				
Cluster 10	Terpene	21883	-	-
Cluster 11	T3pks	41100	-	-
Cluster 12	Transatpks	100453	Difficidin biosynthetic gene cluster (100% of genes show similarity)	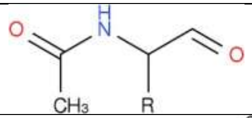
Cluster 13	Bacteriocin-Nrps	66810	Bacillibactin biosynthetic gene cluster (100% of genes show similarity)	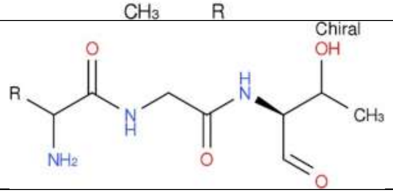

Table S2. List and description of *B. velezensis* strains used in the study.

Strain	Genome size (Mb)	Plasmid	Description	GenBank Accession
FZB42	3.91859	0	A naturally occurring isolate, distinguished from the model organism *Bacillus subtilis* 168 by its abilities to stimulate plant growth and suppress plant pathogens	CP000560.1
CAU B946	4.01986	0	Isolated from the rice rhizosphere, identified by 16S rRNA gene and gyrA gene sequencing and by physiological and biochemical analysis as being *Bacillus amyloliquefaciens* subsp. *plantarum*	HE617159.1
YAU B9601-Y2	4.24277	0	Isolated from the wheat rhizosphere in North China, identified as being *B. amyloliquefaciens* subsp. *plantarum*. The strain suppresses a broad spectrum of pathogenic fungi; promotes growth and rooting of crops and vegetables; improves the drought tolerance of wheat, corn, and broad bean; and reduces the number of nematodes at tomato and tobacco roots.	HE774679.1
AS43.3	3.96137	0	Originally isolated from a wheat head and was subsequently shown to be effective in reducing FHB incidence and severity in wheat; An antagonist of *Gibberella zeae*, the primary causal agent of Fusarium head blight (FHB) in the United States.	CP003838.1
UCMB5036	3.91032	0	A plant growth-promoting bacterium, originally isolated from cotton (Ukraine)	HF563562.1
UCMB5033	4.07117	0	Ability to promote host plant growth through production of stimulating compounds and suppression of soil borne pathogens by synthesizing antibacterial and antifungal metabolites or priming plant defence as induced systemic resistance in oilseed rape (*Brassica napus*)	HG328253.1
UCMB5113	3.88953	0	Isolated from soil in the Karpaty mountains of Ukraine; promote growth of both underground and aboveground tissues of different plants; restrict the growth of several fungal pathogens on oilseed rape such as *Alternaria brassicae*, *Botrytis cinerea*, *Leptosphaeria maculans* and *Verticillium longisporum*	HG328254.1
NAU-B3	4.20461	1	Isolated from the wheat rhizosphere in Jiangsu province, East China	HG514499.1
TrigoCor1448	3.9579	0	Isolated from the surface of a wheat spike (North America); A promising FHB biological control agent isolated from the rhizosphere of a wheat plant	CP007244.1
D2-2	3.92183	0	Isolated from South Korea	CP014990.1
SQR9	4.11702	0	Isolated from the plant rhizosphere and is able to reduce attack by *Fusarium oxysporum* f. sp. *cucumerinum* through efficient root colonization followed by production of antifungal metabolites; Products derived from SQR9 are also widely used in agriculture in China under the BIO™ trademark. An ideal PGPR strain for exploring rhizoshphere plant-microbe interactions.	CP006890.1
JS25R	4.01444	1	Isolated from wheat head in China; Biocontrol activity against *Fusarium verticilliodes* and *Alternaria alternata*	CP009679.1
NJN-6	4.05255	1	Isolated from the rhizosphere soil of healthy banana plants, acts as an efficient antagonist against *F. oxysporum* f. sp. *cubense* by producing several antibiotics	CP007165.1
JJ-D34	4.10595	0	Isolated from fermented soybean product (South Korea) with good proteolytic and antipathogenic activities	CP011346.1

Table S2. Continued.

Strain	Genome size (Mb)	Plasmid	Description	GenBank Accession
YJ11-1-4	4.00664	0	Isolated from Doenjang, Korean traditional fermented soybean paste (South Korea)	CP011347.1
G341	4.00975	0	Isolated from 4-year-old roots of Korean ginseng with rot; Antibacterial and antifungal activities against diverse plant pathogens owing to their broad inhibitory spectra	CP011686.1
B25	3.86276	1	Isolated in Switzerland from the inner wood tissues of a decaying *Platanus acerifolia* tree; Beneficial effects on their hosts, such as growth promotion and health enhancement.	LN999829.1
CC09	4.16715	0	Isolated from *Cinnamomum camphora* leaf tissue, which can be used to improve plant growth and prevent fungal diseases in plants caused by *Glomerella glycines*, *Rhizoctonia solani*, and *Alternaria alternata* by producing bioactive compounds	CP015443.1
S3-1	3.92977	0	Isolated from the rhizosphere soil of cucumber in Tangshan, China; A broad-spectrum resistance to plant pathogens	CP016371.1
M75	4.00745	0	Isolated from cotton waste used for mushroom cultivation in Suwon, Korea; Strong antagonistic activity of the M75 strain towards several plant pathogenic fungi, including *Rhizoctonia solani*	CP015911.1
sx01604	3.92652	0	Isolated from soil in Beijing (China)	CP018007.1
SYBC H47	3.88443	0	Isolated from honey (China); Antifungal activity against *Aspergillus niger*, *Mucor racemosus*, *Fusarium oxysporum*, *Penicillium citrinum*, and *Candida albicans*; Inhibit the germination of conidia and the growth of mycelia from *B. dothidea* as a potential biocontrol agent against the gummosis disease	CP017747.1
9912D	4.24158	1	Isolated from sediment sample from the Liaodong Bay of the Bohai Sea (China); Approved as the first biopesticide formulation by the Ministry of Agriculture of the People's Republic of China; Highly efficient in preventing plant diseases such as cucumber and tomato grey mould, cotton wilt and apple rot disease	CP017775.1
GH1-13	4.14361	1	Isolated from rice paddy soil in Korea; Promote plant growth and have strong antagonistic activities against pathogens; *Fusarium fujikuroi*, *Rhizoctonia solani*	CP019040.1
JTYP2	3.92979	0	Isolated from the leaves of *Echeveria laui* in Qingzhou, China, and may control some of the fungal pathogens of the plant including *Fusarium inflexum*	CP020375.1
9D-6	3.96373	0	Isolated from rhizosphere soil of potato in Canada	CP020805.1
CBMB205	3.92979	0	A methanol-utilizing, plant-growth-promoting bacterium isolated from the rhizosphere soil of traditionally cultivated, field-grown rice (Cheongwon, Korea);	CP011937.1
ZL918	3.92271	0	Isolated from infected bulbs (*Sagittaria sagittifolia*) in Hubei (China)	CP021338.1
GB1	4.01448	0	Isolated from aging cucumber stems, exhibited a strong antagonistic activity	LHCG00000000.1
M27	3.86096	0	Isolated from cotton-waste compost (Korea) that was used in the cultivation of oyster mushrooms (*Pleurotus ostreatus*); A broad spectrum of pathogenic fungi, including *Fusarium oxysporum*, *Phytophthora capsici*, *Rhizoctonia solani*, *Sclerotinia sclerotiorum*, *Trichoderma harzianum*, *Trichoderma koningii*, and *Trichoderma viridescens*	AMPK00000000.1

Table S2. Continued.

Strain	Genome size (Mb)	Plasmid	Description	GenBank Accession
-	4.1347	0	Isolated from the medium by tributyrin as the sole carbon source	AQGM00000000.1
-	4.13565	0	A potential strain for (*R*,*R*)-2,3-butanediol production	ARYD00000000.1
SK19.001	3.92572	0	Isolated from soil in Wuxi (China)	AOFO00000000.1
AH159-1	3.99223	0	Isolated from mushroom in South Korea	JFBZ00000000.1
AP183	3.99136	0	Isolated from rhizosphere of cotton in the United States	JXAM00000000.1
GR4-5	4.1114	0	Isolated from soil in Gangwon (South Korea)	JYGH00000000.1
KACC 13105	3.88892	0	Isolated from rice rhizosphere soil in Cheongwon (South Korea)	JTKJ00000000.2
OB9	3.8614	0	Isolated from crude oil in Canada; produce lipopeptides, such as surfactins that act as immune-stimulators of the host plant and have gained importance in the fields of environmental bioremediation, food processing, and pharmaceuticals	LGAU00000000.1
B26	3.86876	0	Isolated from the bioenergy crop switchgrass in Canada; produce lipopeptides, such as surfactins that act as immunostimulators of the host plant and have gained importance in the fields of environmental bioremediation, food processing, and pharmaceuticals	LGAT00000000.1
NBIF-003	3.88167	0	Isolated from soil in Qingdao (China)	LJJY00000000.1
NRRL B-41580	4.03433	0	Isolated from river Velez in Malaga (Spain)	LLZC00000000.1
KACC 18228	3.92842	0	Isolated as rice endophyte in South Korea	LLZA00000000.1
NRRL B-4257	4.00626	0	Isolated from soil in Ness Ziona (Israel)	LLZB00000000.1
FKM10	3.92879	0	Isolated from the soil of apple rhizosphere in Shandong (China); A member of PGPR containing antimicrobial activity to some pathogen of soil-borne plant diseases, such as *Fusarium oxysporum*, *F. solani*, and *F. proliferatum*	LNTG00000000.1
RC218	3.85624	0	Isolated from wheat anthers as potential biocontrol agents against head blight causal agent *Fusarium graminearum* in Argentina; reduction of deoxynivalenol accumulation	LQCL00000000.1
AP214	4.03978	0	Isolated from soil in the United States	LSZM00000000.1
AP194	3.9894	0	Isolated from soil in the United States	LSZL00000000.1
CFSAN034338	4.05201	0	Isolated from agricultural soil in Canada	LYNA00000000.1
CFSAN034339	4.20953	0	Isolated from agricultural soil in Canada	LYNB00000000.1
CFSAN034340	4.06284	0	Isolated from agricultural soil in Canada	LYNC00000000.1
HJ18-4	4.11289	0	Isolated from fermented soybean paste in South Korea	MDCI00000000.1
CH13	3.88126	0	A plant beneficial bacterium isolated from chernozem soil of *Triticum aestivum* L. in Moldova	MPHE00000000.1
2A-2B	3.95861	0	Isolated from rhizosphere soil of a wild plant in Mexico	MLCV00000000.1
OEE1	4.07184	0	Isolated from *Olea europaea* in Tunisia	MZXS00000000.1

Table S2. Continued.

Strain	Genome size (Mb)	Plasmid	Description	GenBank Accession
SSBW-18	3.81082	0	Isolated from slow sand bio-filter in Poland	NBMN00000000.1
SSBW-2	3.92408	0		NBMQ00000000.1
SSBW-10	3.93708	0		NBMO00000000.1
SSBW-19	3.93315	0		NBMM00000000.1
SSBW-8	3.93612	0		NBMP00000000.1
NB91	3.84009	0	Isolated from external auditory canal of human (*Homo sapiens*) in Ningbo (China)	MTID00000000.1
KCTC 13012	4.03936	0	An antibiotic-Producing *Bacillus* isolated from river in Velez at Torredelmar (Spain) which exhibits a broad spectrum of antagonistic activity against bacteria and fungi and promotes plant growth	LHCC00000000.1
K26	4.00504	0	-	BDDG00000000.1
CBMB205	3.88526	0	-	FNER00000000.1
SB1216	3.81472	0	Isolated from agricultural soil in the United States	CP015417.1
W2	3.99751	0	A plant growth-promoting rhizobacterium isolated from saffron fields of Kashmir, India	JOKF00000000.1
SRCM100731	4.04106	0	Isolated from Kochujang in South Korea	LYUF00000000.1
SRCM100730	4.04125	0	Isolated from Kochujang in South Korea	LZZN00000000.1
V4	3.88601	0	Isolated from water in Tianjin (China)	MBDV00000000.1

Table S3. Antifungal activity of endophytic bacteria against the olive trees pathogens using the dual culture method.

	Rb	Pu	Rs	Na	B	N	Fs	Fo	C
**Control**	0.00	0.00	0.00	0.00	0.00	0.00	0.00	0.00	0.00
**10R3I**	86,14 ± 0.72^{b}	86,39 ± 0.26^{b}	16,22 ± 1.70^{n}	21.36 ± 0.02^{mn}	63.32 ± 0.39^{f}	25.89 ± 0.88^{m}	60.24 ± 0.59^{c}	54.27 ± 0.58^{g}	70.35 ± 0.69^{c}
**11R3I**	13,30 ± 2.25^{kl}	20,98 ± 0.11^{l}	20,89 ± 1.58^{m}	33.83 ± 1.04^{k}	0.00	0.00	29.46 ± 0.36^{h}	0.00	0.00
**12R3I**	63,13 ± 2.24^{d}	32,22 ± 0.45^{k}	28,37 ± 1.73^{j}	36.21 ± 0.95^{j}	44.10 ± 2.54^{h}	0.00	54.92 ± 0.41^{d}	13.58 ± 0.65^{o}	64.91 ± 0.32^{e}
**13R3I**	26,50 ± 0.2^{h}	89,03 ± 0.07^{a}	50,44 ± 0.58^{h}	74.03 ± 0.32^{bc}	31.96 ± 1.37^{i}	23.14 ± 0.31^{m}	0.00	8.29 ± 1.28^{q}	69.75 ± 1.20^{c}
**14R3I**	0.00	20,98 ± 1.01^{l}	35,92 ± 1.70^{i}	28.23 ± 0.45^{l}	0.00	0.00	12.57 ± 0.85^{m}	0.00	4.32 ± 0.03^{r}
**15R3I**	30,42 ± 0.91^{g}	51,83 ± 0.21^{h}	19,20 ± 1.02^{m}	19.74 ± 0.09^{n}	28.45 ± 0.21^{i}	11.5 ± 0.67^{o}	28.62 ± 1.22^{h}	60.38 ± 0.31^{ef}	0.00
**17B1I**	51,89 ± 2.88e	73,17 ± 0.39^{de}	25,49 ± 0.91^{kl}	32.51 ± 1.23^{k}	82.37 ± 0.69^{bc}	72.15 ± 1.33^{c}	64.91 ± 0.09^{b}	57.36 ± 0.97^{f}	76.22 ± 0.52^{b}
**OEE1**	61,09 ± 0.65^{d}	56,47 ± 3.55^{fg}	84,30 ± 1.15^{a}	85.78 ± 0.42^{a}	86.38 ± 0.54^{a}	82.54 ± 0.25^{a}	82.42 ± 0.15^{b}	42.86 ± 0.64^{i}	81.47 ± 0.47^{a}
**1B2I**	0.00	45,10 ± 1.81^{i}	0,00	0.88 ± 0.63^{s}	0.00	0.00	0.00	13.82 ± 0.09^{o}	4.61 ± 0.61^{r}
**1C3I**	56,72 ± 1.01^{d}	7,49 ± 0.23^{no}	7,34 ± 1.30^{o}	16.06 ± 0.33^{o}	31.91 ± 2.14^{i}	0.00	26.48 ± 0.37^{i}	37.60 ± 0.22^{j}	44.35 ± 0.32^{j}
**1F1I**	0,00	9,00 ± 0.01^{n}	0,00	0.00	0.00	0.00	4.36 ± 0.94^{o}	10.21 ± 0.03^{p}	0.00
**1F2I**	35,52 ± 0.44^{fg}	39,41 ± 3.01^{j}	45,17 ± 0.03^{hi}	58.57 ± 0.41^{f}	0.00	62.33 ± 0.89^{fg}	2.58 ± 0.60^{p}	46.24 ± 0.72^{h}	61.75 ± 0.08^{fg}
**1R3I**	79,78 ± 1^{c}	26,43 ± 0.11^{k}	68,93 ± 0.76^{e}	69.11 ± 1.12^{c}	83.20 ± 0.085^{b}	64.38 ± 0.37^{e}	46.21 ± 0.03^{f}	70.59 ± 0.83^{c}	47.21 ± 0.64^{hi}
**1T1I**	20,81 ± 0.04^{j}	61,78 ± 0.49^{f}	69,81 ± 0.24^{d}	72.65 ± 0.37^{c}	8.51 ± 0.61^{m}	35.26 ± 0.14^{jk}	28.24 ± 0.77^{hi}	66.35 ± 0.23^{d}	48.91 ± 0.01^{h}
**20R3I**	1,00 ± 1.73^{m}	11,15 ± 0.34^{n}	0,00	0.00	43.71 ± 0.12^{h}	0.00	0.00	21.34 ± 0.94^{m}	0.00
**20R3I'**	0,00	19,49 ± 1.43^{l}	0,00	0.00	0.00	29.20 ± 2.97^{l}	0.00	0.00	5.36 ± 1.24^{r}
**2B1F**	23,10 ± 0.33^{i}	15,55 ± 0.84^{m}	2,52 ± 1.03^{p}	6.46 ± 0.52^{q}	32.18 ± 0.66^{i}	2.31 ± 2.32^{q}	73.26 ± 0.26^{a}	0.00	67.92 ± 0.68^{d}
**2B1I**	74,03 ± 0.57^{cd}	27,74 ± 0.64^{k}	66,78 ± 1.76^{f}	69.91 ± 0.69^{c}	68.92 ± 0.73^{e}	78.31 ± 0.90^{ab}	59.28 ± 0.42^{c}	66.84 ± 0.56^{d}	46.80 ± 0.07^{i}
**2B2I**	0,00	3,00 ± 0.1^{o}	0,00	0.00	0.00	24.59 ± 0.38^{m}	0.00	0.00	0.00
**2C3I**	51,75 ± 2.31^{e}	7,52 ± 0.15^{no}	0,00	2.20 ± 0.35^{rs}	54.91 ± 2.39^{g}	46.2 ± 0.66^{i}	27.13 ± 0.83^{i}	0.00	33.56 ± 0.28^{l}
**2F1I**	0,00	8,15 ± 0.56^{no}	25,31 ± 1.03^{kl}	24.11 ± 1.08^{m}	31.96 ± 0.48^{i}	19.67 ± 0.07^{n}	20.61 ± 0.68^{k}	0.00	10.82 ± 0.33^{p}
**2R3I**	20,02 ± 2.31^{j}	76,02 ± 0.06^{d}	49,20 ± 1.45^{h}	53.98 ± 0.94^{g}	16.20 ± 0.90^{k}	37.29 ± 0.58^{j}	24.66 ± 0.49^{j}	41.09 ± 0.38^{i}	7.34 ± 0.19^{q}
**2T1I**	0,00	87,81 ± 0.68^{a}	24,28 ± 1.21^{l}	36.90 ± 0.67^{j}	12.50 ± 1.50^{l}	0.00	39.88 ± 1.08^{g}	60.75 ± 2.06^{ef}	0.00
**3B1I**	58,45 ± 0.9^{d}	14,25 ± 0.15^{m}	69,54 ± 0.21^{de}	69.66 ± 0.28^{c}	81.60 ± 0.11^{c}	76.14 ± 0.21^{b}	38.09 ± 0.16^{g}	57.92 ± 0.33^{f}	69.83 ± 0.21^{c}
**3B2I**	0,00	68,78 ± 1.74^{e}	70,54 ± 1.82^{d}	67.82 ± 0.84^{d}	0.00	0.00	51.40 ± 0.73^{e}	74.36 ± 0.49^{b}	9.29 ± 0.43^{p}
**3R3I**	5,46 ± 0.46^{lm}	52,67 ± 3.04^{g}	53,86 ± 0.85^{g}	58.21 ± 0.12^{f}	0.00	46.97 ± 0.96^{i}	23.50 ± 0.89^{j}	0.00	69.84 ± 0.97^{c}
**3T1I**	11,27 ± 0.04^{l}	20,16 ± 0.66^{l}	17,49 ± 1.39^{n}	38.99 ± 1.61^{i}	0.00	18.97 ± 0.72^{n}	24.58 ± 0.44^{j}	3.21 ± 0.81^{r}	0.00
**4B1I**	0,00	0,00	53,02 ± 0.61^{g}	0.00	25.92 ± 0.82^{ij}	0.00	0.00	44.67 ± 0.66^{h}	0.00
**4B2I**	8,77 ± 0.03^{l}	6,97 ± 1.15^{no}	65,14 ± 1.33^{f}	71.17 ± 0.24^{c}	0.00	62.74 ± 0.02^{fg}	0.00	18.63 ± 0.32^{mm}	0.00
**4C3I**	14,13 ± 2.47^{k}	61,14 ± 0.8^{f}	0,97 ± 0.52^{pq}	11.94 ± 0.61^{pq}	19.82 ± 1.86^{j}	0.00	0.00	31.57 ± 0.91^{k}	16.80 ± 0.38^{n}

**4R3I**	0,00	3,82 ± 0.05^{o}	69,93 ± 0.48^{d}	70.02 ± 1.33^{c}	0.00	49.65 ± 0.34^{h}	8.03 ± 0.04^{n}	0.00	58.28 ± 0.45^{g}
**5B2I**	0,00	3,91 ± 0.6^{o}	0,00	57.44 ± 0.92	21.69 ± 0.49^{j}	37.19 ± 1.69^{j}	0.00	0.00	22.64 ± 0.97^{m}
**5R3I**	42,88 ± 0.94^{f}	7,27 ± 0.81^{no}	2,30 ± 1.00^{p}	12.6 ± 0.09^{p}	0.00	0.00	9.38 ± 0.13^{n}	59.01 ± 0.64^{f}	0.00
**6B1I**	0,00	31,56 ± 4.1^{k}	0,86 ± 1.49^{pq}	8.14 ± 0.25^{q}	22.73 ± 0.72^{j}	6.01 ± 0.72^{p}	0.94 ± 0.81^{q}	0.00	0.00
**6B2I**	20,88 ± 0.16^{j}	82,78 ± 0.83^{c}	41,74 ± 0.85^{hi}	45.58 ± 0.86^{h}	0.00	0.00	46.85 ± 0.60^{f}	17.91 ± 0.82^{n}	22.19 ± 0.83^{m}
**6R3I**	42,05 ± 0.89^{f}	75,60 ± 0.04^{d}	73,39 ± 0.42^{c}	75.89 ± 2.01^{bc}	0.00	51.48 ± 0.11^{h}	16.08 ± 0.72^{l}	20.09 ± 0.74^{m}	0.00
**7B1I**	0,00	19,43 ± 1.01^{l}	67,84 ± 0.58^{ef}	62.63 ± 0.43^{e}	21.65 ± 0.25^{j}	67.33 ± 0.91^{d}	0.00	0.00	40.97 ± 0.09^{k}
**7R3I**	90,93 ± 1.02^{a}	21,67 ± 0.41^{l}	26,92 ± 1.73^{k}	36.55 ± 0.66^{j}	62.82 ± 0.83^{f}	58.21 ± 0.83^{g}	0.00	24.94 ± 0.12^{l}	13.50 ± 0.50^{o}
**8B1I**	41,70 ± 1.65^{f}	68,96 ± 0.43^{e}	0,73 ± 0.15^{pq}	4.63 ± 0.28^{r}	0.00	63.48 ± 0.41^{e}	40.09 ± 0.36^{g}	61.83 ± 1.36^{e}	7.21 ± 0.26^{q}
**8R3I**	0,00	13,52 ± 0.22^{n}	0,99 ± 0.2^{pq}	7.41 ± 0.94^{q}	0.00	0.00	12.30 ± 2.09^{m}	0.00	4.07 ± 0.05^{r}
**9R3I**	29,15 ± 0.33^{g}	44,41 ± 1.66^{i}	77,24 ± 0.03^{b}	79.38 ± 1.20^{b}	0.00	34.81 ± 0.09^{k}	0.00	78.31 ± 0.64^{a}	0.00
**B2Fr**	47,69 ± 0.3^{ef}	29,07 ± 0.55^{k}	66,80 ± 1.85^{f}	54.01 ± 0.03^{g}	75.07 ± 1.22^{d}	74.95 ± 0.65^{bc}	26.04 ± 0.57^{i}	61.99 ± 0.91^{e}	43.70 ± 0.93^{j}

Rb: *Rhizoctonia bataticola* HQ392809.1, Rs: *Rhizoctonia solani* KU863546, Na: *Neofusicoccum australe* EU375516.1, N: *Nigrospora* sp. JN 207298.1, B: *Botryospheria* sp., Fs: *Fusarium solani* FJ874633.1, Pu: *Pythium ultimum*, Fo: *Fusarium oxysporum* JN400698.1, C: *Cylindrocarpon* sp.

Table S4. *in vitro* evaluation of beneficial traits of the strain OEE1.

**Antimicrobial traits**					**PGP activities**					**Endophytic traits**		
**Protease**		**Chitinase**	**Glucanase**	**HCN production**	**Siderophore production**	**IAA production**		**Phosphate solubilization**	**Nitrogen fixation**	**Cellulase**	**Pectinase**	**Amylase**
**Gelatin hydrolysis**	**Casein hydrolysis**	+	+	+	+	**L-Trp (+)**	**L-Trp (-)**	+	+	+	+	+
+	+					+	+					

Figure S1. Inhibition of fungal growth by volatile organic compounds (VOCs) of *Bacillus velezensis* strain OEE1.

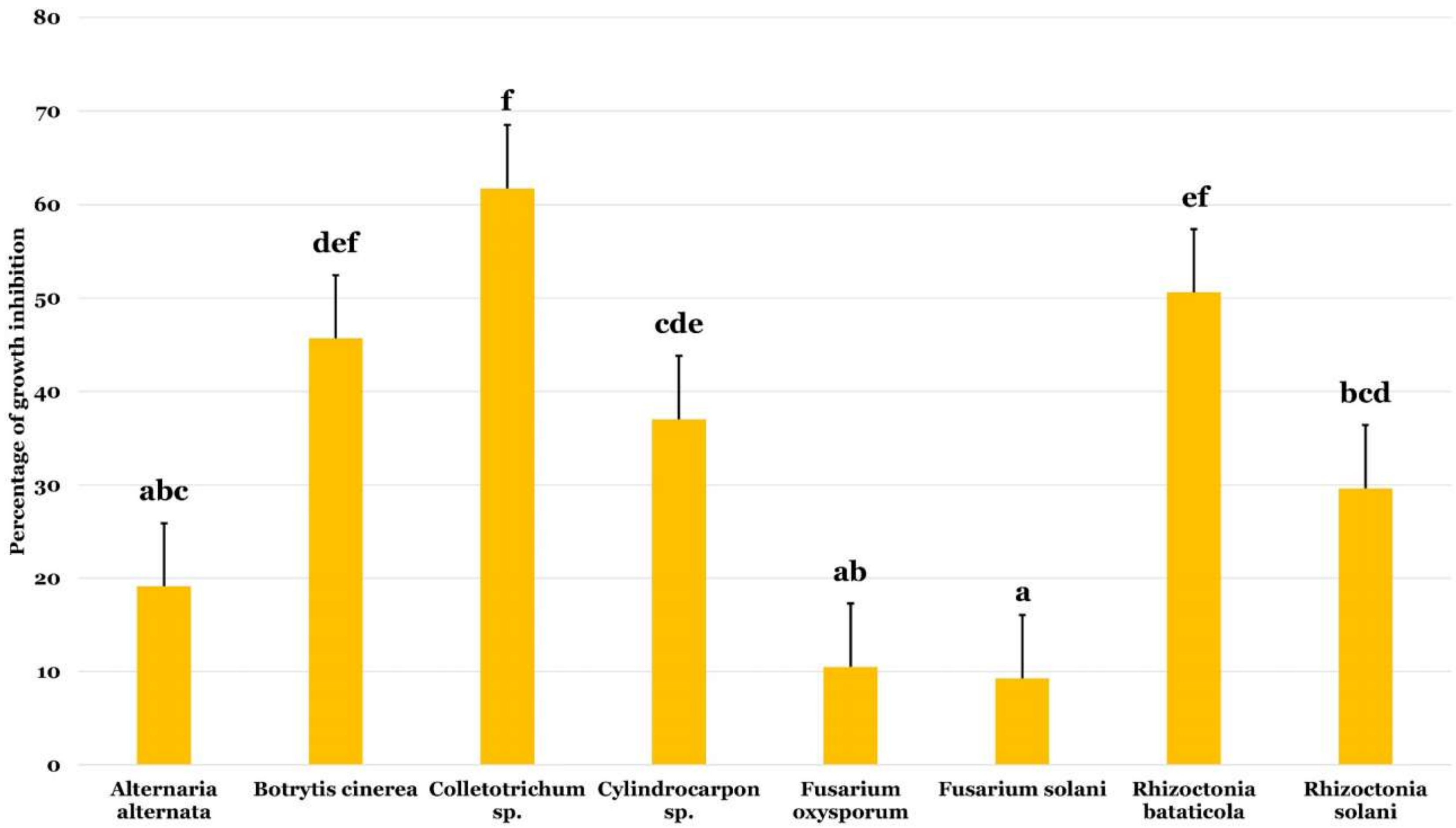

Figure S2. Genome-to-Genome Distance Calculation (GGDC) values between each indicated isolate were calculated with GGDC 2 web-based program and showed 3 species candidates based on 70% similarity thresholds.

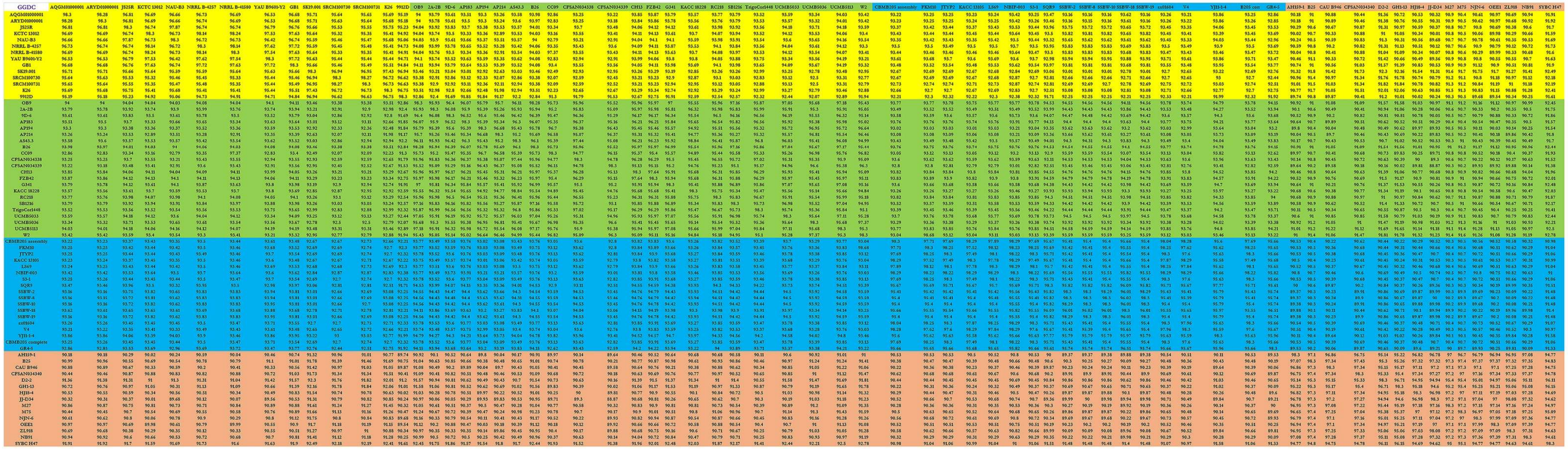

Figure S3. Average nucleotide identity (ANI) values between each indicated isolate were calculated with EzBiocloud web-based program and showed 3 species candidates based on 95% similarity thresholds.

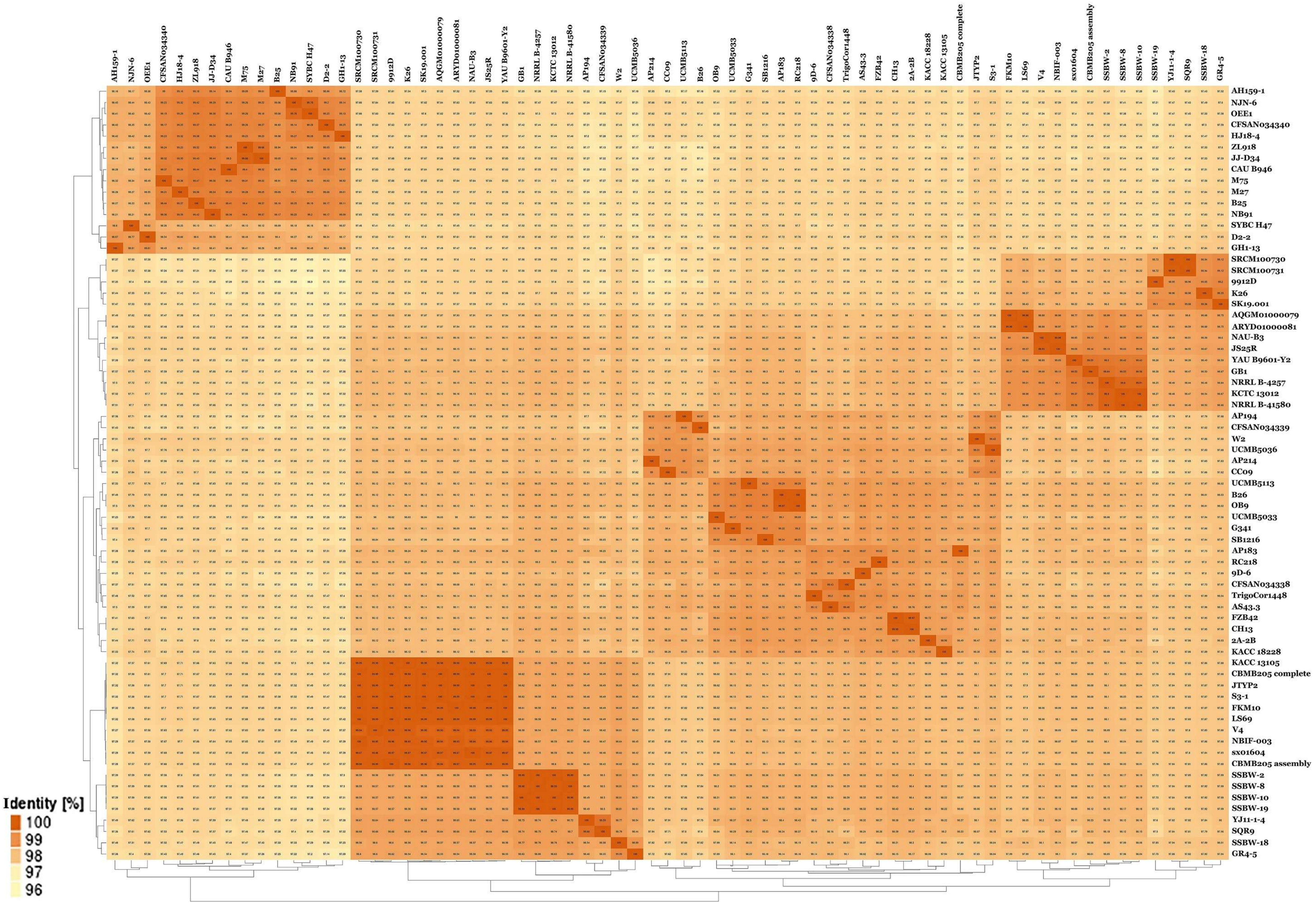

Figure S4. (**a**) Number of discovered secondary metabolites; (**b**) Statistically significant linear relationship between genome sizes and antiSMASH total hits ($p < 0.05$); (**c**) Statistically significant linear relationship between genome sizes and PRISM total hits.

a

b

c

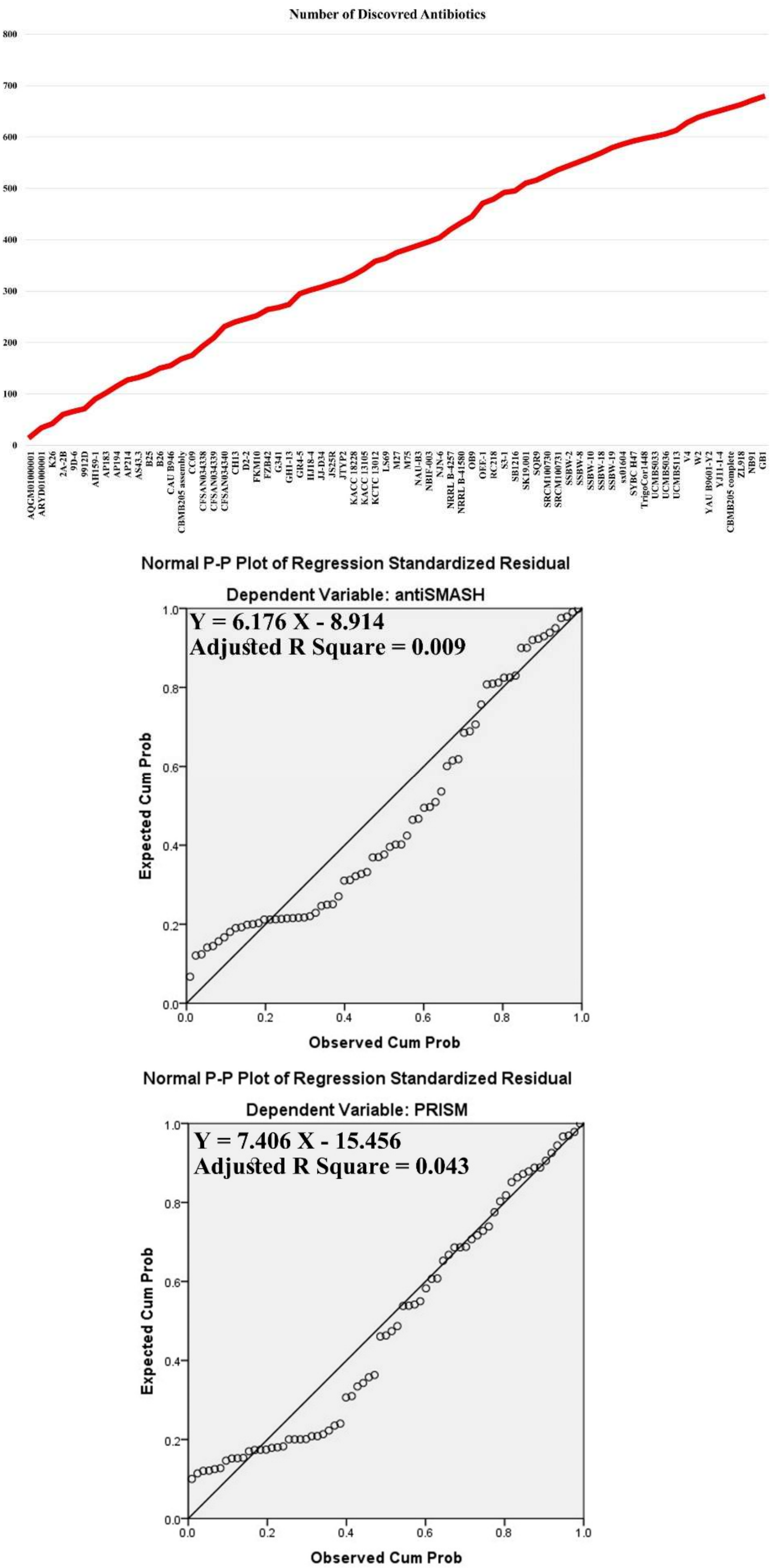

Figure S5. Significant linear relationship between genome sizes and accessory genome antiSMASH total hits ($p < 0.05$).

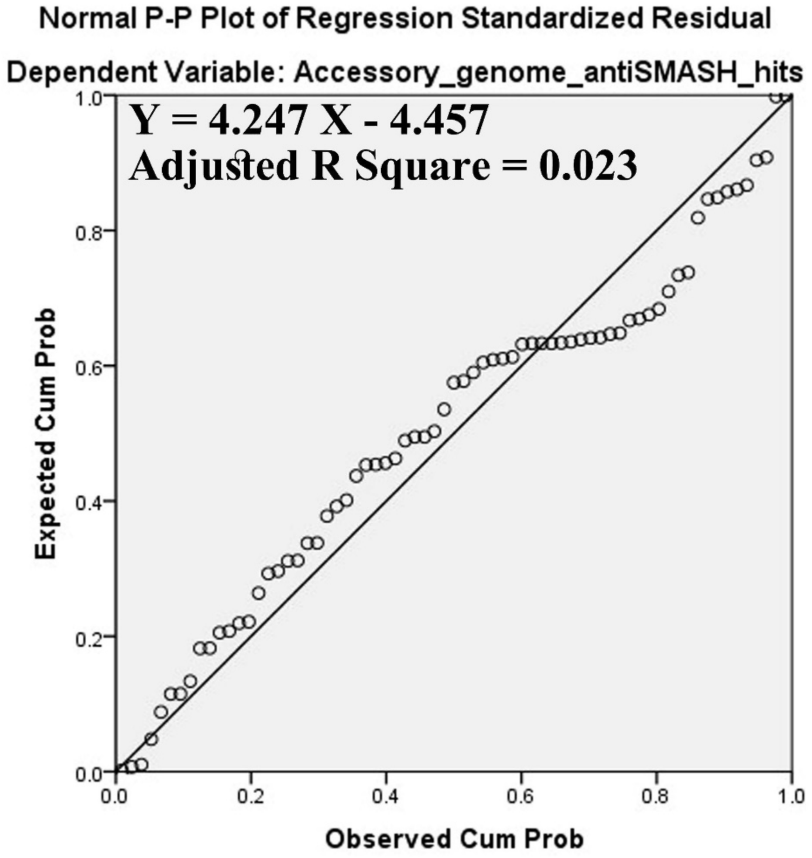

Supplement: Supplementary file 1 [file microorganisms-07-00314-s001.pdf]
